# Supplementary material for: High potential contribution of intercropping to soybean and maize self-sufficiency in Europe
Source: Nat Commun. 2026 Oct 6;17:11389. doi: 10.1038/s41467-026-78206-4 (PMC13736886; doi:10.1038/s41467-026-78206-4)
Supplement: Supplementary file 2 [file 41467_2026_78206_MOESM2_ESM.pdf]

nature portfolio

Corresponding author(s): Mathilde CHEN

Last updated by author(s): Jun 22, 2026

Reporting Summary

Nature Portfolio wishes to improve the reproducibility of the work that we publish. This form provides structure for consistency and transparency in reporting. For further information on Nature Portfolio policies, see our Editorial Policies and the Editorial Policy Checklist.

Statistics

For all statistical analyses, confirm that the following items are present in the figure legend, table legend, main text, or Methods section.

n/a	Confirmed	
☐	☒	The exact sample size (*n*) for each experimental group/condition, given as a discrete number and unit of measurement
☐	☒	A statement on whether measurements were taken from distinct samples or whether the same sample was measured repeatedly
☒	☐	The statistical test(s) used AND whether they are one- or two-sided<br>*Only common tests should be described solely by name; describe more complex techniques in the Methods section.*
☐	☒	A description of all covariates tested
☐	☒	A description of any assumptions or corrections, such as tests of normality and adjustment for multiple comparisons
☐	☒	A full description of the statistical parameters including central tendency (e.g. means) or other basic estimates (e.g. regression coefficient) AND variation (e.g. standard deviation) or associated estimates of uncertainty (e.g. confidence intervals)
☒	☐	For null hypothesis testing, the test statistic (e.g. *F*, *t*, *r*) with confidence intervals, effect sizes, degrees of freedom and *P* value noted<br>*Give P values as exact values whenever suitable.*
☒	☐	For Bayesian analysis, information on the choice of priors and Markov chain Monte Carlo settings
☒	☐	For hierarchical and complex designs, identification of the appropriate level for tests and full reporting of outcomes
☐	☒	Estimates of effect sizes (e.g. Cohen's *d*, Pearson's *r*), indicating how they were calculated

Our web collection on statistics for biologists contains articles on many of the points above.

Software and code

Policy information about availability of computer code

Data collection	References and access to the dataset:<br>- Historical yields: Iizumi, T., Sakai, T. The global dataset of historical yields for major crops 1981–2016. Sci Data 7, 97 (2020). https://doi.org/10.1038/s41597-020-0433-7 ; the dataset is accessible here: https://doi.pangaea.de/10.1594/PANGAEA.909132<br>- Historical climate data: Hersbach, H., Bell, B., Berrisford, P., Biavati, G., Horányi, A., Muñoz Sabater, J., Nicolas, J., Peubey, C., Radu, R., Rozum, I., Schepers, D., Simmons, A., Soci, C., Dee, D., Thépaut, J-N. (2023): ERA5 hourly data on single levels from 1940 to present. Copernicus Climate Change Service (C3S) Climate Data Store (CDS), DOI: 10.24381/cds.adbb2d47 (Accessed in February 2023) ; the data is accessible here: https://cds.climate.copernicus.eu/datasets/reanalysis-era5-single-levels?tab=download<br>- Irrigation fraction: Yu, Q., You, L., Wood-Sichra, U., Ru, Y., Joglekar, A. K. B., Fritz, S., Xiong, W., Lu, M., Wu, W., and Yang, P.: A cultivated planet in 2010 – Part 2: The global gridded agricultural-production maps, Earth Syst. Sci. Data, 12, 3545–3572, https://doi.org/10.5194/essd-12-3545-2020, 2020. SPAM2010 can be downloaded via an open-data repository (DOI: https://doi.org/10.7910/DVN/PRFF8V; IFPRI, 2019).<br>- Crop calendars provided by the Agricultural Market Information System: www.amis-outlook.org/amis-about/calendars/soybeancal/en/<br>- Gridded N fertilizers rates: Nguyen, T.H., Tang, F.H.M., Conchedda, G. et al. NPKGRIDS: a global georeferenced dataset of N, P2O5, and K2O fertilizer application rates for 173 crops. Sci Data 11, 1179 (2024). https://doi.org/10.1038/s41597-024-04030-4
Data analysis	Packages (and their version) required for the analyses:<br>- for data management: tidyverse (v2.0.0), stringr (v1.6.0), lubridate (v1.9.3), CCMHr<br>- for graphics: ggplot2 (v4.0.3), cowplot (v1.1.3), viridis (v0.6.5)<br>- for spatial data management and maps production: terra (v1.8-29), raster (v3.6-31), rnaturalearth (v1.0.1), rnaturalearthdata (v1.0.0), sf (v1.0-19), sp (v2.2-0), rworldmap (v1.3-8), rmapshaper (v0.5.0), tidygeocoder (v1.0.5), metR (v0.18.0)<br>- for parallelization of the analyses: parallel (4.4.2), doParallel (1.0.17), foreach (1.5.2)

- for random forest models fitting: ranger (0.17.0)
All analyses were done using R version 4.4.2.

For manuscripts utilizing custom algorithms or software that are central to the research but not yet described in published literature, software must be made available to editors and reviewers. We strongly encourage code deposition in a community repository (e.g. GitHub). See the Nature Portfolio guidelines for submitting code & software for further information.

Data

Policy information about availability of data

All manuscripts must include a data availability statement. This statement should provide the following information, where applicable:

- Accession codes, unique identifiers, or web links for publicly available datasets
- A description of any restrictions on data availability
- For clinical datasets or third party data, please ensure that the statement adheres to our policy

References and access to the data used for the study:
- Historical yields: Iizumi, T., Sakai, T. The global dataset of historical yields for major crops 1981–2016. Sci Data 7, 97 (2020). https://doi.org/10.1038/s41597-020-0433-7 ; the dataset is accessible here: https://doi.pangaea.de/10.1594/PANGAEA.909132
- Historical climate data: Hersbach, H., Bell, B., Berrisford, P., Biavati, G., Horányi, A., Muñoz Sabater, J., Nicolas, J., Peubey, C., Radu, R., Rozum, I., Schepers, D., Simmons, A., Soci, C., Dee, D., Thépaut, J-N. (2023): ERA5 hourly data on single levels from 1940 to present. Copernicus Climate Change Service (C3S) Climate Data Store (CDS), DOI: 10.24381/cds.adbb2d47 (Accessed in February 2023) ; the data is accessible here: https://cds.climate.copernicus.eu/datasets/reanalysis-era5-single-levels?tab=download
- Irrigation fraction: Yu, Q., You, L., Wood-Sichra, U., Ru, Y., Joglekar, A. K. B., Fritz, S., Xiong, W., Lu, M., Wu, W., and Yang, P.: A cultivated planet in 2010 – Part 2: The global gridded agricultural-production maps, Earth Syst. Sci. Data, 12, 3545–3572, https://doi.org/10.5194/essd-12-3545-2020, 2020. SPAM2010 can be downloaded via an open-data repository (DOI: https://doi.org/10.7910/DVN/PRFF8V; IFPRI, 2019).
- Crop calendars provided by the Agricultural Market Information System: www.amis-outlook.org/amis-about/calendars/soybeancal/en/
- Gridded N fertilizers rates: Nguyen, T.H., Tang, F.H.M., Conchedda, G. et al. NPKGRIDS: a global georeferenced dataset of N, P2O5, and K2O fertilizer application rates for 173 crops. Sci Data 11, 1179 (2024). https://doi.org/10.1038/s41597-024-04030-4

Access to the processed data:
The processed data are available in the "Maize-Soybean Intercropping in Europe" repository, available at: https://github.com/MathildeChen/Maize_Soybean_Intercropping_Europe.

Research involving human participants, their data, or biological material

Policy information about studies with human participants or human data. See also policy information about sex, gender (identity/presentation), and sexual orientation and race, ethnicity and racism.

Reporting on sex and gender	*Use the terms sex (biological attribute) and gender (shaped by social and cultural circumstances) carefully in order to avoid confusing both terms. Indicate if findings apply to only one sex or gender; describe whether sex and gender were considered in study design; whether sex and/or gender was determined based on self-reporting or assigned and methods used. Provide in the source data disaggregated sex and gender data, where this information has been collected, and if consent has been obtained for sharing of individual-level data; provide overall numbers in this Reporting Summary. Please state if this information has not been collected. Report sex- and gender-based analyses where performed, justify reasons for lack of sex- and gender-based analysis.*
Reporting on race, ethnicity, or other socially relevant groupings	*Please specify the socially constructed or socially relevant categorization variable(s) used in your manuscript and explain why they were used. Please note that such variables should not be used as proxies for other socially constructed/relevant variables (for example, race or ethnicity should not be used as a proxy for socioeconomic status). Provide clear definitions of the relevant terms used, how they were provided (by the participants/respondents, the researchers, or third parties), and the method(s) used to classify people into the different categories (e.g. self-report, census or administrative data, social media data, etc.) Please provide details about how you controlled for confounding variables in your analyses.*
Population characteristics	*Describe the covariate-relevant population characteristics of the human research participants (e.g. age, genotypic information, past and current diagnosis and treatment categories). If you filled out the behavioural & social sciences study design questions and have nothing to add here, write "See above."*
Recruitment	*Describe how participants were recruited. Outline any potential self-selection bias or other biases that may be present and how these are likely to impact results.*
Ethics oversight	*Identify the organization(s) that approved the study protocol.*

Note that full information on the approval of the study protocol must also be provided in the manuscript.

Field-specific reporting

Please select the one below that is the best fit for your research. If you are not sure, read the appropriate sections before making your selection.

☒ Life sciences ☐ Behavioural & social sciences ☐ Ecological, evolutionary & environmental sciences

For a reference copy of the document with all sections, see nature.com/documents/nr-reporting-summary-flat.pdf

Life sciences study design

All studies must disclose on these points even when the disclosure is negative.

Sample size	To build the soybean and maize yield training datasets, we selected grid cells from locations representative of major production environments in the European Union and worldwide. For soybean, observational data within Europe were only available for Italy; therefore, additional grid cells were included from major soybean-producing countries (Argentina, Brazil, Canada, China, India, and the United States) to ensure that the training dataset captured a broad range of climatic and management conditions relevant to soybean production. For maize, grid cells from the European Union, the United States, and Northern China were included. The latter two regions were selected because their maize growing seasons and management practices are broadly comparable to those found in the EU.Within these regions, only grid cells with substantial crop presence were retained, defined as locations where the harvested area of soybean or maize individually represented at least 1% of total cropland. Crop-specific harvested area fractions were obtained from the M3 crop mask.The final training datasets comprised 3,286 sites (116,516 site-years) for soybean and 2,064 sites (72,964 site-years) for maize. The number of sites was therefore determined by the availability of yield observations within the selected production regions and by the crop area threshold applied to ensure meaningful crop representation. These datasets encompass a wide range of environmental and management conditions and contain tens of thousands of site-years, providing sufficient variability and sample size for robust model training and evaluation. After model development, annual rainfed soybean and maize yields in pure stands were projected for 2,699 sites across the EU over the 2000–2023 period, corresponding to 64,776 site-years.
Data exclusions	The data were taken in major-producing areas of the crops for which we built predictive models.
Replication	In the development of the model, the preditive accuracy was assessed based on 2 cross-validation stratgies. Several sensitivity analyses were performed to assess the robustness of the results according to various factors. See the Methods section of the paper for further details.
Randomization	Allocation of samples into experimental groups was not applicable to this study because no experimental treatments or intervention groups were defined. The study was based on the analysis of existing and modeled datasets, and therefore no randomization or group allocation procedures were performed.
Blinding	Blinding was not relevant to this study because it did not involve an experimental design, treatment allocation, or intervention groups. The analyses were based exclusively on existing and modeled datasets, and therefore no investigators were assigned to groups during data collection or analysis.

Reporting for specific materials, systems and methods

We require information from authors about some types of materials, experimental systems and methods used in many studies. Here, indicate whether each material, system or method listed is relevant to your study. If you are not sure if a list item applies to your research, read the appropriate section before selecting a response.

Materials & experimental systems

n/a	Involved in the study	
☒	☐	Antibodies
☒	☐	Eukaryotic cell lines
☒	☐	Palaeontology and archaeology
☒	☐	Animals and other organisms
☒	☐	Clinical data
☒	☐	Dual use research of concern
☒	☐	Plants

Methods

n/a	Involved in the study	
☒	☐	ChIP-seq
☒	☐	Flow cytometry
☒	☐	MRI-based neuroimaging

Plants

Seed stocks	Not appropriate (modelling study).
Novel plant genotypes	Not appropriate (modelling study).
Authentication	Not appropriate (modelling study).
