# Supplementary material for: High potential contribution of intercropping to soybean and maize self-sufficiency in Europe
Source: Nat Commun. 2026 Oct 6;17:11389. doi: 10.1038/s41467-026-78206-4 (PMC13736886; doi:10.1038/s41467-026-78206-4)
Supplement: Supplementary file 3 [file 41467_2026_78206_MOESM3_ESM.pdf]

nature portfolio

Peer Review File

High potential contribution of intercropping to soybean and maize self-sufficiency in Europe

Corresponding Author: Dr Mathilde Chen

Parts of this Peer Review File have been redacted as indicated to remove third-party material.

This file contains all reviewer reports in order by version, followed by all author rebuttals in order by version.

Version 0:

Reviewer comments:

Reviewer #1

(Remarks to the Author)
Review of Chen et al. “High potential contribution of intercropping to soybean and maize self-sufficiency in Europe”

I have read this paper with anticipation and interest because I share the authors' belief that intercropping maize and soybean is an overlooked opportunity for increasing production of maize and soybean in Europe while moderating inputs. The topic is important, and I feel the basis conclusions of the paper are cautious enough to be right. Nevertheless the analysis left me unsatisfied and unconvinced. The prime reason for this is that the results depend critically not so much on the machine learning that the authors have done (highly competent, as far as I can judge), but on the values for the land equivalent ratio (LER) that were obtained in a previous study (Xu et al., 2020). The machine learning (ML) focuses on the yields of soybean and maize and was mostly used to select regions where soybean yields are high enough (>1 t/ha) to grow soybean as sole crop or as an intercrop with maize. Then the ML models are used to predict yields in the sole crops, and the average LER from Xu et al. (2020) is used to obtain the intercrop yield. It is no surprise that the results of the study suggest that large improvements in self-sufficiency in soybean production can be achieved by growing maize-soybean intercrops on a large area. This conclusions follows straight from the assumed LER which was obtained from Xu et al. (2020). However, whether this LER will materialize in Europe is highly uncertain due to lack of experiments. The effect of climatic conditions and management on the LER are likely to be important. The work by Xu et al. (2020) but also by Yu et al. (2015) and by Li et al. (2020) showed the large importance of temporal complementarity between the crop species, i.e. relay intercropping has greater production advantages than simultaneous intercropping, particularly at higher input levels (Li et al., 2020). I would have expected for an analysis of this kind that an attempt would have been made to assess whether temporal complementarity would be obtainable given the heat sums available across Europe. Moreover, meta-analyses have shown the high importance of N input for LER (e.g. Yu et al., 2016, Liu et al., 2024). Generally, in cereal-legume intercropping, the LER decreases with N input as the mechanism for complementarity (different sources of N for the two crop species) is taken away. With no temporal complementarity and contemporary western-European N input into the crop system, I would not expect a land equivalent ratio for maize-soybean intercropping substantially above 1.10, though it could be higher under conditions of lower fertilization (but at low yield levels) and it could be substantially higher if substantial temporal complementarity can be achieved. I feel the study would be a lot more convincing if somehow these effects of N input and temporal complementarity would have been incorporated to generate projections.

I am not saying the results are wrong. I agree with the findings, I just feel that the most important issue determining the prospects of maize-soybean intercropping is not sufficiently addressed and leaves the reader with large uncertainty. Currently uncertainty is addressed with a sensitivity analysis, which could be fine in itself, but I feel it is not satisfactory as the LER is the factor of overriding importance influencing the yield advantages that can be attained and that are the core of the paper. The LER depends on the growing conditions and management. The ML models are interesting but they only address the yields in sole cropping and do not address the extent to which intercropping can achieve complementarity and overyielding.

Li CJ, Hoffland E, Kuyper Th W, Yu Y, Zhang CC, Li HG, Zhang FS, van der Werf W (2020) Syndromes of production impact yield gains in intercropping. Nature Plants
6, 653–660. http://dx.doi.org/10.1038/s41477-020-0680-9
Liu YL, Stomph TJ, Zhang FS, Li CJ, van der Werf W (2024) Nitrogen input strategies impact fertilizer nitrogen saving by

intercropping: A global meta-analysis. Field Crops Research 318: 109607. https://doi.org/10.1016/j.fcr.2024.109607
Xu Z, Li CJ, Zhang CC, Yang Y, van der Werf W, Zhang FS (2020) Intercropping maize and soybean increases efficiency of land and fertilizer nitrogen use; A meta-analysis. Field Crops Research 246: 107661. http://dx.doi.org/10.1016/j.fcr.2019.107661
Yu Y, Stomph TJ, Makowski D, van der Werf W (2015) Temporal niche differentiation increases the land equivalent ratio of annual intercrops: a meta-analysis. Field Crops Research 184, 133–144. http://dx.doi.org/10.1016/j.fcr.2015.09.010
Yu Y, Stomph TJ, Makowski D, Zhang L, van der Werf W (2016) A meta-analysis of relative crop yields in cereal/legume mixtures suggests options for management. Field Crops Research 198: 269-279. http://dx.doi.org/10.1016/j.fcr.2016.08.001

Detailed comments
18-21 This statement in the abstract is highly contingent on the value of LER used. The estimate from Xu et al. (2020) is high. It does reflect the literature, but new experiments are needed to assess the current yield advantages. In experiments I have been involved in in China under current conditions, the LER was about 1.10 (not responsive to N input because of high N stock in the soil). LERs from relay intercropping are likely (substantially) higher, but the paper provides no evidence that relay intercropping would be achievable in Europe given the current climatic conditions.
52-54 The paper cites a paper by Wu et al. (2023) stating that “In China, intercropping maize with soybean was found to double soybean production”. I initially misread this statement thinking you meant the yield per ha was doubled and therefore checked the source. Please clarify to avoid such misreading.
62-70 The authors bring up an interesting point in differences in sole crop yields across Europe. However, I don’t see clearly how this would affect the land saving or production increases from intercropping because these depend on the complementarity (the LER), not on the monocrop yields. In other words, I have the impression that we would obtain very similar results if we would just apply the LER to the European average yields of maize and soybean on the area that is judged suitable for growing this combination, without caring about the regional differences in absolute yield. Am I missing something?
96 Would soybean yields of 1.0 t/ha be judged acceptable in a European context? It seems low, but it may be ok.
103 equal to or higher than
119-128 This section is a bit hard to follow. I think you mean that the intercrop would be grown on the same area as soybean is currently grown. But perhaps you mean that the intercrop is grown in areas where predicted soybean yield is at least 1.0 t/ha. Please clarify.
120-124 I probably misread the sentence, but how can you maintain maize yields if an intercrop maize-soybean is grown in the highest yielding soybean areas? Soybean areas have no maize yield at all. Please clarify. You must mean something else than I read.
132-139 These results feel a bit “deja-vu” for people who are familiar with the LER concept. They follow straight from the LER. But the LER is uncertain.
149-150 I feel some reservation about these results because they depend totally on the assumed LER value which may not be achievable across Europe. Studies in China have shown vast variation in LER, mainly driven by temporal complementarity (ongoing work by Tao et al., but see also Li et al. (2020) and Xu et al. (2020)).
158 provided
157-172 The sensitivity analysis is helpful, but it mixes somehow the effect of LER and of total area used for production of maize and soybean. I mean: in Fig. 4 we see that at the lowest pLER of soybean (0.3) and at the lowest frequency of maize-soybean in the rotation (1/7), the self-sufficiency for soybean is still increased from ~15 to >25%, which is a vast increase. But at this LER, there is also a production increase for maize while the total LER is only 1.09 (0.3 + 0.79). As a reader I do not get a good enough understanding of the areas used in the scenario and in the reference situation.
211-213 I acknowledge that the authors correctly cite Chen et al. (2023) that increased sunshine hours and temperatures reduced the performance of maize-soybean intercropping. I find this a puzzling result, and without further evidence on the underlying mechanisms, I would be hesitant to accept this as a fact …
216 I had to read three times “shorter growth period overlap […] could contribute in reducing competition” before I understood it. It feels like a four-fold negation.
239-243 Personally, I would expect more opportunity for relay intercropping and double cropping with higher temperatures though there might be some areas with production decreases due to drought under climate warming. Just a thought.
262 competition (singular)
Fig. 4 Was there a need to add the notes that the partial land equivalent ratio of the other species was fixed? It does not matter for the result shown, does it?

(Remarks on code availability)

Reviewer #2

(Remarks to the Author)
The authors used machine learning models to estimate the potential contribution of intercropping soybeans with maize to improve Europe’s self-sufficiency. The rationale sounds. The paper is well-written and easy to follow. The implications of the results could influence policy and research prioritization.

However, there are too many assumptions, and the results are highly uncertain. Most importantly, the authors do not have the appropriate data to train their models on actual data from intercropping systems. Their approach to model each crop separately and then apply weights (0.56 for soybean and 0.79 for maize) to estimate intercropping yields was probably the best they could do, but, I am very skeptical of the results because they do not account for the interactions that will occur in reality by intercropping maize with soybean. Furthermore, the meta-analysis study the author cite does not reflect Europe's

production environments.

Results from this study, which are very promising (13 Mha of intercropping could cover 50% and 75% of the EU's consumption in soybean and maize) would create expectations that may influence policy not in the right direction. While the authors did the best they could do, including sensitivity analysis to explore the impact of their assumed values, I believe yield predictions of intercropping systems at scale is a very premature task, especially in Europe where farmers have no prior experiences with soybean cultivation.

(Remarks on code availability)
na

Reviewer #3

(Remarks to the Author)
The manuscript entitled "High potential contribution of intercropping to soybean and maize self-sufficiency in Europe" evaluates the cropland requirements needed to achieve 50% and 75% self-sufficiency in soybean and maize production across the EU. The authors demonstrate that intercropping could reduce land demand by 27–30% compared to sole cropping systems. To estimate yields, they develop a Random Forest-based machine learning model trained using GDHY (yield), SPAM2010 (irrigation), and ERA5 (weather) datasets, and apply a yield-threshold-based spatial allocation approach to identify suitable growing regions. Cropland is allocated using a soybean-first, yield-ranked assignment strategy, and intercropping performance is parameterized using partial Land Equivalent Ratio (pLER) values reported in Xu et al. (2020, Field Crops Research).
While the modeling framework is technically sound and clearly described, the main findings are somewhat expected. Given the adopted pLERs of 0.56 for soybean and 0.79 for maize (total land saving ≈ 0.56 + 0.79 – 1 ≈ 0.3), the reported 27–30% land saving aligns with theoretical expectations. However, as acknowledged by the authors in Line 202, intercropping efficiency is highly variable (ranging from 0.5 to 2.0) depending on local environmental conditions and cropping system configurations. This variability introduces substantial uncertainty that is not fully explored. Moreover, the study does not address practical constraints of intercropping—such as increased harvesting difficulty, crop protection, and irrigation complexity—which could limit real-world adoption and should be discussed.
Regarding the GDHY dataset used to train the yield model, there appears to be a significant limitation for soybean. According to Supplementary Figure 1, very few data points from Europe were used in model training for soybean, which may affect prediction accuracy. This important limitation should be explicitly acknowledged in the discussion section.

Specific Comments:
• Line 52–53:
Wu et al. (2023) does not report a doubling of production, but rather a 67.4 million ton increase on top of the current 19 million tons, which is closer to a fourfold increase. Please correct this statement.
• Line 187–189:
"Our study is the first providing evidence on the benefits of intercropping…"
This claim appears overstated. The study does not present new experimental evidence but rather extends the findings from Xu et al. (2020) to a European context through modeling. Please consider rephrasing this claim to reflect that the analysis is based on extrapolated meta-analytical data.
Line 204:Please clarify how intercropping is expected to be beneficial in areas where the sum of soybean and maize pLER values is less than 0.5. Such values would indicate very low productivity under intercropping.
Lines 318–319:The reference to "25%, 50%, 75%, or 100% of the average annual soybean consumption" is inconsistent with the scenarios described in the abstract, which only refer to 50% and 75% self-sufficiency. Please ensure consistency throughout the manuscript.
Line 322:Please clarify the rationale for selecting 25 Mha (25% of EU cropland) as the maximum allocation limit to avoid competition with other crops. Is this threshold based on existing land-use statistics, policy guidelines, or modeling assumptions? It would also be helpful to indicate what percentage of EU cropland is currently allocated to maize and soybean combined, for context.
Line 491 / Figure 1:Why does the yield ratio map (Figure 1c) show values in the Nordic countries where the individual maize and soybean yield maps (Figures 1a and 1b) contain no data? Additionally, what explains the hotspot around Switzerland in Figure 1c, where maize yields are high while soybean yields are extremely low?
Figures 2 & 3:Please ensure that the legend color schemes—particularly the yellow class—are used consistently between Figures 2 and 3. Any inconsistency in classification or color meaning should be clarified.

(Remarks on code availability)
I was unable to access the code at the provided link. When I attempted to review it on May 10, 2025, the repository appeared to have been removed or was otherwise inaccessible. I also examined an alternative repository (https://github.com/MathildeChen/SOYBEAN_PRED_COMP), which contains the code but includes minimal documentation and no accompanying data.

Version 1:

Reviewer comments:

Reviewer #1

(Remarks to the Author)
Review of Chen et al.: High potential contribution of intercropping to soybean and maize self-sufficiency in Europe

(for Word version with better layout, see attachment)

I have commented on the original version of this manuscript and expressed as my opinion that I agree with the authors on the high potential contribution of intercropping to soybean and maize self-sufficiency in Europe, but I felt the analysis was not strong enough and did not adequately address uncertainties in the estimation of intercrop performance. The original analysis was based on machine learning models to estimate the soybean and maize sole crop yields across Europe, while values of soybean pLER and maize pLER from a meta-analysis by Xu et al. (2020) were used to extrapolate these crop yields to production in intercrops. I found that analysis too simple in addressing the uncertainties in intercrop productivity as compared to sole crops, given the huge variation in intercrop performance compared to sole crops reported in the literature. The authors have responded satisfactorily to this criticism in their response letter. Based on the response letter, I understand that in the revised paper, different pLER values for maize and soybean are used under the assumption of simultaneous vs relay intercropping and under the assumption of "average N input, as in the meta-analysis" as compared to a European estimate of N input, based on the 75 percentile of N input to maize in Europe from Eurostat. This sounds good.

My comments are now mainly on the way these things are written up in the manuscript, which I found lacking in clarity. I suppose that with rigorous editing, the linkage between results, concepts and methods can be improved. Three key things did not become clear to me: (1) is soybean allocated to the cropland that gives the highest sole soybean yields in Europe according to the ML model, by first ranking all sites according to the soybean yield and then selecting that number of sites that would result in sufficient production (area times yield) to reach a certain production target, e.g. 25 or 50% self sufficiency), or is soybean allocated evenly to all the land that is climatically suitable for soybean cultivation? (Actually, it is stated in the M&M at the end of the paper, but readers need to understand it earlier, when they read the results) (2) the land that is allocated to soybean was previously allocated to another crop; it did not become clear to me which crop was replaced. I think this needs clearer description and possibly discussion regarding the consequences of substitution. (3) The authors used regressions from Xu et al. (2020) on the effects of N input and simultaneous vs relay intercropping to assess the relative performance of intercrops compared to sole crops under European growing conditions, and then explored whether these relationships affected their main thesis: maize-soybean intercropping has high potential for increasing self-sufficiency for soybean in Europe. It was not found to be sensitive. If wonder whether it would be possible to illustrate this lack of sensitivity in the main body of the paper graphically.

Detailed comments
Please note that detailed comments were written "as I went". It means that some queries might be answered later in the text, e.g. regarding methods used to produce the results. I chose not to edit these comments because the paper needs to be written in such a way that the readers knows enough about methods that such questions would not arise.

16 "and by minimizing crop substitution" At this point in the paper, it is not clear what is meant. When reading further, I am not sure we did get information on the degree of substitution. Maybe this is implied somehow, but it is not explicit.

16-18 "Maize-soybean intercropping, even limited to 25% of croplands in the EU, could meet 25%, 50%, and 75% of soybean demand with 6.3, 13.1, and 20.1 Mha, respectively". This is confusing as written. Why is there a constraint of "25% of cropland in the EU" in this sentence? Suggest to rephrase for clarity. E.g. Cultivation of maize-soybean intercrops on 6.3, 13.1, and 20.1 Mha of European cropland could meet 25, 50 and 75% of soybean demand, respectively. Maybe I misunderstand …

19 Which threshold is meant? You mean "Greater self-sufficiency than 75% would require that more than 25% of European cropland was used for maize-soybean intercropping"? I am not sure whether you mean that in any one year, more than 25% of the EU crop land would be under maize-soybean intercropping (MSI), or you mean that more than 25% of EU crop land would be cultivated with MSI in one out of four years, i.e. more than 6.25% under MSI in any one year. This aspect needs to be clarified.

19-21 This sentence is unclear: "When comparing […] than in 0.5 ha of sole soybean". Please rephrase.

29 I think that instead of "became" you might say here "has become" (as of now) or add the year by when this became so.

43-44 I suppose this information that soybean consumes more water than maize comes straight from the cited paper that two of the authors co-authored, but I am surprised. Based on my knowledge and experience, I thought that the water use by maize is usually a little greater than that of soybean. This opinion was backed up when I used chatgpt to find information. Please check.

[Text Redacted]

So, in summary: yes, published literature generally supports maize using somewhat more water (or at least achieving higher yields per water) under many conditions, but it is not a universal rule and depends on context.

64-71 The text in these lines seems to suggest that the LER sets a maximum to the intercropping advantage that may be attained in reality. However, the LER that we have from experiments is an average across different conditions that might include those that the authors mention here, including cases where one of the sole crop does not or hardly perform; hence I wonder whether the conclusion would still hold if the LER is not a fixed mathematical property but the outcome of variable sole crop and intercrop performance across growing conditions and ecoregions.

77 quantified -> quantify

87 “rainfed yields”. Suggest to add: “of soybean and maize” for clarity.

96-111 This section is not so clear. I understand that you use a minimum soybean yield of 1 t/ha but no minimum for maize. Why no minimum for maize?

99 “and to the 25th percentile of our projections under rainfed conditions” What is meant? Do you use the 25 percentile of the rainfed soybean yield projections to say that all the areas with greater yield are suitable? Why is the 25 percentile used and why do you not use the same approach and same percentile for maize? Rationale missing. Then the median of the remaining 75% of yield projections of soybean is 2.1 t/ha? It needs to be written up in a less ambiguous way. It is key to write up in a consistent way how you define where soybean and maize may be grown in the future. On top, readers may want to know what crops are replaced. It may not be the case, as suggested in the abstract, that crop substitution is minimized. This would need to be shown?

100 The -> A

102 largely -> greatly

101-102 Production area of 82 million ha has median yield of 2.1 t/ha but current area is only 1 million ha. What does this tell us about the acceptability of a soybean yield of 2.1 t/ha and the associated gross margin? What do growers cultivate currently on the 81 million ha that they could use for soybean but use for another crop? Do they grow a more profitable crop? What other crop? If it is maize, there is really no problem, but if it is wheat, alfalfa, or grass, maybe there is a problem. Some contextualization is needed to make sense of the reported numbers.

119 meet -> achieve

121-123 How do you achieve that intercropping is primarily implemented in areas with high soybean yields? Do you do this by making a selection of suitable regions with high enough yields and distribute evenly, or do you do this by ranking pixels according to soybean yield and allocating pixels to soybean or MSI cultivation until the production target is met? This needs to be made clearer. (Actually, it is in M&M, but the reader needs to know ahead of time.)

122 excluded from -> excluding

124 “we assume” Suggest to add: “as a baseline”

129 Add “arable” to “cropland”?

130 Please include rationale why the cultivation of soybean and maize should be simultaneous, so readers know your reasoning.

132-135 Self sufficiency for soybean in attained more easily if the crop is grown in areas in which it performs well. Is this done in all of the scenarios that follow? (See also above in line

121-123 and general comment no. 1).

142-143 Are the areas with higher soybean production prioritized in the allocation?

156 “stronger competition” here means simultaneous intercropping. I would advocate using the term “simultaneous intercropping” to be clear and upfront. You may explain elsewhere that relay intercropping boost production by temporal complementarity, i.e. competition avoidance.

157 a N fertilization -> an N fertilization (an because N is pronounced as “en”, so it starts with a vowel if pronounced. (also in 188)

171 conventional -> conventionally managed. I suggest a change in wording because MSI is by no means “conventional”. It is an innovative system, but it can still be conventionally managed, i.e. with artificial fertilizer and biocides.

183 Suggest to write “assumed” before “LER values”.

184-186 Please harmonize tense (mix of present and past tense)

185-186 I’d suggest omitting “regardless of intercropping’s frequency in crop sequences”. In any case, it is a bit implicit whether the reported area of soybean, maize and MSI are in each year, or they are areas in which these crops are cultivated, e.g., in 1:4 years.

203-204 I don’t understand why pLERs of 0.30 and 0.31, respectively, are needed to achieve 25 and 50% soybean self-sufficiency. The difference in pLER is quite small, but the difference in self-sufficiency is large. I suppose that the self-sufficiency is mostly driven by the cultivation area of soybean or MSI. But why then are the minimum pLERs so similar?

Please clarify.

208 Why does maize self-sufficiency not exceed 75% when soybean pLER exceeds 0.41? I would think that maize production and maize self-sufficiency are altogether independent from soybean pLER. I am missing something in the reasoning. If there is insufficient maize production in MSI, then the remainder of maize can be produced in sole crops? This is related to what is replaced by MSI? Is it maize? Is it another crop?

215 A pLER of 0.46 for a legume is not low. See, e.g., Yu et al. (2015, 2016) and Li et al. (2020).

Li CJ, Hoffland E, Kuyper Th W, Yu Y, Zhang CC, Li HG, Zhang FS, van der Werf W (2020) Syndromes of production impact yield gains in intercropping. Nature Plants 6, 653–660. http://dx.doi.org/10.1038/s41477-020-0680-9
Yu Y, Stomph TJ, Makowski D, van der Werf W (2015) Temporal niche differentiation increases the land equivalent ratio of annual intercrops: a meta-analysis. Field Crops Research 184, 133–144. http://dx.doi.org/10.1016/j.fcr.2015.09.010
Yu Y, Stomph TJ, Makowski D, Zhang L, van der Werf W (2016) A meta-analysis of relative crop yields in cereal/legume mixtures suggests options for management. Field Crops Research 198: 269-279. http://dx.doi.org/10.1016/j.fcr.2016.08.001

216 "25 million hectares" Suggest to add: "of maize soybean intercropping"

238 Add here "growing the intercrops in areas that are the most suited for soybean cultivation across Europe"?

250-253 "If more productive intercropping scenarios […] in the least productive intercropping scenario". I suppose you are only talking about soybean pLER here. Then I would refrain from calling it "productivity". LER is not productivity. It is relative performance.

255 See previous comment: better call it simultaneous intercropping

266 condition -> conditions

275 Omit "only"

310-312 What is replaced?

338 producing -> production

338 How was detrending done? You mean time trend?

339 What is the meaning of a detrended "mean yield"? Conceptually, which year is represented by the detrended mean yield; e.g. the yield in 1980, in 2030? Is it a future year or a year in the past?

352 Are these predictions of soybean yield for water limited rainfed conditions or irrigated conditions?

353 for each crop -> for both crops (to leave no doubt that there are just two species involved)

376 This is fine; but I am still a bit confused whether 25 Mha of a crop at EU level means that EACH YEAR, there is 25 Mha of this crop, or there is Mha of land in which this crop occurs in a 1:4 rotation, so 6.25 Mha in each year. I presume the former, but I am not 100% it is not the latter. Please state it.

381-398 This section is fairly clear, but I had to read several times to be sure I understood. I think readers need the understanding conveyed by this paragraph when they read the results (which are BEFORE the materials and methods). I suppose that more methods need to be infused into the results to make readers (and this reviewer) understand the methods when reading the results.

381-398 Why does the area of MSI need to be capped at 25 Mha? Is this because this represents 25% of the whole EU arable crop area, and soybean cannot be cultivated at greater frequency than 1:4? Please, if so, state it.

381-398 I wonder whether adopting MSI would lower the maize cultivation frequency on parcels in maize growing areas. If so, this could boost maize yields by mitigating soil-borne diseases. Furthermore, incorporation of soybean in rotations benefits productivity of cereals (e.g. Xia et al., 2023, but there are many other sources)

Xia HY, Qiao YT, Li XJ, Xue YH, Wang N, Yan W, Xue YF, Cui ZL, van der Werf W (2023) Moderation of nitrogen input and integration of legumes via intercropping enable sustainable intensification of wheat-maize double cropping in the North China Plain: A four-year rotation study. Agricultural Systems 204: 103540. https://doi.org/10.1016/j.agsy.2022.103540

388-389 You mention the area of wheat but do not state what the area is. Is it 25 Mha? Please clarify. Is it necessary to consider the replacement of wheat area by MSI area, with consequences for wheat self-sufficiency? Does it need quantification or discussion?

416 Please make clear this is about relay intercropping.

518 Incomplete reference.

Figure 1 Colour scale of panels a and b is counter-intuitive, with dark blue (cold) representing the highest yields and yellow (somewhat warm) representing the lowest yield. Please consider using a different colour scale that is more intuitive. In panel c it is unclear whether the ratio shown is maize/soybean yield or soybean/maize yield. Sentence in lines 593-596 does not run.

Figure 2 This figure did not help me as a reader. It might help if the caption would explain the procedure and all symbols used, but in the current situation, the reader needs to figure out the meaning by himself. Suggest to move to supplementary or make it totally self explanatory.

Figure 3 Please enlarge the maps. They tell an important story about the allocation but they are too small.

(Remarks on code availability)

Reviewer #3

(Remarks to the Author)
I thank the authors for their efforts in revising the manuscript. Many aspects have been improved. However, my main concern regarding the static ratio LER = 0.56 + 0.79 remains unresolved.

I acknowledge the authors' argument that yield variability of the two crops and the allocation model may introduce some variation. However, the allocation model itself is relatively simple and based on many assumptions, which may not be sufficient to justify the conclusions. More importantly, the central focus of the manuscript is intercropping. The efficiency or land-saving benefit of intercropping is measured by the land equivalent ratio (LER):

LER = f(weather, crop allocation, management)

where management includes nitrogen fertilization, irrigation regime, and relay vs. simultaneous planting. In other words, LER is not a fixed value but a dynamic outcome of these interacting factors. While the ML yield model developed by the authors successfully captures weather effects, it does not explicitly address LER, which is the primary driver of land-use efficiency and land saving.

In addition, the authors applied a fixed nitrogen rate of 148 kg N ha^{-1} to calculate LER using the regression equation from Xu et al. This approach neglects spatial variability in nitrogen management and, therefore, does not provide a solid basis for conclusions about land saving.

To strengthen the manuscript, I recommend that the authors consider constructing a machine learning model directly for LER and investigate how interactions among weather, management, and allocation factors could be extrapolated from other regions to Europe. Such an approach would provide more robust and generalizable evidence of the land-saving potential of intercropping under diverse conditions.

Another major concern is the allocation of intercropping, sole soybean, and sole maize. Based on the current description of the methods, it is difficult to reconcile the results presented in Figure 1 with those in Figure 3. Greater methodological clarity is needed.

Specific comments:
• Figure 2: A clear legend or explanation for P^i_m and P^m_m is still missing.
• Figure 3:
1. Why does southeastern France show no intercropping or soybean cultivation, despite Figure 1 indicating higher yields than in northwestern France?
2. Inconsistencies remain between the intercropping and land-saved maps.

(Remarks on code availability)
The code associated with this manuscript has not yet been made publicly available. In the response to reviewers, the authors provided a different link, which does not directly correspond to the present manuscript.

Version 2:

Reviewer comments:

Reviewer #1

(Remarks to the Author)
Review of Chen et al. High potential contribution of intercropping to soybean and maize self-sufficiency in Europe

This article has potential, and I agree with the main thesis that maize/soybean intercropping has great potential for boosting self sufficiency for soybean and maize, but the problems in presenting results in a clear manner persist in this version. I had

difficulty in correctly interpreting the abstract and results, though I had little trouble with the introduction, discussion and methods sections which are clear. I feel readers need to be told upfront that total land area in the EU is about 100 Mha, of which 1 Mha is soybean and 9 Mha is maize. Readers need to know upfront whether yields are seed yields or biomass yields (maize may be harvested as both), and if this is not relevant, that needs to be stated and it needs to be explained why it does not matter. Proposed planting areas of intercrops are huge (up to 25 Mha; 25% of the EU arable land area) and it is not clear whether this area increase is from expansion of the arable land area, from partially reducing maize sole cropping (which would make sense) or from replacing another crop (e.g. wheat). Maybe the authors consider this a side issue, but without telling readers, readers cannot well contextualize results. I thus feel further modifications are needed.
Authors might include a table detailing soybean, maize and intercrop areas for different scenarios along with the production of soybean and maize, and include this in the results section. Doing so they may remove many numbers from the narrative and focus the narrative more on the logic and ideas. This could help to make the results section more accessible.

ABSTRACT
I wrestled with the abstract. I found it left out critical information on the area and production of maize which made it difficult to assess the findings. Specifically:
14-17 “Increasing domestic production of one of these species could reduce the production of the other. We examine the potential for maize-soybean intercropping to improve the European Union's soybean self-sufficiency while minimizing competition with maize and other crops.” Maybe rephrase as “while minimizing trade-offs with THE PRODUCTION OF maize and other crops”.
17-20 “Assuming a one-in-four years frequency, 25%, 50%, and 75% self-sufficiency would be achieved with 6.3, 13.1, and 20.1 Mha of maize-soybean intercropping, resulting in total co-production of 37.9, 81.9, and 126.2 Mt of both crops, respectively.” This is confusing as written as the previous sentence was emphasizing self-sufficiency for soybean, so this sentence would then be about 25, 50 and 75 self-sufficiency for SOYBEAN, but then it goes on to mention the TOTAL grain yields of the two species together. Is this what is meant? To avoid ambiguity, this might be rephrased as “Assuming a one-in-four years frequency, 25%, 50%, and 75% self-sufficiency FOR SOYBEAN would be achieved with 6.3, 13.1, and 20.1 Mha of maize-soybean intercropping, resulting in total co-production of 37.9, 81.9, and 126.2 Mt of both crops, respectively.” Still this does not inform the reader what this means for the sufficiency of maize and other crops production and the area of land used for intercropping + maize in the scenarios compared to the area of land used for soybean + maize under current conditions.
21 intercropping -> intercrops
17-23 I feel lines 17-23 do not give readers a clear picture of the soybean and maize production under the depicted scenarios of 25, 50 and 75% soybean self sufficiency, and the corresponding soybean and maize areas required to reach the same production of the two crops. I realize that this might require more space, but then perhaps the message needs to be simplified.
21-24 “To achieve comparable levels of co-production, separate cultivation of the two species would require an additional 36.5, 24.7, and 11.1 Mha of land, corresponding to an estimated land saving of 20–21% under intercropping.” Readers cannot judge the relevance of increases in land area of 36.5, 24.7 and 11.1 Mha of land as the reference areas have not been provided. The percentage land saving makes sense, but likely follows directly from the LER of maize/soybean intercropping. Then the greater conclusion is that the LER obtained from field-level studies extrapolates well to continent level land use efficiency, even if geographic variation in the water limited potential yields of maize and soybean is taken into account.
24-25 A pLER > 0.5 may be hard to achieve for soybean in an intercrop with maize if both species are grown simultaneously as maize is highly competitive in such intercrops. This is an important caveat.

INTRODUCTION
35-36 To set the stage clearly, it would be useful to mention here both the soybean and maize areas in the EU and the average yields and production obtained.
Generally the introduction is clear and sets the stage well. The workflow in the last paragraph is clear.

RESULTS
99-102 “The 25th, 50th (i.e., median), and 75th percentiles of averages soybean yield projections for 2000-2023 across the EU are 1.0, 2.1, and 2.6 t.ha-1, respectively (Figure 1a, top panel). These levels of productivity are reached over 103, 82, and 36 Mha, respectively (Figure 1a, bottom panel), where wheat, maize, and barley are the major cultivated crops (Supplementary Table 2).” This sentence maybe misunderstood easily. I first though the percentiles were for the average yield estimate across the whole EU (a single number for the whole continuent). And then it made no sense that corresponding areas were mentioned. Upon rereading several times, I gathered that you estimated yields for each grid cell and obtained a distribution across the whole EU for which the percentiles are given. Please rephrase to remove the ambiguity in the term “average yield”.
105-107 For context you might mention here the total arable land area in the EU, so readers can immediately see that the 103 Mha is more than 25% of the total arable land area in the EU, actually, even more than the WHOLE arable land area in the EU.
142 “maize’s productivity remains above zero” What do you mean? Of course maize’s productivity is above zero. Do you mean the change in production? You want to minimize trade-off?
149-152 “This system would produce 18.2 Mt of soybean and 63.7 Mt of maize (Figure 3c), representing nearly a sevenfold increase in current EU soybean production while maintaining maize production at nearly the same level (Supplementary Table 1).” For the reader it is not clear whether intercropping in this scenario is replacing sole maize or something else.
144-155 To be more comprehensive in the presentation of results, it might be beneficial to present for each scenario soybean area, maize area, intercrop area, and the associated soybean and maize production. Somewhat in line with Supplementary Table S3. This would greatly facilitate the insightfulness of the results. It would also clarify to which extent

maize or other crop species are replaced. Such a table would have to be in the main text, not in supplementary. When the table is included, the text can be simplified because readers can see the numbers in the table.
156-171 The narrative in this paragraph would be supported by including yet another scenario in the 50% self sufficiency for soybean is achieved by increasing soybean area (at the expense of maize?) and again reporting soybean and maize cropping areas as well as the soybean and maize production. The maps are nice, but they are not a good vehicle to support the text on area requirements to reach certain levels of sufficiency. In line with previous comment: a table is needed.
189-193 This is a best case; be cautious.
211 A soybean pLER of 0.53 with TND = 0 seems optimistic. It maybe in line with the results of Xu et al. (2019) but I am not sure. Maybe those data would need to be analysed specifically for pLERs of both soybean and maize with both higher N input and low TND (also because of mechanization difficulties if TND>0). Are the data of Xu et al. (2019) available from public sources or from the authors?
224-226 The truth of this statement depends on whether intercropping replaces maize or another crop. If intercropping replaces maize, then less intercropping would mean greater self sufficiency for maize production in the EU; however, if intercropping replaces another crop than maize, less intercropping indeed would mean lower self sufficiency for maize production in the EU but if would imply higher self sufficiency for the unnamed crop that is replaced by intercropping. It depends on what crop is replaced by intercropping; but that is not clear. I feel a table with clear scenario assumptions (as suggested above) could help in this respect.
241-243 “Interestingly, 50% soybean self-sufficiency and 100% maize self-sufficiency would be simultaneously achieved a maize pLER of 0.79 as soon as soybean pLER remains lower than 0.41.” This result is not logical. If the pLER of soybean decreases, while that of maize stays the same, a greater area of intercropping is needed to match a given production target of soybean. Then the self sufficiency of maize is bound to change, getting lower if intercropping replaces sole maize and getting higher if intercropping replaces another crop than maize. Maybe you mean AT LEAST 100% maize self-sufficiency. Please rephrase for clarity.

DISCUSSION
311 Sorting of grain is indeed needed if the crops are harvested as bulk. In China, where maize/soybean intercropping is practiced and advocated by the national government, the crops are harvested separately, using specific equipment. See Li Chunjie et al. (2020) in Nature Plants. Li Long and colleagues at China Agricultural University investigate wide strip maize/soybean intercropping to facilitate separate harvesting with bigger than traditional equipment as shown in Li et al. (2020). In colder areas (e.g. Tibet) encouraging results have been obtained with using maize/soybean silage (Yang Wenyu, personal communication). Li CJ, Hoffland E, Kuyper Th W, Yu Y, Zhang CC, Li HG, Zhang FS, van der Werf W (2020) Syndromes of production impact yield gains in intercropping. Nature Plants 6, 653–660. http://dx.doi.org/10.1038/s41477-020-0680-9
351-353 “Our findings suggest that to achieve 91% soybean self-sufficiency, more than 25 Mha of maize-soybean intercropping would be required, corresponding to an increase of 15 Mha compared to the current harvested areas of maize and soybean in the EU.11” From this I would calculate the current maize area in the EU is 25 – 1 – 15 = 9 Mha. But if that is true, the planting of 15 Mha of intercropping would result in a big overproduction of maize in the EU. This would also suggest further adaptations to the implemented scenarios to avoid such overproduction?

METHODS
384-392 Please clarify whether yields are seed yields (standard moisture percentage) or seed yield dry matter, or biomass (fresh or after drying).
432-440 Please clarify which land is allocated to intercropping. Total arable land area in the EU currently is about 100 Mha, of which 1 Mha is soybean and 9 Mha is maize. Scenarios for increased self sufficiency for soybean result in a major area allocation to soybean/maize intercropping, up to 25 Mha for reaching 91% soybean self sufficiency. The text in 432-440 mentions that arable land area increase is avoided, and competition with maize growing area is also avoided. Somehow, it is not clear where the land is coming from, and it may even be questioned if the land area is available.

• What are the noteworthy results?
Maize/soybean intercropping, if applied at a large scale in Europe, has potential major benefit for self sufficiency for both maize and soybean in the EU. This is not a surprising result, but this paper is the first to come up with somewhat credible quantification.
• Will the work be of significance to the field and related fields? How does it compare to the established literature? If the work is not original, please provide relevant references.
The work is important as a hallmark in quantification of the benefits of intercropping in Europe if applied at a large scale. It may make policy makers aware of the need to invest in research and technological development to make intercropping of maize and soybean technically achievable and known and acceptable to farmers in the EU.
• Does the work support the conclusions and claims, or is additional evidence needed?
Yes, the conclusions are supported. I agree with the conclusions and the analysis, but found the presentation needs improvement to enhance its clarity and transparency (see above).
• Are there any flaws in the data analysis, interpretation and conclusions? Do these prohibit publication or require revision?
I don’t think there are important flaws. There are uncertainties, but these are acknowledged.
• Is the methodology sound? Does the work meet the expected standards in your field?
I am satisfied.
• Is there enough detail provided in the methods for the work to be reproduced?
Yes.

(Remarks on code availability)

Reviewer #3

(Remarks to the Author)
Again, I thank the authors for their careful revision. The manuscript has improved in clarity, structure, and transparency. In particular, the description of the allocation procedure is now clearer (e.g., Figure 2 and corresponding text), and additional sensitivity analyses have been conducted to explore the robustness of the results to variations in pLER values and key drivers such as nitrogen input and temporal niche differentiation.

However, my main concern remains only partially addressed.

The central focus of the manuscript is the efficiency of intercropping, which is quantified through the land equivalent ratio (LER). As noted previously, LER is not a fixed parameter but a dynamic outcome of interacting factors including climate, soil conditions, and management practices (e.g., fertilization, irrigation, and relay versus simultaneous intercropping). While the authors acknowledge this and perform sensitivity analyses across a wide range of pLER values, LER remains treated as an exogenous parameter rather than being explicitly linked to the same drivers that determine crop yields in the model.

In the current framework, crop yields are spatially explicit and climate-driven (via the machine learning model), whereas intercropping performance is imposed through externally derived pLER values that do not vary consistently with environmental conditions. This creates a structural inconsistency: the model captures spatial variability in sole crop productivity but not in the intercropping advantage itself. As a result, the estimated land-use efficiency and land-saving potential depend critically on assumptions about LER that are not mechanistically or statistically integrated into the modeling framework.

The additional sensitivity analyses are useful and demonstrate that the main conclusions are numerically robust across a range of assumed pLER values. However, they do not fully resolve the underlying issue that LER may co-vary with climate and management conditions across Europe. Therefore, the results should be interpreted as conditional on the assumed distributions of pLER, rather than as fully spatially resolved estimates of intercropping performance.

I encourage the authors to further clarify this point in the manuscript. In particular:
- The limitations section should more explicitly state that LER is not modeled as a function of environmental drivers and that this may affect spatial allocation results.
- The framing of the conclusions could be adjusted to emphasize that the reported potential of intercropping is conditional on assumed pLER values.
- If feasible, even a simplified stratification of pLER values across agro-climatic zones would strengthen the consistency of the framework.

Overall, the manuscript addresses an important question and has been substantially improved. Nevertheless, the treatment of LER remains a key limitation that should be more explicitly acknowledged and discussed to ensure that the conclusions are interpreted appropriately.

(Remarks on code availability)
The codebase reflects solid scientific programming and implements the paper’s core methodology effectively, particularly the spatial allocation framework for maize–soybean intercropping, the random forest setup, the PCA-based climate preprocessing, and the baseline use of partial land equivalent ratios for soybean and maize. At the same time, its present form limits reproducibility and broader community use: several scripts rely on system-specific file paths, the large raw input datasets are not included in the repository even though their sources are documented, some sensitivity-analysis scripts described in the paper are missing, and the README would be stronger with clearer installation guidance, dependency specifications, data access instructions, and troubleshooting notes. The documentation does provide a useful overview of the workflow and data provenance, but reproducing the full analysis still requires substantial setup and adaptation. Even so, the cached outputs appear sufficient to regenerate the final figures with limited effort, and the main workflow should be reproducible with file-path revisions, appropriate data organization, and a better-defined software environment. Overall, this is a well-constructed and scientifically valuable codebase whose reproducibility and community uptake would be improved by platform-independent paths, more explicit setup documentation, and packaging tools such as dependency locking or containerization.

Version 3:

Reviewer comments:

Reviewer #1

(Remarks to the Author)
Authors provide a convincing analysis that use of maize/soybean intercropping will contribute substantially to soybean and maize self sufficiency in Europe if applied at a large scale. Results extend the meta-analysis results of Xu et al. (2021) and provide a compelling analysis of potentials of this intercropping system in the EU. The results are important as they progress from existing LER values, which are abstractions, to economic potentials, which will probably trigger more the imagination of policy makers and other stakeholders. Hence, this study could have high impact. The machine learning values for yield projections help give credibility to the results. Evidence is sufficient, and there is ample analysis of uncertainties. I have not

seen any flaws. Methods are sound and are at the level the journal should expect. There is a lot of information in supplementary, and I understand that codes are provided (but I did not check those). There are still some minor issues in the text that might be clarified. I do provide an annotated word file with minor suggestions for improving clarity and language, at the discretion of the authors.

I have seen previous versions of this ms, and my concerns are sufficiently addressed in this version.

(Remarks on code availability)

Reviewer #3

(Remarks to the Author)
I thank the authors for their careful revision. The manuscript has improved substantially in clarity and transparency. In particular, the allocation procedure is now much clearer, and the additional sensitivity analyses for pLER values, nitrogen input, and temporal niche differentiation help readers understand the conditional robustness of the conclusions.

My main concern regarding the treatment of LER as an exogenous parameter rather than as a modeled function of environmental and management drivers has now been sufficiently acknowledged as a limitation. The revised Discussion appropriately states that spatial variability in pLER could not be modeled due to the lack of suitable experimental datasets, and the conclusions are now more clearly framed as conditional on assumed pLER values.

I therefore do not see a remaining critical issue that would prevent publication. I only recommend minor editorial corrections before acceptance:

- Harmonize the description of allocation ranking, which is described as soybean production in the Results but projected soybean yield in the Methods.
- Report land-saving values consistently as positive percentages, or clarify the sign convention in Table 1.
- Check minor rounding inconsistencies, such as 18.1 vs 18.2 Mt.

Overall, I am satisfied with the revision and support publication after these minor editorial corrections.

(Remarks on code availability)

Open Access This Peer Review File is licensed under a Creative Commons Attribution 4.0 International License, which permits use, sharing, adaptation, distribution and reproduction in any medium or format, as long as you give appropriate credit to the original author(s) and the source, provide a link to the Creative Commons license, and indicate if changes were made.
In cases where reviewers are anonymous, credit should be given to 'Anonymous Referee' and the source.
The images or other third party material in this Peer Review File are included in the article’s Creative Commons license,

unless indicated otherwise in a credit line to the material. If material is not included in the article's Creative Commons license and your intended use is not permitted by statutory regulation or exceeds the permitted use, you will need to obtain permission directly from the copyright holder.
To view a copy of this license, visit https://creativecommons.org/licenses/by/4.0/

RE: Manuscript reference: NCOMMS-25-06543-T entitled "High potential contribution of intercropping to soybean and maize self-sufficiency in Europe"

Mathilde CHEN [a,b,c,*], Nicolas GUILPART [d], David MAKOWSKI [a,*]

[a] Université Paris-Saclay, INRAE, AgroParisTech, UMR MIA PS, 91120 Palaiseau, France
[b] CIRAD, UMR PHIM, F-34398 Montpellier, France.
[c] PHIM, Univ Montpellier, CIRAD, INRAE, Institut Agro, IRD, 34398 Montpellier, France
[d] Université Paris-Saclay, AgroParisTech, INRAE, UMR Agronomie, 91120 Palaiseau, France

Emails: mathilde.chen@cirad.fr, nicolas.guilpart@agroparistech.fr, david.makowski@inrae.fr

RESPONSE TO REVIEWERS' COMMENT

REVIEWER #1

Remarks to the Author:

1.1. I have read this paper with anticipation and interest because I share the authors' belief that intercropping maize and soybean is an overlooked opportunity for increasing production of maize and soybean in Europe while moderating inputs. The topic is important, and I feel the basis conclusions of the paper are cautious enough to be right.

Our response: Thank you for this positive feedback.

1.2. Nevertheless the analysis left me unsatisfied and unconvinced. The prime reason for this is that the results depend critically not so much on the machine learning that the authors have done (highly competent, as far as I can judge), but on the values for the land equivalent ratio (LER) that were obtained in a previous study (Xu et al., 2020). The machine learning (ML) focuses on the yields of soybean and maize and was mostly used to select regions where soybean yields are high enough (>1 t/ha) to grow soybean as sole crop or as an intercrop with maize. Then the ML models are used to predict yields in the sole crops, and the average LER from Xu et al. (2020) is used to obtain the intercrop yield.

It is no surprise that the results of the study suggest that large improvements in self-sufficiency in soybean production can be achieved by growing maize-soybean intercrops on a large area. This conclusions follows straight from the assumed LER which was obtained from Xu et al. (2020).

However, whether this LER will materialize in Europe is highly uncertain due to lack of experiments. The effect of climatic conditions and management on the LER are likely to be important. The work by Xu et al. (2020) but also by Yu et al. (2015) and by Li et al. (2020) showed the large importance of temporal complementarity between the crop species, i.e. relay intercropping has greater production advantages than simultaneous intercropping, particularly at higher input levels (Li et al., 2020). I would have expected for an analysis of this kind that an attempt would have been made to assess whether temporal complementarity would be obtainable given the heat sums available across Europe.

Moreover, meta-analyses have shown the high importance of N input for LER (e.g. Yu et al., 2016, Liu et al., 2024). Generally, in cereal-legume intercropping, the LER decreases with N input as the mechanism for complementarity (different sources of N for the two crop species) is taken away. With no temporal complementarity and contemporary western-European N input into the crop system, I would not expect a land equivalent ratio for maize-soybean intercropping substantially above 1.10, though it could be higher under conditions of lower fertilization (but at low yield levels) and it could be substantially higher if substantial temporal complementarity can be achieved. I feel the study would be a lot more convincing if somehow these effects of N input and temporal complementarity would have been incorporated to generate projections.

I am not saying the results are wrong. I agree with the findings, I just feel that the most important issue determining the prospects of maize-soybean intercropping is not sufficiently addressed and leaves the reader with large uncertainty. Currently uncertainty is addressed with a sensitivity analysis, which could be fine in itself, but I feel it is not satisfactory as the LER is the factor of overriding importance influencing the yield advantages that can be attained and that are the core of the paper. The LER depends on the growing conditions and management. The ML models are interesting but they only address the yields in sole cropping and do not address the extent to which intercropping can achieve complementarity and overyielding.

Li CJ, Hoffland E, Kuyper Th W, Yu Y, Zhang CC, Li HG, Zhang FS, van der Werf W (2020) Syndromes of production impact yield gains in intercropping. Nature Plants 6, 653–660. http://dx.doi.org/10.1038/s41477-020-0680-9
Liu YL, Stomph TJ, Zhang FS, Li CJ, van der Werf W (2024) Nitrogen input strategies impact fertilizer nitrogen saving by intercropping: A global meta-analysis. Field Crops Research 318: 109607. https://doi.org/10.1016/j.fcr.2024.109607
Xu Z, Li CJ, Zhang CC, Yang Y, van der Werf W, Zhang FS (2020) Intercropping maize and soybean increases efficiency of land and fertilizer nitrogen use; A meta-analysis. Field Crops Research 246: 107661. http://dx.doi.org/10.1016/j.fcr.2019.107661
Yu Y, Stomph TJ, Makowski D, van der Werf W (2015) Temporal niche differentiation increases the land equivalent ratio of annual intercrops: a meta-analysis. Field Crops Research 184, 133–144. http://dx.doi.org/10.1016/j.fcr.2015.09.010
Yu Y, Stomph TJ, Makowski D, Zhang L, van der Werf W (2016) A meta-analysis of relative crop yields in cereal/legume mixtures suggests options for management. Field Crops Research 198: 269-279. http://dx.doi.org/10.1016/j.fcr.2016.08.001

Our response: We thank the reviewer for this clear and complete comment. Following the suggestions made by the reviewer, we performed a new sensitivity analysis to take into account the effects of high N inputs and temporal differentiation niche (TND) on values of partial land equivalent ratios (pLERs) of soybean and maize.

Specifically, we used the regression models published by Xu et al. (2020) to compute less optimistic pLER values (i.e. lower) assuming high N fertilizer doses and low TND, and assess the robustness of our conclusions considering these lower pLER values. First, we assumed a fertilization rate of 148 $kg.ha^{-1}$, which corresponded to the 75th percentile of N dose applied on maize in the European Union (EU) members states in 2017-2018, according to the data of the International Fertilization Association (Ludemann et al., 2022). This level of fertilization led to pLER values of 0.51 and 0.78 for soybean and maize, respectively, based on the results of Xu et al. (2020). Second, we assumed that both species had identical growing periods (i.e., TND=0), which led to pLER values of 0.53 and 0.76, respectively, based on the results of Xu et al. (2020).

We computed the level of European self-sufficiency with these new pLER values (see Supplementary Figure 10 in the revised manuscript, also displayed below, and new Supplementary Tables 3 and 4) and showed that our conclusion was only marginally changed, even in a high N inputs scenario and in case of strong competition between both species.

Reference:

Ludemann, C.I., Gruere, A., Heffer, P. et al. Global data on fertilizer use by crop and by country. Sci Data 9, 501 doi:https://doi.org/10.1038/s41597-022-01592-z (2022).

Xu, Z. et al. Intercropping maize and soybean increases efficiency of land and fertilizer nitrogen use; A meta-analysis. Field Crops Research 246, 107661, doi:https://doi.org/10.1016/j.fcr.2019.107661 (2020).

The following texts were added in the revised paper:

Methods, lines 416-425:

"To evaluate the effects of high N inputs and temporal niche differentiation (TND) on values of partial land equivalent ratios (pLERs) of soybean and maize, we used the regression models published by Xu et al. (2020) to compute less optimistic pLER values (i.e., lower) assuming high N fertilizer doses and low TND, and assess the robustness of our conclusions considering these lower pLER values. First, we assumed a fertilization rate of 148 kg.ha^{-1}, which corresponded to the 75th percentile of N dose applied on maize in the European Union (EU) members states in 2017-2018, according to the data of the International Fertilization Association (Ludeman et al., 2022). This level of fertilization led to pLER values of 0.51 and 0.78 for soybean and maize, respectively. Second, we assumed that both species had identical growing periods (i.e., TND=0), which led to pLER values of 0.53 and 0.76, respectively. Further details on these sensitivity analyses are provided in Supplementary Material 5."

Results, lines 155-164:

"Land saving allowed by intercropping slightly decreased in simulations considering high N inputs and stronger competition between intercropped species, two factors which were found to impact maize-soybean intercropping efficiency (Xu et al., 2020). Assuming a N fertilization rate of 148 kg.ha-1 (i.e., 75th of N rate applied on maize in the EU in 2017-2018; Ludemann et al., 2022) and corresponding to pLERs values of 0.51 and 0.78 for soybean and maize (Xu et al., 2022) respectively, intercropping uses 24% and 22% less land compared to sole cropping to achieve similar co-production of both crops using targeting 25% and 50% soybean self-sufficiency in the EU (Supplementary Table 3). Similarly, 20% and 23% self-sufficiency for soybean and maize, respectively, could be achieved in a scenario characterized by a TND of zero (i.e., both species are sown and harvested at the same time) and corresponding pLERs values of 0.53 and 0.76 (Xu et al., 2022), respectively (Supplementary Table 4)."

Results, lines 186-193:

"Similar levels of self-sufficiency were achieved after incorporating the effects of N inputs and temporal niche differentiation (TND) on the pLERs (Supplementary Figure 10). Assuming a N fertilization rate of 148 kg.ha^{-1} and pLERs values of 0.51 and 0.78 for soybean and maize, respectively, the potential self-sufficiency rates ranged from 47 to 86% for soybean and 83 to 143% for maize, respectively, depending on the return frequency. Similarly, 49-90% and 80-138% self-sufficiency for soybean and maize, respectively, could be achieved in a scenario characterized by a TND of zero (i.e., corresponding pLERs values: 0.53 and 0.76), respectively."

Supplementary Table 1. Soybean and maize coproduction and surface requirement for 25, 50, 75, and 100% soybean self-sufficiency in the European Union (EU) achieved by intercropping or sole cropping *assuming a nitrogen fertilization rate of 148 kg/ha*.

Target % of EU's self-sufficiency in soybean [a]	Intercropping		Sole crops			Total		Land-saving [b]
	Soybean	Maize	Soybean	Maize	Additional land required	Intercrop-ping	Sole crop	
**Production (Mt)**								
**25%**	9.1	32.1	9.1	22.2	10.1	41.2	41,3	
**50%**	18.2	70.8	18.2	48.8	22.0	89.0	89,0	
**75%**	27.2	110.2	27.3	74.5	21.1	137.4	122,8	
**100%**	29.9*	122.5	36.3	65.0	5.3	152.4	106,7	
**Surface (Mha)**								
**25%**	7.0		3.4	3.6	1.5	7.0	8.5	-22%
**50%**	14.7		7.1	7.6	3.5	14.7	18.2	-24%
**75%**	22.6		10.9	11.7	3.3	22.6	25.9	*-15%***
**100%**	25.0		14.8	10.2	0.9	25.0	25.9	*-3%***

Notes: Crop return frequency was one-in-four year for all simulations, and total allocated area was limited to 25% of croplands in the EU (i.e., 25 Mha). The nitrogen fertilization rate of 148 kg/ha is the 75th percentile of nitrogen fertilization rate of maize in 2018 in the EU (Ludeman et al., 2022). The values of partial land equivalent ratios of intercropping corresponding to this level of nitrogen fertilization were 0.50 and 0.78 for soybean and maize, respectively, based on the results of Xu et al. (2020).

[a] Self-sufficiency levels of 50, 75, and 100% correspond to 18.2, 27.2, 36.3 Mt soybean.

[b] Computed as the percentage of surface saved by intercropping compared to sole crop strategy.

* Target production not reached even when soybean was grown of 25% cropland in the EU.

** Production from intercropping strategy higher than sole crops.

Supplementary Table 2. Soybean and maize coproduction and surface requirement for 25, 50, 75, and 100% soybean self-sufficiency in the European Union (EU) achieved by intercropping or sole cropping _assuming that maize and soybean are grown during the same period**_.**

Target % of EU's self-sufficiency in soybean [a]	Intercropping		Sole crops			Total		Land-saving [b]
	Soybean	Maize	Soybean	Maize	Additional land required	Intercrop-ping	Sole crop	
**Production (Mt)**								
**25%**	9,1	29,6	9,1	20,4	9,3	38,7	38,8	
**50%**	18,2	65,5	18,2	45,1	20,4	83,6	83,7	
**75%**	27,2	101,7	27,3	68,5	27,0	128,9	122,8	
**100%**	31,1*	118,1	36,3	65,0	5,3	149,2	106,7	
**Surface (Mha)**								
**25%**	6,8		3,4	3,4	1,4	6,8	8,1	-20%
**50%**	14,1		7,1	7,0	3,2	14,1	17,3	-23%
**75%**	21,6		10,9	10,7	4,2	21,6	25,9	*-20%***
**100%**	25,0		14,8	10,2	0,9	25,0	25,9	*-3%***

Notes: Crop return frequency was one-in-four year for all simulations, and total allocated area was limited to 25% of croplands in the EU (i.e., 25 Mha). Growing both soybean and maize during the same time period is translated by a temporal differentiation niche of 0. The values of partial land equivalent ratios of intercropping corresponding to this temporal differentiation niche were 0.53 and 0.76 for soybean and maize, respectively, based on the results of Xu et al. (2020).

[a] Self-sufficiency levels of 50, 75, and 100% correspond to 18.2, 27.2, 36.3 Mt soybean.

[b] Computed as the percentage of surface saved by intercropping compared to sole crop strategy.

* Target production not reached even when soybean was grown of 25% cropland in the EU.

** Production from intercropping strategy higher than sole crops.

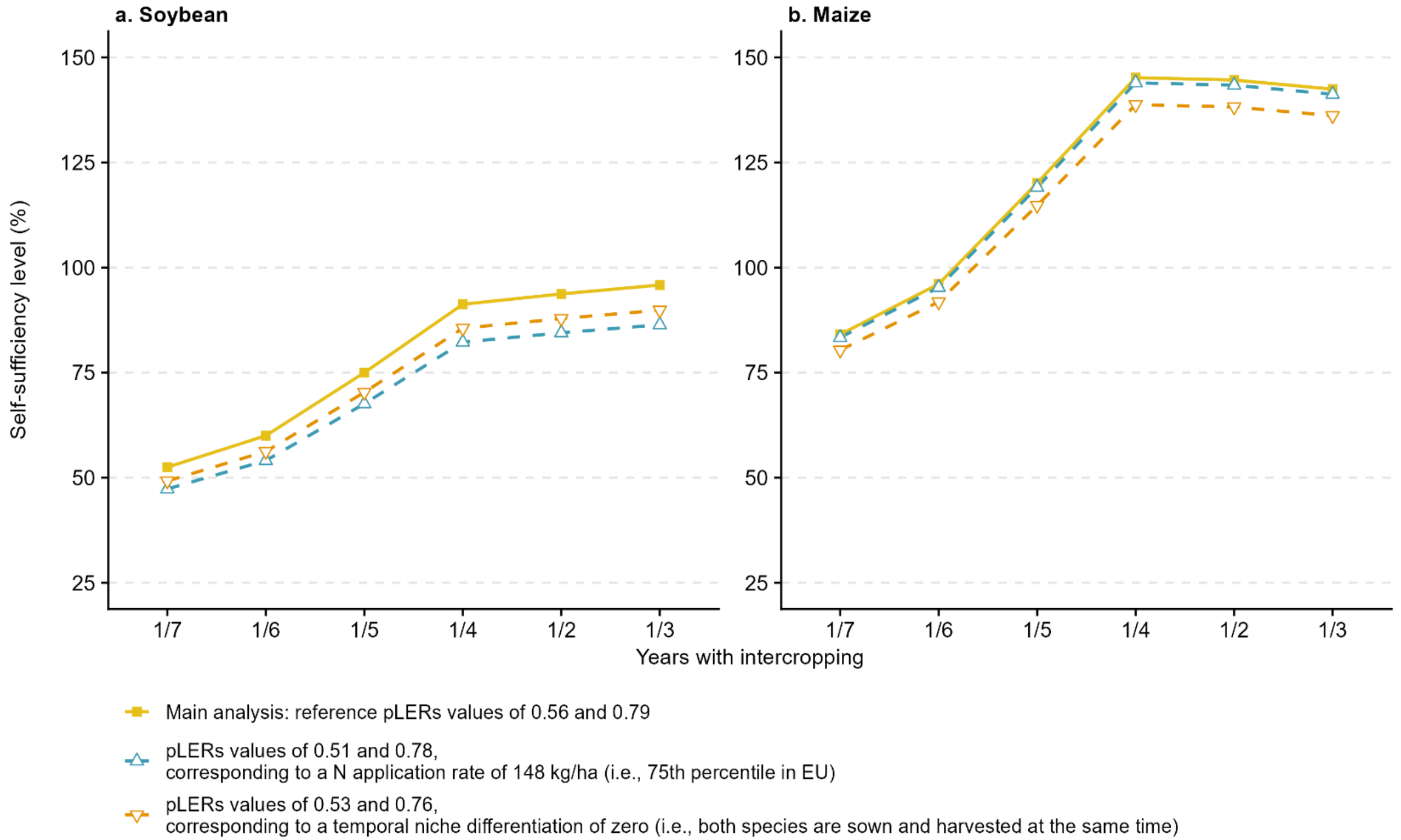

Supplementary Figure 10. Levels of soybean (a) and maize (b) self-sufficiency in the European Union (EU) achieved from intercropping for different assumptions of partial land equivalent ratios (pLERs) and crop return frequency. Crop frequencies of one year in seven, six, five, four, three, or two correspond to allocating maize-soybean intercropping on 14%, 16%, 20%, 25%, 33%, or 50% of cropland area in each grid-cell. Intercropping was first allocated to soybean highest-yielding grid-cells, and in all cases total intercropping area was constrained to not exceed 25% of croplands in the EU (i.e., 25 Mha). Squares represent the results obtained with pLER values of 0.56 for soybean and 0.79 for maize, corresponding to the average productive performances of maize-soybean intercropping systems estimated in a previous meta-analysis (Xu et al., 2020). Triangles represent the results obtained with pLER values of 0.50 for soybean and 0.78 maize, corresponding to maize-soybean intercropping systems with a nitrogen fertilization rate of 148 kg/ha (i.e., equivalent to the 75th percentile of nitrogen fertilization rate of maize in 2018 in the EU [Ludeman et al., 2022]), based on the results of Xu et al. (2020). Circles represent the results obtained with pLER values of 0.53 for soybean and 0.76 for maize, corresponding to a temporal differentiation niche of 0 (i.e., both soybean and maize are grown during the same time period), based on the results of Xu et al. [2020]).

Detailed comments

1.3. 18-21 This statement in the abstract is highly contingent on the value of LER used. The estimate from Xu et al. (2020) is high. It does reflect the literature, but new experiments are needed to assess the current yield advantages. In experiments I have been involved in in China under current conditions, the LER was about 1.10 (not responsive to N input because of high N stock in the soil). LERs from relay intercropping are likely (substantially) higher, but the paper provides no evidence that relay intercropping would be achievable in Europe given the current climatic conditions.

Our response: Thank you for these comments regarding intercropping's performance in the European Union under current agronomic and climatic conditions.

Regarding the effect of agronomic practices on LER, especially N inputs, see our response to comment #1.2. above about the robustness of our conclusions in a scenario of higher N fertilization and in the revised manuscript (Supplementary Figure 10).

Following the comment of the reviewer, and subsequent comments from other reviewers (i.e., #2.3), we examined the climate conditions covered by the experimental data used in the meta-analysis of Xu et al. (2020) to estimate maize and soybean pLERs, in order to see how representative they are of the area where we allocate intercropping in the European Union.

We computed the mean temperature and precipitations during soybean growing period in each experiment site included in the meta-analysis Xu et al. (2020) and in the European grid cells considered in our yield projections over the 2000-2023 period. Cropping calendar were derived from USDA website[1] and climate data were taken from the ERA5-land dataset, also used in our study to model crop yield (Supplementary Material 2). Additionally, we compared the distribution of experimental and projection sites based on their Köppen-Geiger zones (Kottek et al., 2006).

Both analyses show that climate conditions of experimental sites included in the meta-analysis, especially those in Europe, both in North and South Americas, as well as several in Asia and one in Australia, are representative of the sites where intercropping is projected in our study (Supplementary Figures 21-22, also displayed on the next pages). Based on the Köppen-Geiger classification, most of experimental and projection sites are located in temperate areas (42.9% and 60.3%, respectively; Supplementary Figure 22). These results suggest that in current climatic conditions, intercropping might probably be achievable in the European Union, but we acknowledge that more studies are required to confirm this.

It is worth noting that the LER estimated from experimental data exclusively collected in Europe reaches 1.48 ± 0.087, suggesting a higher productivity than the level estimated at a global scale (LER = 1.32 ± 0.02) (Xu et al., 2020). Our estimations based on the "global" LER, which is slightly lower compared to the "European" LER, are then likely to be conservative.

[1]USDA Crop Calendar Charts available at: https://ipad.fas.usda.gov/ogamaps/cropcalendar.aspx

These elements are now included as Supplementary Figures 21 and 22 in the revised manuscript.

References:

Kottek M, J. Grieser, C. Beck, B. Rudolf, and F. Rubel (2006) World Map of the Köppen-Geiger climate classification updated. . Meteorol. Z. 15:259-263.

Muñoz-Sabater, J. et al. ERA5-Land: a state-of-the-art global reanalysis dataset for land applications. Earth Syst. Sci. Data 13, 4349-4383, doi:10.5194/essd-13-4349-2021 (2021).

Xu, Z. et al. Intercropping maize and soybean increases efficiency of land and fertilizer nitrogen use; A meta-analysis. Field Crops Research 246, 107661, doi:https://doi.org/10.1016/j.fcr.2019.107661 (2020).

Methods, lines 365-371:

“The reference pLER values used in this study – 0.56 for soybean and 0.79 for maize – come from a meta-analysis of soybean-maize intercropping trials (Xu et al., 2020). Results of this meta-analysis were estimated using 654 field observations collected in 108 sites worldwide, with environmental conditions partially representative to those in the EU (Supplementary Figures 21-22). Although this meta-analysis also provides an estimation based on data collected in Europe specifically, we choose to use the global estimate, which is less optimistic (i.e., lower pLER), preventing us to overestimate the potential contribution of intercropping.”

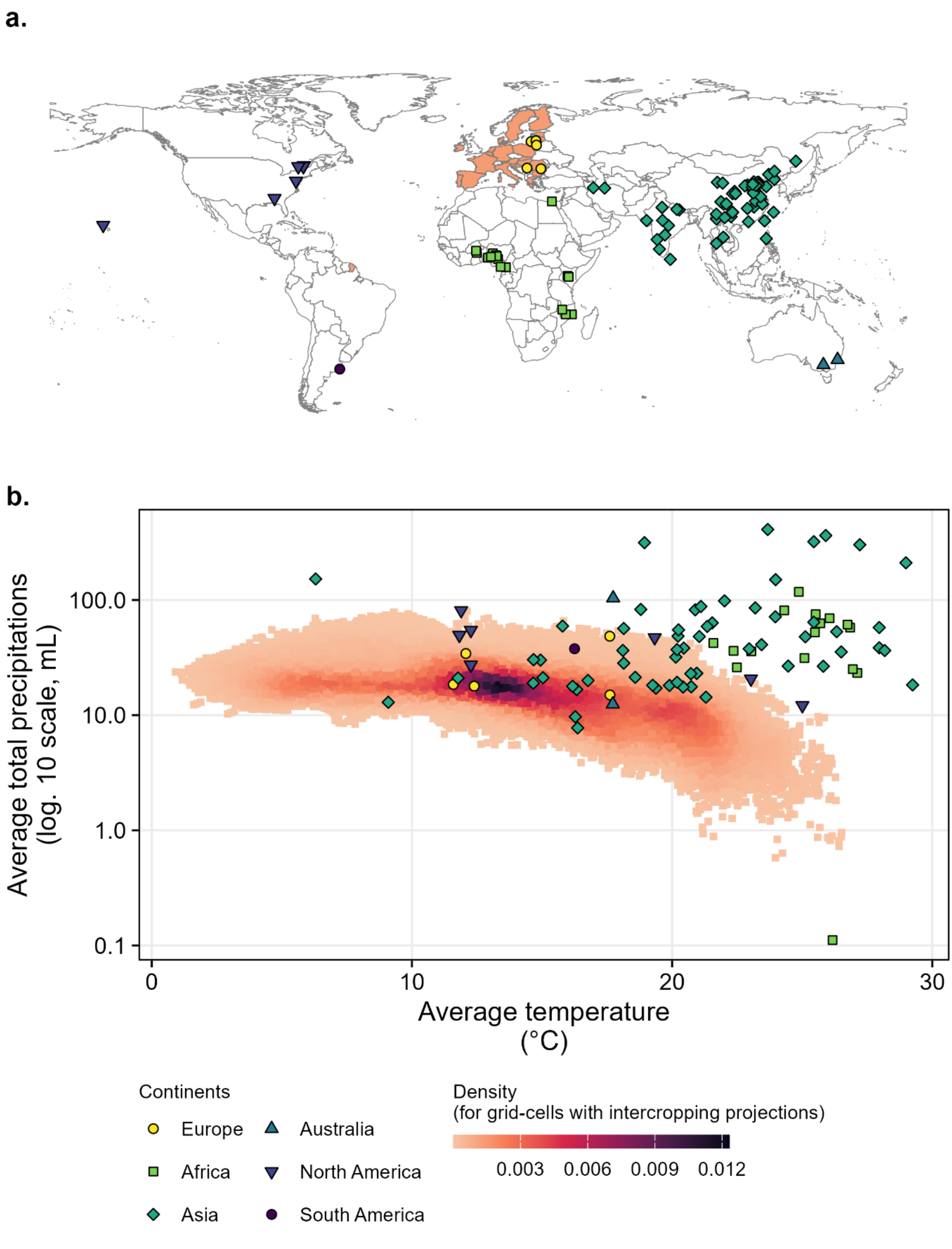

Supplementary Figure 21. Location (a), average temperature and total precipitations during soybean growing period over 2000-2023 (b) in the experimental sites used in the meta-analysis of Xu et al. (2020) and in the sites where soybean yields are projected in the present study. Cropping calendars for each site was determined using the USDA's crop calendars charts. For each site, monthly temperatures and total precipitations were derived from the ERA5 land dataset and then averaged over soybean growing period.

a.

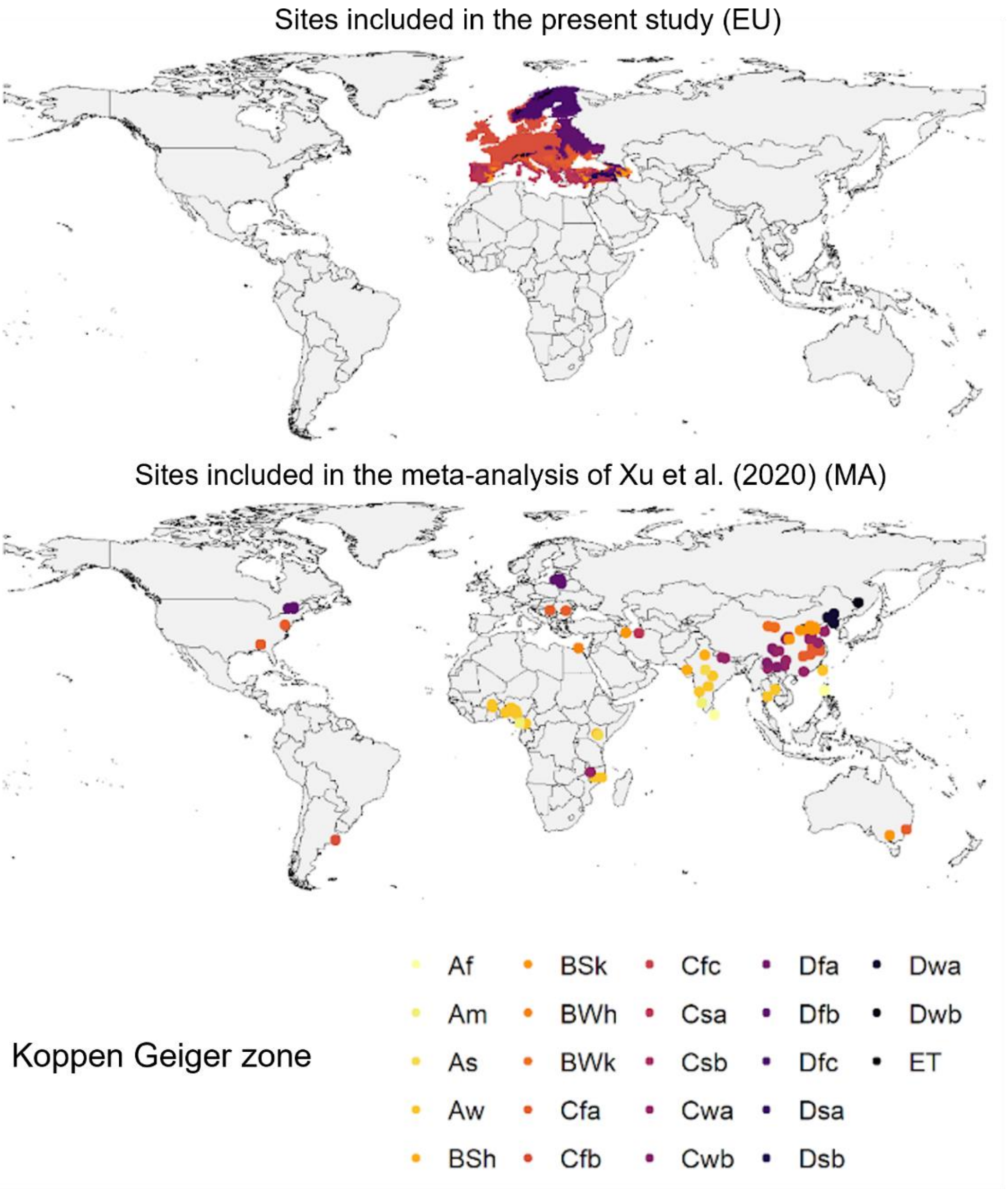

b.

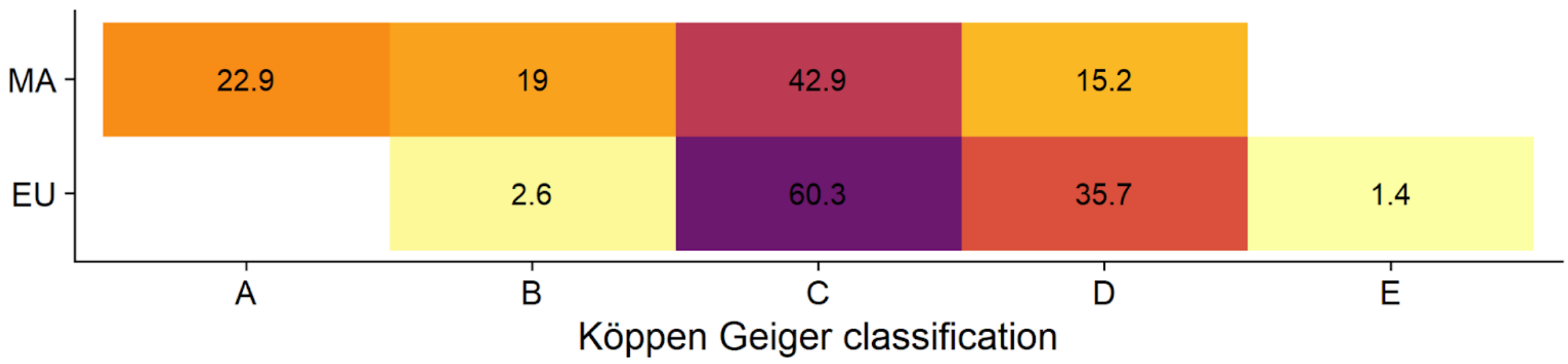

Supplementary Figure 22. Location (a) and distribution (b) of Köppen-Geiger zones among the sites included in the meta-analysis of Xu et al. (2020) [MA] and in the sites where soybean yields are projected in the present study [EU]. The numbers in (b) are the percentages of sites of the meta-analysis (MA) and in the projected European yield map (EU) located in each Köppen-Geiger zone.

1.4. 52-54 The paper cites a paper by Wu et al. (2023) stating that “In China, intercropping maize with soybean was found to double soybean production”. I initially misread this statement thinking you meant the yield per ha was doubled and therefore checked the source. Please clarify to avoid such misreading.

Our response: Thank you for your comment. Wu et al. (2023) report that intercropping maize with soybean on 50% of the maize planting area in China in 2018 could more than double the total soybean production in this country. This latter result is now provided in the revised version of the manuscript.

Introduction, lines 52-53:

“In China, intercropping maize with soybean on 50% of the maize planting area could more than double the total soybean production in 2018 in this country (Wu et al., 2023).”

Reference: Wu, Y. et al. Soybean yield variations and the potential of intercropping to increase production in China. Field Crops Research 291, 108771, doi:https://doi.org/10.1016/j.fcr.2022.108771 (2023).

1.5. 62-70 The authors bring up an interesting point in differences in sole crop yields across Europe. However, I don’t see clearly how this would affect the land saving or production increases from intercropping because these depend on the complementarity (the LER), not on the monocrop yields. In other words, I have the impression that we would obtain very similar results if we would just apply the LER to the European average yields of maize and soybean on the area that is judged suitable for growing this combination, without caring about the regional differences in absolute yield. Am I missing something?

Our response: The results would not be same because, as some areas are more productive than others, the allocation of the crop species in the most productive areas may make sole cropping systems more productive than expected according to the LER. Although the spatial variability might be relatively low at the field scale, this is an important aspect to consider at large scales. This is illustrated below in a numerical example, and a general proof is given in Supplementary Material 1.

In the present study, we consider a decision problem combining two distinct objectives, namely: 1) reach a target production level of soybean in a given region, and 2) maximizing the production of corn in the same region and in addition to soybean. We want to compare different cropping strategies, namely intercropping or sole cropping, in simultaneously satisfying both objectives. LER provides a clear answer to this problem only if the yields are the same everywhere in the region considered.

This is illustrated below in a numerical example where we consider:

- an area where the average yields of crop 1 and crop 2 in sole cropping are equal to Y1 = 4 $t.ha^{-1}$ and Y2 = 8 $t.ha^{-1}$, respectively,
- values of pLER of the two crops are equal to pLER_1=0.5 and pLER_2=0.6, respectively, corresponding to a LER of 1.1. Then, the average yields of crop 1 and 2 in intercropping are equal to 4*0.5 $t.ha^{-1}$ and 8*0.6 $t.ha^{-1}$, respectively.
- a target production level for crop 1 in this area is set equal to P*_1 = 400 t.

With intercropping, the requested area (Ai) for producing P*_1 is equal to:

$$Ai = P^*\_1 / (Y1 * pLER\_1) = 400 / (4 * 0.5) = 200 \text{ ha}$$

and the associated production of crop 2 in the intercrop (Pi_2) on the same 200ha is equal to:

$$Pi\_2 = Y2 * pLER\_2 * Ai = 8 * 0.6 * 200 = \mathbf{960\ t}$$

What would be the production achieved with monocropping in this area 200ha? The answer depends on the spatial variability of the yields in this area of 200ha. For example: we now assume that the yield of crop 2 is homogeneous in this total area of 200 ha but that the yield of crop 1 is heterogeneous in the same area. Specifically, in the most productive half part of the whole 200ha, we assume that the average yield of crop 1 is 50% higher than the average yield in the whole 200ha. If we allocate crop 1 to this most productive part, the yield of monocrop 1 is thus equal to 4*1.5. The requested area to reach the target production level P*_1 in the sole cropping system (As_1) is thus equal to:

$$As\_1 = 400 / (4 * 1.5) = 66.67 \text{ ha}$$

Here, we only need 66.67 ha of monocrop 1 to reach the target production level of crop 1. The remaining area (As_2) is available for monocrop 2:

$$As\_2 = 200 - As\_1 = 200 - 66.67 \text{ ha}$$

and the production of crop 2 in sole cropping (Ps_2) is then equal to

$$Ps\_2 = 8 *(200 - 66.67) = \mathbf{1066.64} \text{ t, and thus } \mathbf{Ps\_2 > Pi\_2}.$$

In this case, for maximizing total production, the best option is the monocropping system.

However, if the yields are homogeneous, As_1 = 400 / 4 = 100 ha, As_2=100ha, Ps_2=8*100=**800** t, and thus **Ps_2 < Pi_2**, i.e., intercropping is the best choice as indicated by the LER value.

More generally, the standard criterion LER provides the solution of this decision problem only when the yields of both crops are homogeneous over space, which is not usually the case in large scale studies (Figure 1). Then, LER is not sufficient to choose between the two strategies, even if LER is constant over the intercropping area. We give general proof of this result in Supplementary Material 1. We were able to define another criterion - based on a modified expression of LER - and, based on this new criterion, we see that a LER>1 is not sufficient to ensure that a strategy is better than another (Supplementary Material 1). The

introduction now refers to this appendix in order to clarify the utility to take into account the spatial variability of yields to compare intercropping and sole cropping strategies.

1.6. 96 Would soybean yields of 1.0 t/ha be judged acceptable in a European context? It seems low, but it may be ok.

Our response: As mentioned in the manuscript, this threshold represents the minimum soybean productivity in Italy between 1981 and 2016 in the historical yield dataset (Iizumi and Sakai, 2020). In addition, it corresponds to the 25th percentile of our yield projections in the European Union. This is now mentioned in the revised manuscript.

Reference: Iizumi, T. & Sakai, T. The global dataset of historical yields for major crops 1981-2016. *Sci Data* 7, 97, doi:10.1038/s41597-020-0433-7 (2020).

Results, lines 97-100:

"Considering a threshold of 1.0 t.ha^{-1} (i.e., minimum observed yield in major soybean-producing regions of our training dataset with climate conditions similar to those in the EU and to the 25th percentile of our projections under rainfed conditions), our projections suggest that 103 Mha could be considered as suitable for soybean production in the EU."

1.7. 103 equal to or higher than

Our response: Thank you spotting the typo. This is now corrected.

1.8. 119-128 This section is a bit hard to follow. I think you mean that the intercrop would be grown on the same area as soybean is currently grown. But perhaps you mean that the intercrop is grown in areas where predicted soybean yield is at least 1.0 t/ha. Please clarify.

Our response: We realize that it may be not totally clear for the reader that we perform two distinct allocation scenarios. First, we estimate the area requirements under maize-soybean intercropping to meet 25%, 50%, 75%, and 100% soybean self-sufficiency in the EU, assuming that intercropping would primarily be implemented in regions with the highest soybean productivity and excluded from those where soybean yield is lower than 1 t.ha^{-1}. Then, we estimate the area requirements to reach the same soybean self-sufficiency levels under a sole cropping system.

We agree that this section was confusing for the reader. We provided more details on the allocation procedure to make the results of this analysis clearer. In addition, we rephrased the outputs for clarity and readability and we now explicitly refer to the Figure 2 and Supplementary Material 2 which both provide all details about our allocation procedure.

Results, lines 114-147:

“Following the procedure displayed in Figure 2 and detailed in the Supplementary Material 2, we examine the potential of maize-soybean intercropping to improve soybean self-sufficiency in the EU while maintaining maize production and we quantify the land savings allowed by intercropping at the scale of the EU. The allocation procedure is summarized in Figure 2 and detailed in the Supplementary Material 2.

First, we estimate the area requirements under maize-soybean intercropping to meet 25%, 50%, 75%, and 100% soybean self-sufficiency in the EU (i.e., 9.1, 18.2, 27.2, and 36.3 Mt, respectively), assuming that intercropping would primarily be implemented in regions with the highest soybean productivity and excluded from those with lowest suitability (i.e., soybean yield below 1 $t.ha^{-1}$). To represent the current average productive performance of maize-soybean intercropping systems, we assume that 1 ha of intercropping produces as much as 0.56 ha of soybean and 0.79 ha of maize grown separately (Xu et al., 2020), corresponding to pLER values of 0.56 and 0.79, respectively. We consider that intercropping is grown once every four years (Hartman, West, and Herman, 2011; Pannecoucque et al., 2018), which is equivalent to yearly allocating 25% of the cropland area in each site to intercropping. To limit competition with other crops, we consider that intercropping could not be implemented on more than 25 Mha, corresponding to a quarter of cropland areas in the EU (Eurostats, 2024). Additionally, since both soybean and maize are simultaneously cultivated in such system and that maize’s productivity remains above zero in the whole intercropping area (Figure 1), we also estimate the quantity of maize produced in this area. For example, producing enough soybean to reach 50% self-sufficiency would require to preferably implement intercropping in southeastern part of France, North of Italy, Hungary, and Romania (first map in Figure 3a), covering an area of 13.1 Mha (Figure 3b). This system would simultaneously produce 18.2 Mt of soybean and 63.7 Mt of maize (Figure 3c), representing nearly a sevenfold increase in current EU soybean production while maintaining maize production at nearly the same level (Supplementary Table 1). In other words, 50% of the EU’s soybean needs and nearly 75% of its maize demand could be met, thereby more than tripling the current self-sufficiency rate for soybean (16%), while slightly reducing the maize self-sufficiency rate from its current level (81%).

Then, we estimate the area requirements to reach the same soybean self-sufficiency levels but under a sole cropping system. We find that 50% self-sufficiency could be reached by cultivating soybean on 7.1 Mha, as shown in Figure 3. The second map of Figure 3a shows the area required by sole soybean, while the third map represents the residual area of 6.0 Mha (i.e., 13.1-7.1 Mha) between intercropping and sole cropping. Based on our maize yield projections, growing sole maize on this remaining area would produce 39.0 Mt of maize, i.e., 39% less than the 63.7 Mt produced under intercropping for the same total area of 13.1 Mha (Figure 3c)."

References:

Eurostats. Utilised agricultural area by categories [update: December 19, 2024] Accessed in January 2025 (2024)

Hartman, G. L., West, E. D. & Herman, T. K. Crops that feed the World 2. Soybean—worldwide production, use, and constraints caused by pathogens and pests. Food Security 3, 5-17, doi:10.1007/s12571-010-0108-x (2011).

Pannecoucque, J. et al. Screening for soybean varieties suited to Belgian growing conditions based on maturity, yield components and resistance to Sclerotinia sclerotiorum and Rhizoctonia solani anastomosis group 2-2IIIB. The Journal of Agricultural Science 156, 342-349, doi:10.1017/S0021859618000333 (2018).

Wu, Y. et al. Soybean yield variations and the potential of intercropping to increase production in China. Field Crops Research 291, 108771, doi:https://doi.org/10.1016/j.fcr.2022.108771 (2023).

1.9. 120-124 I probably misread the sentence, but how can you maintain maize yields if an intercrop maize-soybean is grown in the highest yielding soybean areas? Soybean areas have no maize yield at all. Please clarify. You must mean something else than I read.

Our response: We agree that this section was hard to follow and we tried to improve the readability. See our response to comment #1.8.

1.10. 132-139 These results feel a bit “deja-vu” for people who are familiar with the LER concept. They follow straight from the LER. But the LER is uncertain.

Our response: The reviewer can refer to our response to comment #1.5 and to new Supplementary Material 1.

1.11. 149-150 I feel some reservation about these results because they depend totally on the assumed LER value which may not be achievable across Europe. Studies in China have shown vast variation in LER, mainly driven by temporal complementarity (ongoing work by Tao et al., but see also Li et al. (2020) and Xu et al. (2020)).

Our response: Thank you for your comment. See our answer to comment #1.3 and new Supplementary Figures 11-14.

1.12. 158 provided

Our response: This is now corrected in the revised version of the manuscript.

1.13. 157-172 The sensitivity analysis is helpful, but it mixes somehow the effect of LER and of total area used for production of maize and soybean. I mean: in Fig. 4 we see that at the lowest pLER of soybean (0.3) and at the lowest frequency of maize-soybean in the rotation (1/7), the self-sufficiency for soybean is still increased from ~15 to >25%, which is a vast increase. But at this LER, there is also a production increase for maize while the total LER is only 1.09 (0.3 + 0.79). As a reader I do not get a good enough understanding of the areas used in the scenario and in the reference situation.

Our response: Thank you for your comment. To clarify this point, we performed new sensitivity analyses where we examined scenarios with varying partial land equivalent ratios (pLERs) for soybean and maize (from 0.3 to 0.7 and from 0.5 to 0.9, by 0.01-increment, respectively). The results of these sensitivity analyses are displayed in the following pages (Supplementary Figures 11 to 14).

These new results show that the higher soybean pLER, the lower surface required by intercropping to reach a given target self-sufficiency threshold. In other words, less land is required to produce a certain quantity of soybean in a scenario where intercropping shows higher soybean pLER. In this case, as intercropping area is lower, less maize is produced by the intercropping. On the opposite, when soybean pLER is lower, more maize is produced because a higher intercropping area is required to produce the same target quantity of soybean. When targeting higher soybean production objectives (i.e., 75% or 100% soybean self-sufficiency, Supplementary Figures 13-14), one limiting factor is the maximum area allocated to intercropping (here limited to 25 Mha).

The manuscript now describes these analyses (see Results section and new Supplementary Figures 11-14).

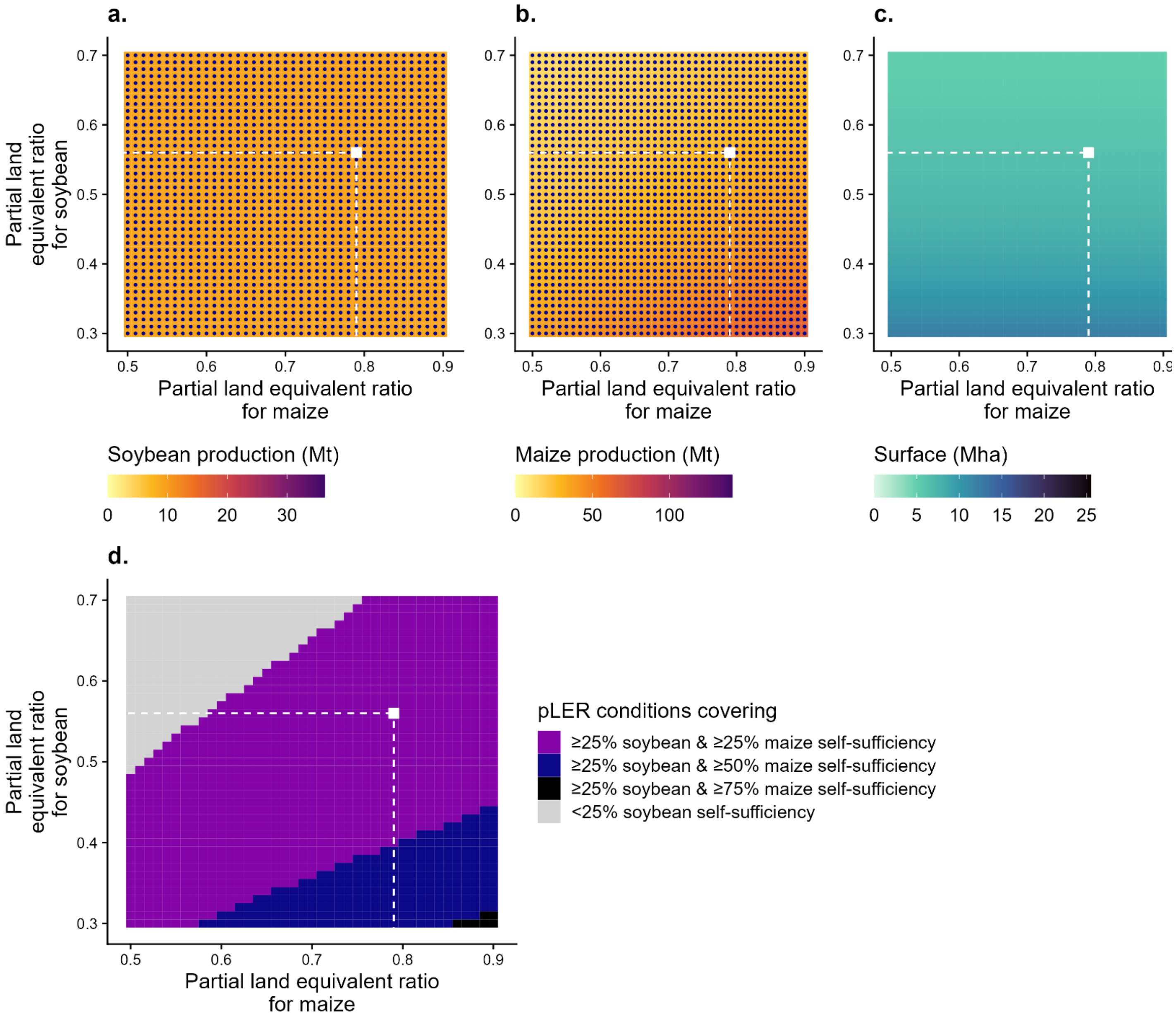

Supplementary Figure 11. Characteristics of maize-soybean intercropping allocations in terms of soybean produced (a), maize produced (b), and surface allocated (c) for covering 25% soybean self-sufficiency in the European Union depending on intercropping efficiency (pLER). The minimum efficiency conditions simultaneously satisfying 25% of soybean and 25, 50, 75, or 100% maize self-sufficiency are also shown (d).

Scenarios with soybean and maize productions satisfying 50% and 100% self-sufficiency (i.e., 9.0 and 85.1 Mt, respectively) are highlighted by blue dots in panels (a) and (b), respectively. All scenarios assume a one-in-four year return frequency of intercropping and the total surface is limited to 25% of cropland area in the European Union (i.e., 25 Mha). Scenarios where the surface allocated to intercropping reaches that threshold are represented by the red dots in panel (c). In all panel, the white dot represent the current estimation of maize-intercropping efficiency at a global scale estimated by Xu et al. (2020).

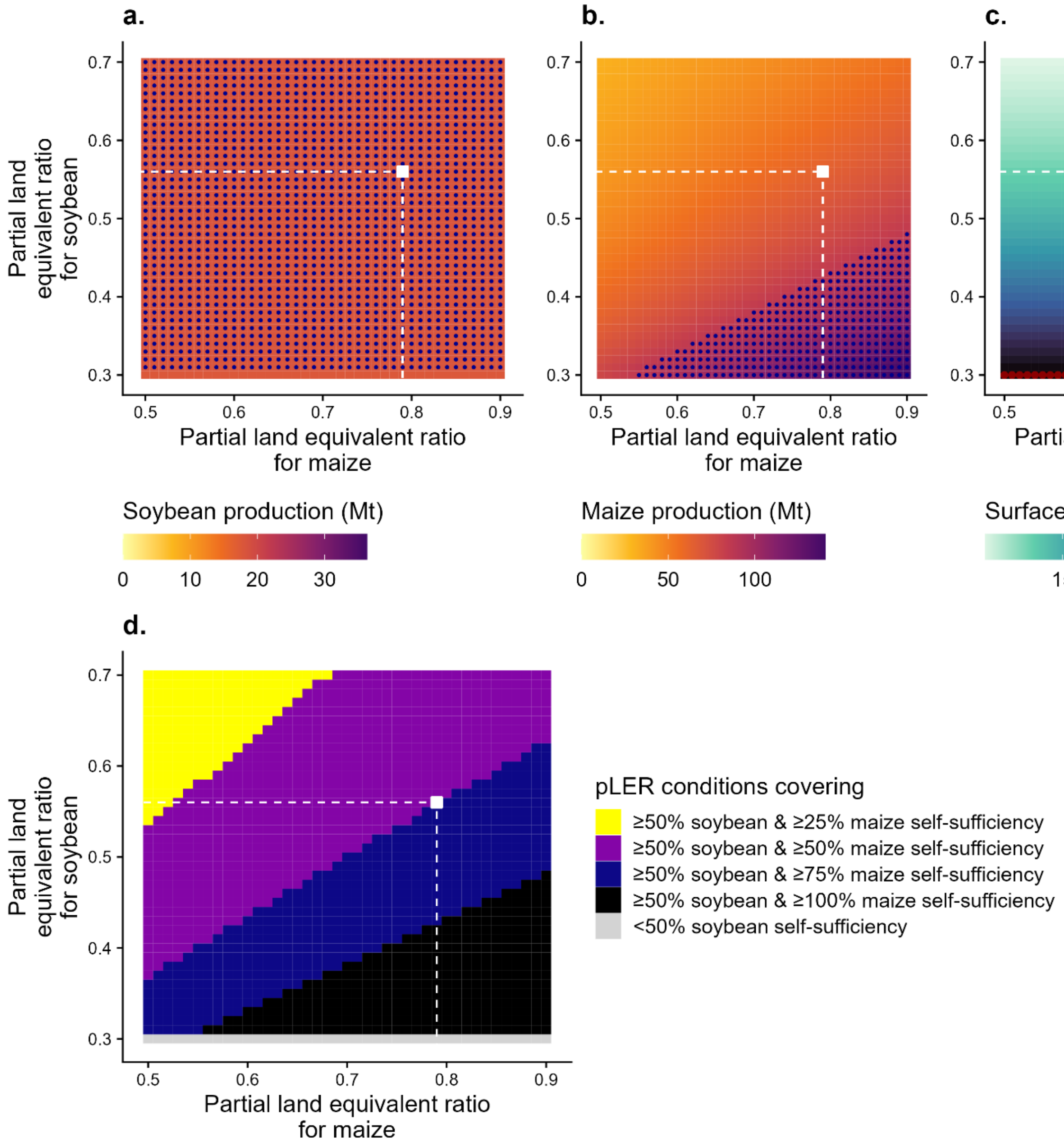

Supplementary Figure 12. Characteristics of maize-soybean intercropping allocations in terms of soybean produced (a), maize produced (b), and surface allocated (c) for covering 50% soybean self-sufficiency in the European Union depending on intercropping efficiency (pLER). The minimum efficiency conditions simultaneously satisfying 50% of soybean and 25, 50, 75, or 100% maize self-sufficiency are also shown (d). Scenarios with soybean and maize productions satisfying 50% and 100% self-sufficiency (i.e., 18.1 and 85.1 Mt, respectively) are highlighted by blue dots in panels (a) and (b), respectively. All scenarios assume a one-in-four year return frequency of intercropping and the total surface is limited to 25% of cropland area in the European Union (i.e., 25 Mha). Scenarios where the surface allocated to intercropping reaches that threshold are represented by the red dots in panel (c). In all panel, the white dot represent the current estimation of maize-intercropping efficiency at a global scale estimated by Xu et al. (2020).

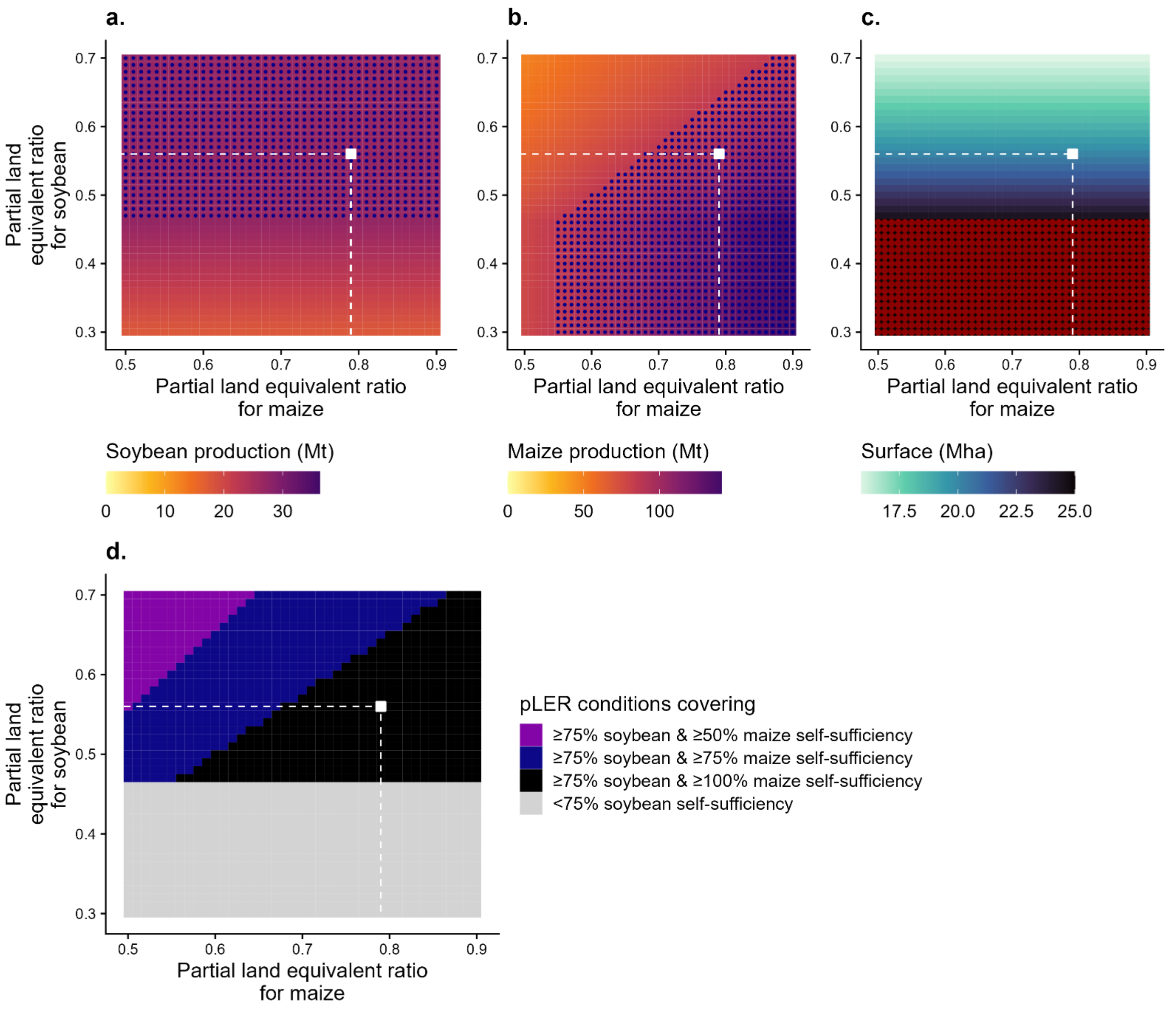

Supplementary Figure 13. Characteristics of maize-soybean intercropping allocations in terms of soybean produced (a), maize produced (b), and surface allocated (c) for covering 75% soybean self-sufficiency in the European Union depending on intercropping efficiency. The minimum efficiency conditions simultaneously satisfying 75% of soybean and 25, 50, 75, or 100% maize self-sufficiency are also shown (d). Scenarios with soybean and maize productions satisfying 50% and 100% self-sufficiency (i.e., 27.2 and 85.1 Mt, respectively) are highlighted by blue dots in panels (a) and (b), respectively. All scenarios assume a one-in-four year return frequency of intercropping and the total surface is limited to 25% of cropland area in the European Union (i.e., 25 Mha). Scenarios where the surface allocated to intercropping reaches that threshold are represented by the red dots in panel (c). In all panel, the white dot represent the current estimation of maize-intercropping efficiency at a global scale estimated by Xu et al. (2020).

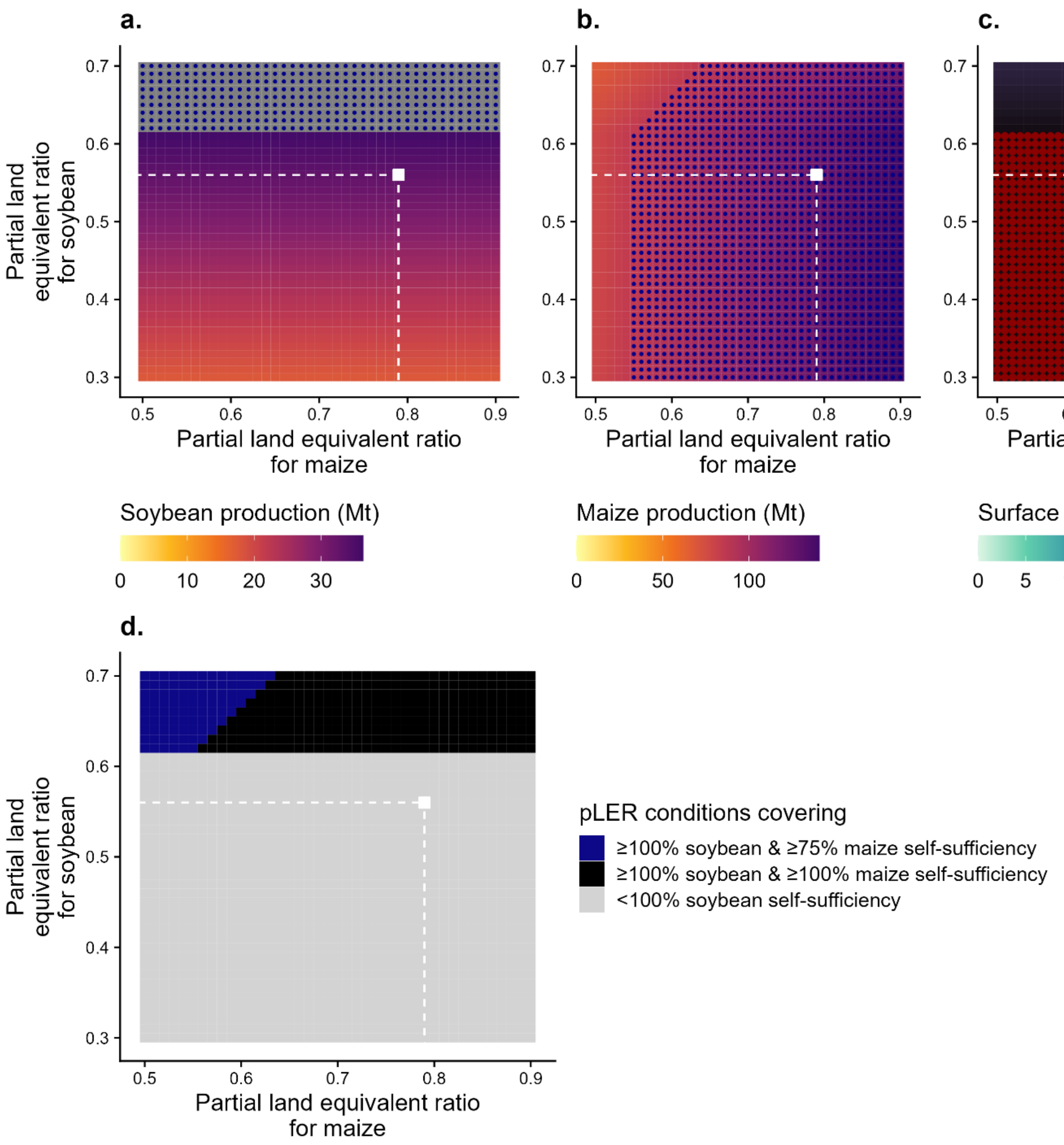

Supplementary Figure 14. Characteristics of maize-soybean intercropping allocations in terms of soybean produced (a), maize produced (b), and surface allocated (c) for covering 100% soybean self-sufficiency in the European Union depending on intercropping efficiency. The minimum efficiency conditions simultaneously satisfying 100% of soybean and 25, 50, 75, or 100% maize self-sufficiency are also shown (d). Scenarios with soybean and maize productions satisfying 100% and 100% self-sufficiency (i.e., 36.6 and 85.1 Mt, respectively) are highlighted by blue dots in panels (a) and (b), respectively. All scenarios assume a one-in-four year return frequency of intercropping and the total surface is limited to 25% of cropland area in the European Union (i.e., 25 Mha). Scenarios where the surface allocated to intercropping reaches that threshold are represented by the red dots in panel (c). In all panel, the white dot represent the current estimation of maize-intercropping efficiency at a global scale estimated by Xu et al. (2020).

Results section, lines 203-217:

“In sensitivity analyses, 25% or 50% soybean self-sufficiency was reached as long as soybean pLER was higher than 0.3 and 0.31, respectively (Supplementary Figures 11-12). Our simulations suggest that as soybean pLER increases, higher maize pLER would be required to maintain high self-sufficiency levels for both crops. In an allocation scenario targeting 50% of soybean self-sufficiency, with a maize pLER of 0.79, full maize self-sufficiency would be achieved when soybean pLER remains lower than 0.41. However, when soybean pLER exceeds this threshold, maize self-sufficiency does not exceed 75% (Supplementary Figure 12). This reflects a trade-off: as intercropping becomes more efficient for soybean production (i.e., higher soybean pLER), less land is needed to meet the soybean self-sufficiency target. Because the intercropping area is lower, maize production in intercropping is reduced. Similar patterns were obtained when targeting 25%, 75% or 100% soybean self-sufficiency (Supplementary Figures 11, 13, and 14). For higher objectives, one limiting factor was the maximum area allocated to intercropping. While three-quarters of the EU’s demand in soybean could be met with a relatively low soybean pLER of 0.46, achieving full soybean self-sufficiency using less than 25 million hectares would require a minimum pLER of 0.62 (Supplementary Figures 13-14).”

Material and methods section, lines 426-432:

"Additionally, for the different soybean self-sufficiency levels considered in this study (i.e., 25%, 50%, 75%, and 100%), we considered scenarios with pLER varying values of soybean (0.3 to 0.7, by 0.01-increment) and maize (0.5 to 0.9, by 0.01-increment) to increase the range of possible performances of intercropping over sole cropping. We estimated the minimum pLERs conditions simultaneously satisfying each soybean self-sufficiency levels, as well as 25%, 50%, 75%, and 100% maize self-sufficiency, given a one-in-four-year return frequency and limiting the surface allocated to 25% of the cropland area in the European Union.”

1.14. 211-213 I acknowledge that the authors correctly cite Chen et al. (2023) that increased sunshine hours and temperatures reduced the performance of maize-soybean intercropping. I find this a puzzling result, and without further evidence on the underlying mechanisms, I would be hesitant to accept this as a fact …

Our response: This sentence has been nuanced.

Discussion, lines 259-261:

“For example, results from a meta-analysis of field trials conducted in China (Chen et al., 2023) suggest that increased sunshine hours and temperatures would reduce the performance of maize-soybean intercropping compared to sole crops.”

Reference: Chen, G. *et al.* Potential crop yield gains under intensive soybean/maize

intercropping in China. *Plant and Soil*, doi:10.1007/s11104-023-06423-7 (2023).

1.15. 216 I had to read three times “shorter growth period overlap […] could contribute in reducing competition” before I understood it. It feels like a four-fold negation.

Our response: This sentence has been clarified.

Discussion, lines 263-265:

“However, wider strip distance (Yang et al., 2024), moderate maize planting density, or shorter growth periods’ overlap (Chen et al., 2023) could contribute in reducing the competition between species in intercrops.”

References:

Chen, G. et al. Potential crop yield gains under intensive soybean/maize intercropping in China. Plant and Soil, doi: https://doi.org/10.1007/s11104-023-06423-7 (2023).

Yang, S. *et al.* Yield performance response to field configuration of maize and soybean intercropping in China: A meta-analysis. *Field Crops Research* **306**, 109235, doi:https://doi.org/10.1016/j.fcr.2023.109235 (2024).

1.16. 239-243 Personally, I would expect more opportunity for relay intercropping and double cropping with higher temperatures though there might be some areas with production decreases due to drought under climate warming. Just a thought.

Our response: Thank you for your comment. Indeed, in the context of climate change and increase of temperatures, the growing period of crops might be shortened and the probability of success of double cropping may increase under climate change in some regions such as Brazil (Andrea et al., 2020) or the US (Massigoge et al., 2023). However, the relative interest of intercropping compared to double cropping requires further studies. This is now mentioned in the Discussion section of the revised manuscript.

Discussion, lines 305-309:

“With the possible reduction of the lengths of growing periods in the context of climate warming, the cultivation of several crops during one single growing season (i.e., relay or double cropping) might become more plausible in some regions (Andrea et al., 2020; Massigoge et al., 2023). However, a comprehensive analysis of the relative interest of these practices compared to intercropping is lacking.”

References:

Andrea, M.C.d., Dallacort, R., Tieppo, R.C. *et al.* Assessment of climate change impact on double-cropping systems. *SN Appl. Sci.* **2**, 544 (2020). https://doi.org/10.1007/s42452-020-2325-z

Massigoge, I., Carcedo, A., de Borja Reis, A. F., *et al*. Exploring avenues for agricultural intensification: a case study for maize-soybean in the Southern US region. Agricultural Systems, 204, 103539 (2023). https://doi.org/10.1016/j.agsy.2022.103539

1.17. 262 competition (singular)

Our response: Corrected.

1.18. Fig. 4 Was there a need to add the notes that the partial land equivalent ratio of the other species was fixed? It does not matter for the result shown, does it?

Our response: We agree with the reviewer and removed the notes in Figure 4.

REVIEWER #2

Remarks to the Author

2.1. The authors used machine learning models to estimate the potential contribution of intercropping soybeans with maize to improve Europe's self-sufficiency. The rationale sounds. The paper is well-written and easy to follow. The implications of the results could influence policy and research prioritization.

Our response: Thank you for your positive feedback.

2.2. However, there are too many assumptions, and the results are highly uncertain. Most importantly, the authors do not have the appropriate data to train their models on actual data from intercropping systems. Their approach to model each crop separately and then apply weights (0.56 for soybean and 0.79 for maize) to estimate intercropping yields was probably the best they could do, but, I am very skeptical of the results because they do not account for the interactions that will occur in reality by intercropping maize with soybean.

Our response: We acknowledge that given the lack of experimental trials in Europe the performance of maize-soybean intercropping in that region might be uncertain. In order to take into account this uncertainty, we performed several new sensitivity analyses with varying intercropping efficiency, as described in comment #1.13. These analyses allowed us to estimate the minimum pLERs conditions simultaneously satisfying a given level of soybean self-sufficiency (i.e., 25, 50, 75, and 100%) as well as 25, 50, 75, or 100% maize self-sufficiency, given a one-in-four-year return frequency and limiting the surface allocated to 25% of the cropland area in the European Union. In this response letter, all results are displayed above (pages 18-21 in the present response letter) as new Supplementary Figures 11-14.

For instance, the levels of 25% and 50% soybean self-sufficiency in the EU are reached as long as soybean pLER is higher than 0.3 and 0.31, respectively (Supplementary Figure 11-12). According to our results, as soybean pLER increases, higher maize pLER would be required to maintain self-sufficiency levels of 50% and 75% for soybean and maize, respectively (Supplementary Figure 12-13). Indeed, higher soybean pLER, the lower surface required by intercropping to reach a given target self-sufficiency threshold. In other words, less land is required to produce a certain quantity of soybean in a scenario where intercropping shows higher soybean pLER. In this case, as intercropping area is lower, less maize is produced by the intercropping. On the opposite, when soybean pLER is lower, more maize is produced because a higher intercropping area is required to produce the same target quantity of soybean (Figure 4). When targeting higher soybean production objectives (i.e., 75% or 100% soybean self-sufficiency, Supplementary Figures 13-14), one limiting factor is the maximum area allocated to intercropping (here limited to 25 Mha). While three-quarters of the EU's demand in soybean could be met with a relatively low soybean pLER of 0.46, achieving full soybean self-sufficiency using less than 25 million hectares would require a minimum pLER of 0.62.

The manuscript now describes these analyses (see Results section lines 203-217, and new Supplementary Figures 11-14).

2.3. Furthermore, the meta-analysis study the author cite does not reflect Europe's production environments.

Our response: Thank you for pointing this issue, which has been addressed in our response to comment #1.3, and in Supplementary Figures 21-22 of the revised manuscript.

2.4. Results from this study, which are very promising (13 Mha of intercropping could cover 50% and 75% of the EU's consumption in soybean and maize) would create expectations that may influence policy not in the right direction. While the authors did the best they could do, including sensitivity analysis to explore the impact of their assumed values, I believe yield predictions of intercropping systems at scale is a very premature task, especially in Europe where farmers have no prior experiences with soybean cultivation.

Our response: We thank the reviewer for this suggestion. Following reviewers' suggestions, we added two new analyses to assess the sensitivity of the level of self-sufficiency to N inputs and to temporal differentiation niche. These new analyses showed that our conclusion was only marginally changed, even in a high N inputs scenario and in case of strong competition between both species. The reviewer can refer to our response to the comment #1.2., as well as Supplementary Figure 10 in the revised manuscript, lines 155-164 and 188-193 in the revised manuscript.

REVIEWER #3

Remarks to the Author

3.1. The manuscript entitled "High potential contribution of intercropping to soybean and maize self-sufficiency in Europe" evaluates the cropland requirements needed to achieve 50% and 75% self-sufficiency in soybean and maize production across the EU. The authors demonstrate that intercropping could reduce land demand by 27–30% compared to sole cropping systems. To estimate yields, they develop a Random Forest-based machine learning model trained using GDHY (yield), SPAM2010 (irrigation), and ERA5 (weather) datasets, and apply a yield-threshold-based spatial allocation approach to identify suitable growing regions. Cropland is allocated using a soybean-first, yield-ranked assignment strategy, and intercropping performance is parameterized using partial Land Equivalent Ratio (pLER) values reported in Xu et al. (2020, Field Crops Research).

Our response: Thank you for this summary of our study.

3.2. While the modeling framework is technically sound and clearly described, the main findings are somewhat expected. Given the adopted pLERs of 0.56 for soybean and 0.79 for maize (total land saving ≈ 0.56 + 0.79 – 1 ≈ 0.3), the reported 27–30% land saving aligns with theoretical expectations.

Our response: We thank the reviewer for this comment, which was also pointed by Reviewer 1 (see our response to comment #1.5.). In the new Supplementary Material 1, we show that the results are not obvious due to the yield variability in Europe (Figure 1). As some areas are more productive than others, the allocation of the crop species in the most productive areas may make monocropping systems more productive than expected according to LER. Although the spatial variability might be relatively low at the field scale, this is an important aspect to consider at large scales.

3.3. However, as acknowledged by the authors in Line 202, intercropping efficiency is highly variable (ranging from 0.5 to 2.0) depending on local environmental conditions and cropping system configurations. This variability introduces substantial uncertainty that is not fully explored.

Our response: We acknowledge that given the lack of experimental trials in Europe the performance of maize-soybean intercropping in that region might be uncertain. In order to take into account this uncertainty, we performed additional sensitivity analyses which

1) included the potential impact of farming practices on intercropping efficiency (i.e., high N input and high competition between species) and
2) considered a refined range of pLER values for soybean and maize (from 0.3 to 0.7 and from 0.5 to 0.9, by 0.01-increment, respectively).

These new analyses showed that our conclusion marginally changed in a high N inputs scenario and in case of strong competition between both species (see our response to the comment #1.2., as well as Supplementary Figure 10) and in case of lower pLER values (see our response to the comment #2.2., as well as Supplementary Figures 11-15). These sensitivity analyses allowed for identifying a trade-off between maize and soybean production in intercropping, which is now discussed in the revised manuscript.

3.4. Moreover, the study does not address practical constraints of intercropping—such as increased harvesting difficulty, crop protection, and irrigation complexity—which could limit real-world adoption and should be discussed.

Our response: Thank you for this comment. Following the reviewer's comment, we expanded the discussion on these aspects.

Discussion, lines 276-279:

"For instance, intercropping requires specific machinery for sowing, harvesting, and sorting of grains of the different crops to comply with collectors and processors purity standards. In addition, adapted irrigation systems and crop protection practices still need to be identified, also according to intercropping design."

3.5. Regarding the GDHY dataset used to train the yield model, there appears to be a significant limitation for soybean. According to Supplementary Figure 1, very few data points from Europe were used in model training for soybean, which may affect prediction accuracy. This important limitation should be explicitly acknowledged in the discussion section.

Our response: We thank the reviewer for pointing this limitation, which is now mentioned in the Discussion of the revised manuscript.

Discussion, lines 288-294:

"One strength of this study is the use of a machine learning model to model soybean and maize productivity trained on a global historical yield dataset (Iizumi and Sakai, 2020). Thanks to their greater flexibility, machine learning algorithms often outperform traditional methods (e.g., linear regression or process-based models) to forecast yields of crops from climate predictors, especially soybean (Guilpart et al., 2022; Chen et al., 2024) and maize (Abramoff et al., 2023). For soybean, a limitation of the model is the low number of observations collected in Europe as few data points were available in Europe to train the model for soybean, which may affect prediction accuracy."

References:

Abramoff, R. Z. et al. Adaptation Strategies Strongly Reduce the Future Impacts of

Climate Change on Simulated Crop Yields. Earth's Future 11, e2022EF003190, doi:https://doi.org/10.1029/2022EF003190 (2023).

Chen, M., Guilpart, N. & Makowski, D. Comparison of methods to aggregate climate data to predict crop yield: an application to soybean. Environmental Research Letters 19, 054049, doi:10.1088/1748-9326/ad42b5 (2024).

Guilpart, N., Iizumi, T. & Makowski, D. Data-driven projections suggest large opportunities to improve Europe's soybean self-sufficiency under climate change. Nature Food 3, 255-265, doi:10.1038/s43016-022-00481-3 (2022).

Iizumi, T. & Sakai, T. The global dataset of historical yields for major crops 1981-2016. Sci Data 7, 97, doi:10.1038/s41597-020-0433-7 (2020).

Specific Comments:

3.6. Line 52–53: Wu et al. (2023) does not report a doubling of production, but rather a 67.4 million ton increase on top of the current 19 million tons, which is closer to a fourfold increase. Please correct this statement.

Our response: Thank you for this correction. Thank you for your comment. Wu et al. (2023) report that intercropping maize with soybean on 50% of the maize planting area in China in 2018 could more than double the total soybean production in this country. This latter results is now provided in the revised version of the manuscript.

Introduction, lines 52-53:

"In China, intercropping maize with soybean on 50% of the maize planting area could more than double the total soybean production in 2018 in this country (Wu et al., 2023)."

Reference: Wu, Y. et al. Soybean yield variations and the potential of intercropping to increase production in China. Field Crops Research 291, 108771, doi:https://doi.org/10.1016/j.fcr.2022.108771 (2023).

3.7. Line 187–189: "Our study is the first providing evidence on the benefits of intercropping…" This claim appears overstated. The study does not present new experimental evidence but rather extends the findings from Xu et al. (2020) to a European context through modeling. Please consider rephrasing this claim to reflect that the analysis is based on extrapolated meta-analytical data.

Our response: Thank you, this sentence has been nuanced.

Discussion, lines 232-235:

"Extending the results from previous meta-analysis by taking into account spatial variability of intercropping in the EU, our study estimates the benefits of intercropping to improve EU's self-sufficiency in soybean, while maintaining high production of maize, and to quantify land saved by this strategy over sole cropping at a continental scale."

3.8. Line 204: Please clarify how intercropping is expected to be beneficial in areas where the sum of soybean and maize pLER values is less than 0.5. Such values would indicate very low productivity under intercropping.

Our response: As stated in the discussion section, the performance of intercropping can be very variable (Xu et al., 2020). We agree with the reviewer that a LER (i.e., the sum of pLERs) of 0.5 would induce a very low intercropping productivity.

In this study, we examine LER values ranking from 0.8 (pLERs of 0.3 and 0.5 for soybean and maize, respectively) to 1.6 (pLERs of 0.7 and 0.9 for soybean and maize, respectively), which are in the range of the possible values of maize-soybean LER estimated by Xu et al. (2020). Our results showed that, assuming a return-frequency of one-in-four-year, a minimum pLER of 0.3 for soybean in required to reach at least 25% of self-sufficiency in the EU, but would require more land, as shown in the new sensitivity analyses presented in Supplementary Figures 11 to 15.

This was clarified in the revised manuscript.

Material and methods, lines 410-415:

"To assess the sensitivity of our results to cropping frequency, the main analysis (i.e., pLER values of 0.56 for soybean and 0.79 for maize, respectively) was repeated in scenarios specified by combining different values of crop frequencies (one-in-seven to one-in-two years, by yearly increment), soybean pLER (0.3 to 0.7, by 0.1-increment) and maize pLER (0.5 to 0.9, by 0.1-increment). These values of pLERs corresponded to LER ranging from 0.8 to 1.6, in the range of the possible values of maize-soybean LER estimated by Xu et al. (2020)."

Reference: Xu, Z. et al. Intercropping maize and soybean increases efficiency of land and fertilizer nitrogen use; A meta-analysis. Field Crops Research 246, 107661, doi:https://doi.org/10.1016/j.fcr.2019.107661 (2020).

3.9. Lines 318–319: The reference to "25%, 50%, 75%, or 100% of the average annual soybean consumption" is inconsistent with the scenarios described in the abstract, which

only refer to 50% and 75% self-sufficiency. Please ensure consistency throughout the manuscript.

Our response: Thanks for your comment. The abstract now refers to all four scenarios explored in the study. In addition, the consistency has been checked throughout the manuscript.

Abstract, lines 16-18:

"Maize–soybean intercropping, even limited to 25% of croplands in the European Union, could meet 25%, 50%, and 75% of soybean demand with 6.3, 13.1, and 20.1 Mha, respectively. Full self-sufficiency would require exceeding this threshold."

3.10. Line 322: Please clarify the rationale for selecting 25 Mha (25% of EU cropland) as the maximum allocation limit to avoid competition with other crops. Is this threshold based on existing land-use statistics, policy guidelines, or modeling assumptions? It would also be helpful to indicate what percentage of EU cropland is currently allocated to maize and soybean combined, for context.

Our response: Thank you for your comment. In our study, we suppose that the area allocated to soybean would not exceed the area currently cultivated with wheat, which is the most cultivated crop in Europe. On average between 2018-2022, wheat approximatively occupied 25 Mha (source: Eurostats), an area representing 25% of the cropland in the EU. This is now clarified in the manuscript.

Reference: Eurostats. Farms and hectares by type of crops, utilised agricultural area, economic size and NUTS 2 region [ef_lus_allcrops__custom_17642986] [update: February 27, 2024]. Accessed in August 2025.

Material and methods, lines 383-389:

"For each self-sufficiency scenario, maize-soybean intercropping was sequentially allocated to highest yielding sites, ranked by soybean productivity (i.e., projected soybean yields), until P_S^* was reached or until the total area allocated to intercropping reached a maximum threshold, chosen to limit competition with other crops. We supposed that the area allocated to soybean would not exceed 25 Mha, which corresponds to 25% of EU croplands in 2018 and to the area dedicated to wheat, which is currently the most cultivated crop in Europe (average 2018-2022, Eurostats)."

3.11. Line 491 / Figure 1: Why does the yield ratio map (Figure 1c) show values in the Nordic countries where the individual maize and soybean yield maps (Figures 1a and 1b) contain no data? Additionally, what explains the hotspot around Switzerland in Figure 1c, where maize yields are high while soybean yields are extremely low?

Our response: Thank you for spotting these inconsistencies, which are linked to the way that the yield ratio has been computed (i.e., ratio between average maize yield and soybean yield in each site). Yields in the Nordic countries are very low for both crops (~0 $t.ha^{-1}$), but a ratio of two low values can be high (example for one site in Finland: maize yield was 0.367 $t.ha^{-1}$, while soybean yield was 0.11 $t.ha^{-1}$: the yield ratio in this case was 0.367/0.11=3.3). Similarly, the areas around Switzerland correspond to areas where the yield of soybean is, indeed, very low (<1 $t.ha^{-1}$), while the maize yield are high. This is why the ratio between maize and soybean yields in these areas is higher (example for one site in Switzerland: maize yield was 6.87 $t.ha^{-1}$, while soybean yield was 0.568 $t.ha^{-1}$: the yield ratio in this case was 6.87/0.568=12.1).

To improve the consistency between the yield maps shown in Figures 1a and 1b and to avoid confusion, we adapted to computation of the yield ratio with the following conditions: in areas with very low maize and soybean yields (i.e., <0.5 $t.ha^{-1}$), the ratio was also set to 0. In addition, the areas with low soybean yields (i.e., <1 $t.ha^{-1}$) but moderate-to-high maize yields (i.e., >6 $t.ha^{-1}$), characterized by very high yield ratios (>8) were highlighted in dark grey.

Please see the new version of Figure 1, also displayed below, and associated caption (with the changes highlighted in yellow).

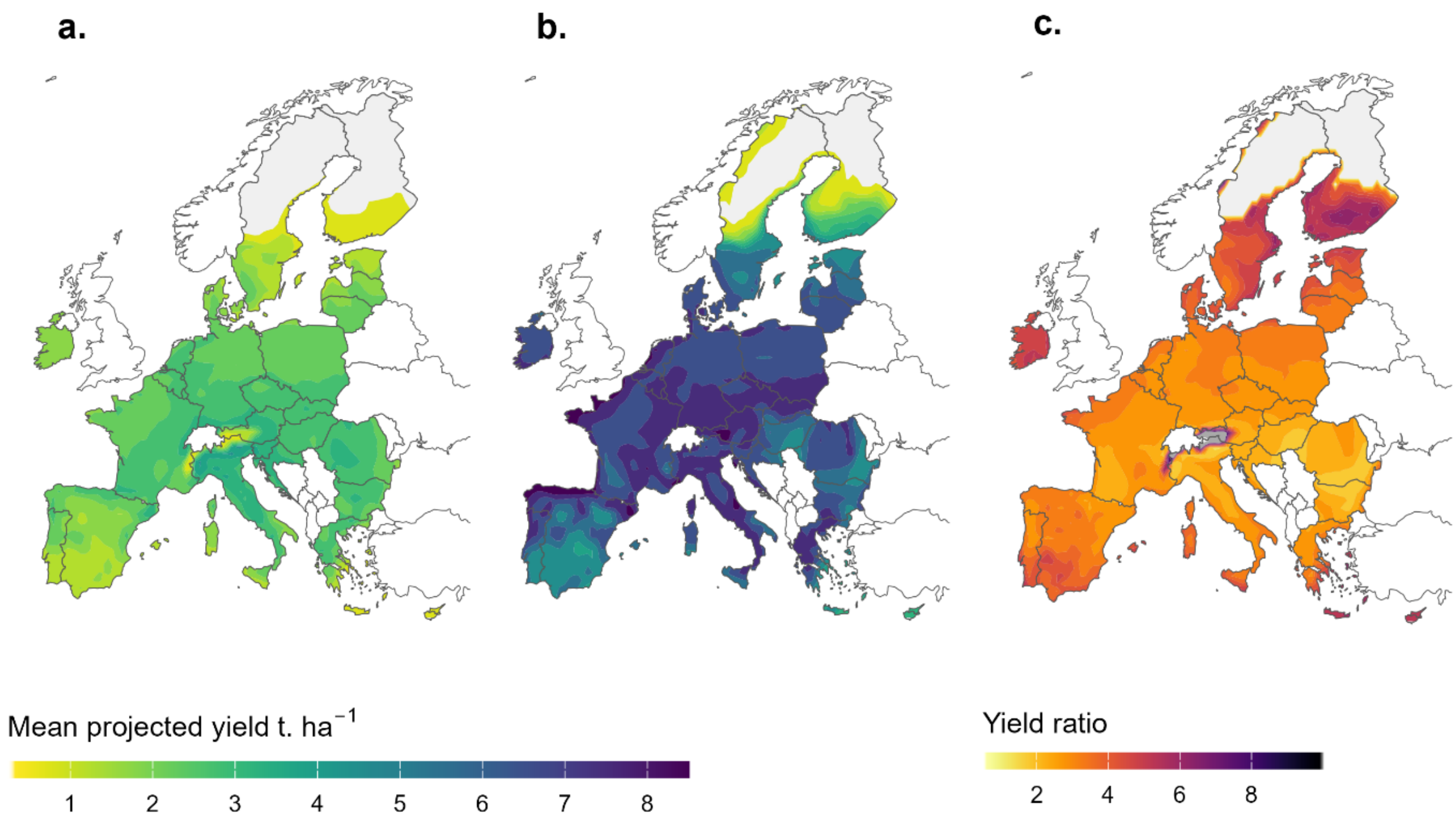

Figure 1. Soybean (a) and maize (b) rainfed yield projections (2000-2023 averages), and ratio maize/soybean (c) in the European Union. Projections were obtained from random forest models based on climate inputs (derived from the ERA5-land dataset) covering the crop growing seasons (April-November for soybean and April-December for maize) and irrigated fraction (estimated by SPAM2010, and set to zero for the yield projections). Climate inputs included monthly averages of minimum and maximum temperatures, total precipitation, solar radiation, reference evapotranspiration, and vapor pressure deficit. For each climate variable, the first two scores derived from a principal component analysis were used as climate predictors in the model. For panels (a) and (b), areas with average crop yield ~ 0 t.ha-1 are displayed in grey. For panel (c), the yield ratio was set to 0 in areas with very low maize and soybean yields (i.e., <0.5 t.ha-1), and areas close to the Alps with low soybean yields (i.e., < 1 t.ha-1) but moderate-to-high maize yields (i.e., > 6 t.ha-1), characterized by the highest yield ratios, were indicated as dark grey.

3.12. Figures 2 & 3: Please ensure that the legend color schemes—particularly the yellow class—are used consistently between Figures 2 and 3. Any inconsistency in classification or color meaning should be clarified.

Our response: Thank you. The legend color scheme has been homogenized among Figures 2 and 3 (we removed the grey line previously displayed on the yellow bars in Figure 2). Please see the new version of Figure 2.

Remarks on code availability:

3.13. I was unable to access the code at the provided link. When I attempted to review it on May 10, 2025, the repository appeared to have been removed or was otherwise inaccessible. I also examined an alternative repository (https://github.com/MathildeChen/SOYBEAN_PRED_COMP), which contains the code but includes minimal documentation and no accompanying data.

Our response: We thank the reviewer for pointing this aspect.

The code associated with the present study will be publicly available depending on the review process and when the results will be published. We acknowledge that the repository associated with the machine learning developed for the study of Chen et al. (2024) lacked of documentation. This issue is now fixed and the documentation of this repository has been consolidated.

All data used for this paper has been published in previous articles. This is now clearly stated in the revised version of the manuscript.

Material and methods, lines 441-443:

"All data analyzed in this paper are publicly available in associated publications, including maize and soybean historical yields (Iizumi and Sakai, 2020), water management (Yu et al., 2020), and climate conditions (Muñoz-Sabater et al., 2021)."

References:

Iizumi, T. & Sakai, T. The global dataset of historical yields for major crops 1981-2016. Sci Data 7, 97, doi:10.1038/s41597-020-0433-7 (2020).

Muñoz-Sabater, J. et al. ERA5-Land: a state-of-the-art global reanalysis dataset for land applications. Earth Syst. Sci. Data 13, 4349-4383, doi:10.5194/essd-13-4349-2021 (2021).

Yu, Q. et al. A cultivated planet in 2010 – Part 2: The global gridded agricultural-production maps. Earth Syst. Sci. Data 12, 3545-3572, doi:10.5194/essd-12-3545-2020 (2020).

Manuscript reference: NCOMMS-25-06543-A entitled “High potential contribution of intercropping to soybean and maize self-sufficiency in Europe”

REVIEWER COMMENTS

Reviewer #1

Remarks to the Author

1.1. I have commented on the original version of this manuscript and expressed as my opinion that I agree with the authors on the high potential contribution of intercropping to soybean and maize self-sufficiency in Europe, but I felt the analysis was not strong enough and did not adequately address uncertainties in the estimation of intercrop performance. The original analysis was based on machine learning models to estimate the soybean and maize sole crop yields across Europe, while values of soybean pLER and maize pLER from a meta-analysis by Xu et al. (2020) were used to extrapolate these crop yields to production in intercrops. I found that analysis too simple in addressing the uncertainties in intercrop productivity as compared to sole crops, given the huge variation in intercrop performance compared to sole crops reported in the literature. The authors have responded satisfactorily to this criticism in their response letter. Based on the response letter, I understand that in the revised paper, different pLER values for maize and soybean are used under the assumption of simultaneous vs relay intercropping and under the assumption of “average N input, as in the meta-analysis” as compared to a European estimate of N input, based on the 75 percentile of N input to maize in Europe from Eurostat. This sounds good.

Our response: We thank the reviewer for this summary, and we are glad that the sensitivity analysis performed for the first revision of our study satisfies him/her.

1.2. My comments are now mainly on the way these things are written up in the manuscript, which I found lacking in clarity. I suppose that with rigorous editing, the linkage between results, concepts and methods can be improved. Three key things did not become clear to me:

(1) is soybean allocated to the cropland that gives the highest sole soybean yields in Europe according to the ML model, by first ranking all sites according to the soybean yield and then selecting that number of sites that would result in sufficient production (area times yield) to reach a certain production target, e.g. 25 or 50% self

sufficiency), or is soybean allocated evenly to all the land that is climatically suitable for soybean cultivation? (Actually, it is stated in the M&M at the end of the paper, but readers need to understand it earlier, when they read the results)

(2) the land that is allocated to soybean was previously allocated to another crop; it did not become clear to me which crop was replaced. I think this needs clearer description and possibly discussion regarding the consequences of substitution.

(3) The authors used regressions from Xu et al. (2020) on the effects of N input and simultaneous vs relay intercropping to assess the relative performance of intercrops compared to sole crops under European growing conditions, and then explored whether these relationships affected their main thesis: maize-soybean intercropping has high potential for increasing self-sufficiency for soybean in Europe. It was not found to be sensitive. I wonder whether it would be possible to illustrate this lack of sensitivity in the main body of the paper graphically.

Our response: We thank the reviewer for pointing out on these issues.

For the issue (1), we included more details on the land allocation procedure in the Results section, and the reader can now understand better the methodology employed without the need to read the Material and methods first. In addition, we improved the Figure 2 in order to provide more details on the methodology applied in the study when reading the Results section. See also our answers to comments 1.20, 1.25, 1.26, and 1.58.

For issue (2), we have quantified how much wheat, maize, barley, rapeseed, and sunflower were grown in the regions where projected soybean yield was low, moderate, or high. For more details on this issue, please refer to comment 1.15.

Regarding the issue (3), we now assess the robustness of our estimates in the Supplementary Figures 11, which has been updated with a new sensitivity analysis to take into account the spatial variability of N fertilizer use, as suggested by the reviewer #3. Please also see our response to comment 2.4.

Detailed comments

Related to the abstract

1.3. Please note that detailed comments were written “as I went”. It means that some queries might be answered later in the text, e.g. regarding methods used to produce the

results. I chose not to edit these comments because the paper needs to be written in such a way that the readers knows enough about methods that such questions would not arise.

Our response: Noted, thank you.

1.4. 16 “and by minimizing crop substitution” At this point in the paper, it is not clear what is meant. When reading further, I am not sure we did get information on the degree of substitution. Maybe this is implied somehow, but it is not explicit.

Our response: The abstract refers to the risk of substitution of maize by soybean in the EU for the purpose of increasing of soybean self-sufficiency. The sentence has been modified to clarify this point.

Abstract (lines 14-17):

“The European Union is currently not self-sufficient in soybean and maize. Increasing domestic production of one of these species could reduce the production of the other. We examine the potential for maize-soybean intercropping to improve the European Union's soybean self-sufficiency while minimizing competition with maize and other crops.”

1.5. 16-18 “Maize-soybean intercropping, even limited to 25% of croplands in the EU, could meet 25%, 50%, and 75% of soybean demand with 6.3, 13.1, and 20.1 Mha, respectively”. This is confusing as written. Why is there a constraint of “25% of cropland in the EU” in this sentence? Suggest to rephrase for clarity. e.g. Cultivation of maize-soybean intercrops on 6.3, 13.1, and 20.1 Mha of European cropland could meet 25, 50 and 75% of soybean demand, respectively. Maybe I misunderstand …

Our response: The objective of our study is to investigate whether growing maize-soybean intercropping in the EU could improve self-sufficiency while limiting cropland use to avoid competition with other crops. The sentence has been rephrased to reflect this objective.

Abstract (lines 15-21):

“We examine the potential for maize-soybean intercropping to improve the European Union's soybean self-sufficiency while minimizing competition with maize and other crops. Assuming a one-in-four years frequency, 25%, 50%, and 75% self-sufficiency would be achieved with 6.3, 13.1, and 20.1 Mha of maize-soybean intercropping, resulting in total co-production of 37.9, 81.9, and 126.2 Mt of both crops, respectively. Meeting 100% of

European soybean demand (i.e., 36.3 Mt) would require growing intercropping on more than 25% of European cropland (25 Mha) every year."

1.6. 19 Which threshold is meant? You mean "Greater self-sufficiency than 75% would require that more than 25% of European cropland was used for maize-soybean intercropping"? I am not sure whether you mean that in any one year, more than 25% of the EU crop land would be under maize-soybean intercropping (MSI), or you mean that more than 25% of EU crop land would be cultivated with MSI in one out of four years, i.e. more than 6.25% under MSI in any one year. This aspect needs to be clarified.

Our response: Following the reviewer's comments 1.5 and 1.6, the sentence has been clarified (lines 20-21).

1.7. 19-21 This sentence is unclear: "When comparing […] than in 0.5 ha of sole soybean". Please rephrase.

Our response: The sentence has been improved for clarity.

Abstract (lines 24-25):

"These estimates held provided that soybean production from 1 hectare of intercropping exceeded that from 0.5 ha of sole soybean."

Related to the introduction

1.8. 29 I think that instead of "became" you might say here "has become" (as of now) or add the year by when this became so.

Our response: Corrected (line 33).

1.9. 43-44 I suppose this information that soybean consumes more water than maize comes straight from the cited paper that two of the authors co-authored, but I am surprised. Based on my knowledge and experience, I thought that the water use by maize is usually a little greater than that of soybean. This opinion was backed up when I used chatgpt to find information. Please check.

[Text Redacted]

So, in summary: yes, published literature generally supports maize using somewhat more water (or at least achieving higher yields per water) under many conditions, but it is not a universal rule and depends on context.

Our response: We thank the reviewer for this comment. We agree that evapotranspiration of maize is often slightly higher than those of soybean, although this can vary according to local conditions as the reviewer pointed out. However, more evapotranspiration does not translate to more irrigation, systematically. For example, according to a large survey of farming practices performed in 2021 in France, when irrigated, soybean received on average 100 mm of irrigation water (n=1500) against 126 mm for maize grain (n=3385), and 99 mm for silage maize (n=2429) (Service de la Statistique et de la Prospective, 2021).

In their paper, Lauerwald et al. (2023) estimated the variation in water supply derived from the conversion of maize crops to soybean in the EU. Water supply is the sum of available soil water at sowing, in-season precipitation, and total irrigation (Grassini et al., 2015). Because soybean's root system was assumed to be shallower compared to the one of maize, growing soybean was found to require more water in the European context, which mainly explained the conclusions of Lauerwald et al. (2023). Sensitivity analyses considering similar root systems between both crops slightly reduced the estimated water deficit of a maize-to-soybean conversion. In addition, these results were sensible to sowing dates.

Intercropping maize and soybean was found to improve water use efficiency of maize (Ren et al., 2017), especially in semi-arid conditions (Raza et al., 2021). However, the present study does not examine the effect of maize-soybean intercropping on water stress or irrigation needs.

We acknowledge that the way the sentence was written might confuse the reader, who would expect some results on this topic. For this reason, we removed the sentence to make the introduction clearer.

References:

Grassini, P., Torrion, J.A., Yang, H.S., Rees, J., Andersen, D., Cassman, K.G., Specht, J.E. Soybean yield gaps and water productivity in the western U.S. Corn Belt Field Crops Res., 179, 150-163. doi:https://doi.org/10.1016/j.fcr.2015.04.015 (2015).

Lauerwald, R., Guilpart, N., Ciais, P. & Makowski, D. Impact of a large-scale replacement of maize by soybean on water deficit in Europe. Agricultural and Forest Meteorology 343, 109781, doi:https://doi.org/10.1016/j.agrformet.2023.109781 (2023).

Raza, M. A., Gul, H., Wang, J., Yasin, H. S., Qin, R., Khalid, M. H. B., ... & Yang, W.. Land productivity and water use efficiency of maize-soybean strip intercropping systems in semi-arid areas: A case study in Punjab Province, Pakistan. Journal of Cleaner Production, 308, 127282. doi;https://doi.org/10.1016/j.jclepro.2021.127282 (2021).

Ren, Y.Y., Wang, X.L., Zhang, S.Q. et al. Influence of spatial arrangement in maize-soybean intercropping on root growth and water use efficiency. Plant Soil 415, 131–144. doi:https://doi.org/10.1007/s11104-016-3143-3 (2017).

Service de la Statistique et Prospective. Enquête Pratiques culturales en grandes cultures 2021 - Principaux résultats, N°2024-13. Available at: https://agreste.agriculture.gouv.fr/agreste-web/disaron/Chd2413/detail/ (2024).

1.10. 64-71 The text in these lines seems to suggest that the LER sets a maximum to the intercropping advantage that may be attained in reality. However, the LER that we have from experiments is an average across different conditions that might include those that the authors mention here, including cases where one of the sole crop does not or hardly perform; hence I wonder whether the conclusion would still hold if the LER is not a fixed mathematical property but the outcome of variable sole crop and intercrop performance across growing conditions and ecoregions.

Our response: We thank the reviewer for mentioning this point.

In our study, we assess the robustness of our conclusions to several factors. First, by modeling sole crop yields (Figure 1), our analyses include the variability in sole crop performances across the EU. Second, we performed several sensitivity analyses considering the most influential factors of pLERs (i.e., N fertilizer and TND). These sensitivity analyses make us confident about the strong benefit of intercropping vs sole cropping for improving maize and soybean self-sufficiency while reducing the area required at the EU level.

We acknowledge that substantial variability might occur at the local scale. Local data and further research are required to better optimize intercropping implementation. We are aware that some important factors are not included in our sensitivity analyses (i.e., relative density, environmental conditions) (Ruillé et al., 2026). However, comprehensive datasets to model

pLERs according to these factors are currently not available to the best of our knowledge. This is acknowledged in the discussion section of the manuscript (lines 292-305).

See also our response to comment 2.4 and in the revised manuscript the results of the sensitivity analysis considering TND and N fertilizer are presented in Supplementary Table 4 and Supplementary Figure 10.

Discussion (lines 292-305):

"Many factors affecting the performances of intercropping have been identified in previous research. […] Given the limited information on the interaction between soybean productivity in intercropping and other farming practices in Europe, additional trials are required to refine local estimations of maize-soybean intercropping performances and to help identifying the best combinations of field configuration, fertilization, and water management which maximizes the productivity of intercropping at the farm level."

Reference: Ruillé, M., Beillouin, D. & Prudhomme, R. Ecological drivers of intercropping performance for enhanced global crop production. npj Sustain. Agric. 4, 8 (2026). https://doi.org/10.1038/s44264-025-00110-z

1.11. 77 quantified -> quantify

Our response: Corrected (line 79).

Related to the results

1.12. 87 "rainfed yields". Suggest to add: "of soybean and maize" for clarity.

Our response: Added to the revised manuscript (line 89).

1.13. 96-111 This section is not so clear. I understand that you use a minimum soybean yield of 1 t/ha but no minimum for maize. Why no minimum for maize?

Our response: The rationale behind this choice is linked to objective of our study (focused on improving EU soybean production) and to the intercropping allocation process detailed in further sections. Indeed, we preferably allocated maize-soybean intercropping in areas with suitable conditions for soybean production. We considered that suitable areas were those where the projected yield of soybean was at least equal to 1.0 $t.ha^{-1}$, which is the minimum observed soybean yield in major producing regions of our training dataset with climate conditions similar to those in the EU (i.e., the US and Italy).

Additionally, the minimum maize yields were 4.0, 3.9, 3.7, and 3.7 $t.ha^{-1}$ in the areas allocated to maize-soybean intercropping (assuming a one-in-four year crop frequency and pLERs of 0.56 and 0.79 for soybean and maize, respectively) to reach 25, 50, 75, or 100% soybean self-sufficiency, respectively. These values are three to four times higher than the lowest value for maize yield (i.e., 1.3 $t.ha^{-1}$ in Bulgaria) observed in the EU in our training dataset (see panel b. Supplementary Figure 20). This justified to not set a minimum value for maize productivity in the allocation procedure applied in this study.

These reasons are now explained in the revised manuscript.

Results (lines 99-107):

"The 25th, 50th (i.e., median), and 75th percentiles of averages soybean yield projections for 2000-2023 across the EU are 1.0, 2.1, and 2.6 $t.ha^{-1}$, respectively (Figure 1a, top panel). These levels of productivity are reached over 103, 82 and 36 Mha, respectively (Figure 1a, bottom panel), where maize is the major spring crop currently cultivated (Supplementary Table 2). The level of productivity of 1.0 $t.ha^{-1}$ also corresponds to the minimum yield observed in major soybean-producing regions with climatic conditions comparable to those of the EU of our train dataset (i.e., Italy and the US). Considering this threshold as a criterion for soybean suitability, our projections suggest that 103 Mha could be considered as suitable for soybean production in the EU, largely exceeding the current European production area of 1 Mha (Supplementary Table 1).

Material and methods (lines 387-389):

"The minimum yields observed in the train dataset for major regions producing soybean with climatic conditions comparable to those of the EU (i.e., Italy and the US) and major maize-producing regions in the EU were 1.0 and 1.3 $t.ha^{-1}$, respectively."

Material and methods (lines 435-440):

"In the area allocated to maize-soybean intercropping, the minimum maize yields projections (ranging from 3.7 to 4.0 $t.ha^{-1}$ depending on the considered scenario) were three-to-four times higher than the minimum yield of 1.3 $t.ha^{-1}$ observed in our train dataset of maize-producing regions in the EU (Supplementary Figure 20). Considering this, no additional criterion was considered for maize suitability in the allocation procedure."

1.14. 99 “and to the 25th percentile of our projections under rainfed conditions” What is meant? Do you use the 25 percentile of the rainfed soybean yield projections to say that all the areas with greater yield are suitable? Why is the 25 percentile used and why do you not use the same approach and same percentile for maize? Rationale missing. Then the median of the remaining 75% of yield projections of soybean is 2.1 t/ha? It needs to be written up in a less ambiguous way. It is key to write up in a consistent way how you define where soybean and maize may be grown in the future.

Our response: In this section, we described the variability of the averages yield projected over the 2000-2023 period in the EU by their 25th, 50th (median), and 75th percentiles (i.e., 1.0, 2.1, and 2.6 $t.ha^{-1}$ for soybean, respectively; 4.3, 6.2, and 6.9 $t.ha^{-1}$ for maize, respectively) and we estimated the areas where average yield projections reach these levels of productivity (e.g., 103, 82, and 36 Mha for soybean, respectively; 100, 62, and 27 Mha for maize, respectively). To estimate the suitability of soybean cultivation in the reion, we used the minimum yield value observed in major soybean-producing areas with similar climatic conditions than the EU. In our case, these areas are Italy and the US, and the minimum observed value was 1 $t.ha^{-1}$.

We improved this paragraph to make it clearer. In particular, we describe average yield projections in a homogenous way for both soybean and maize, and we explained why we used the threshold of 1 $t.ha^{-1}$ as a criterion to define soybean suitability. In addition, we now show the distribution of average yield projections in Figure 1 (to panels) and highlighted the areas where yields reach the 25th, 50th, and 75th percentiles in the map (bottom panels). We hope that these modifications make this section easiest to read.

Results (lines 99-112):

“The 25th, 50th (i.e., median), and 75th percentiles of averages soybean yield projections for 2000-2023 across the EU are 1.0, 2.1, and 2.6 $t.ha^{-1}$, respectively (Figure 1a, top panel). These levels of productivity are reached over 103, 82 and 36 Mha, respectively (Figure 1a, bottom panel) […]. For maize under rainfed conditions, the corresponding 25th, 50th, and 75th percentiles of projected yields across the EU are 4.3, 6.2, and 6.9 $t.ha^{-1}$ (Figure 1b, top panel), attained over areas of 100, 62, and 27 Mha, respectively (Figure 1b, bottom panel).”

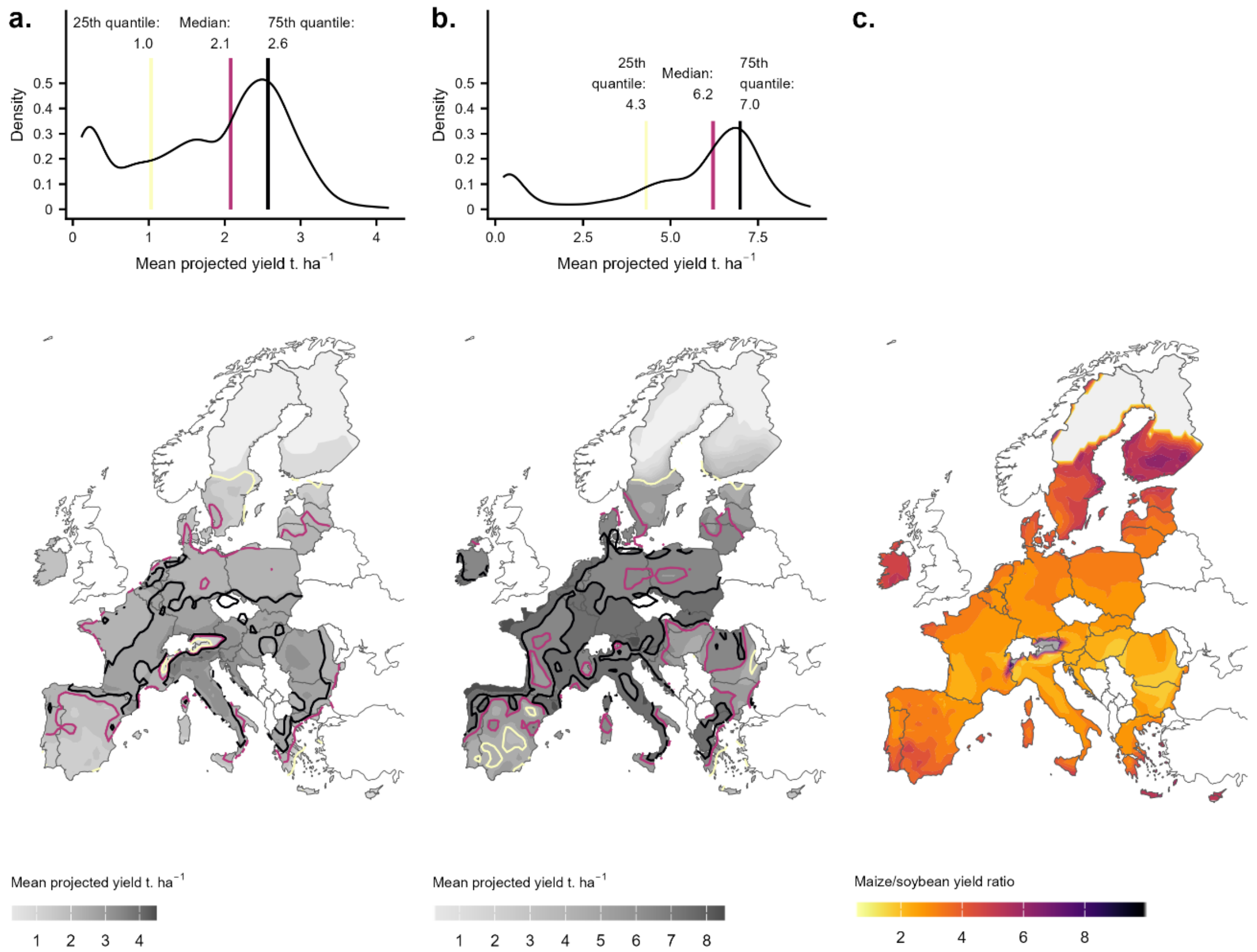

Figure 1. Soybean (a) and maize (b) rainfed yield projections (2000-2023 averages), and maize/soybean ratio (c) in the European Union. Projections were obtained from random forest models based on climate inputs (derived from the ERA5-land dataset) covering the crop growing seasons (April-November for soybean and April-December for maize) and irrigated fraction (estimated by SPAM2010, and set to zero for the yield projections). Climate inputs included monthly averages of minimum and maximum temperatures, total precipitation, solar radiation, reference evapotranspiration, and vapor pressure deficit. For each climate variable, the first two scores derived from a principal component analysis were used as climate predictors in the model. For panels (a) and (b), density graphics on the top show the variability in average projected yields, with 25th, 50th (median), and 75th quantiles indicated as vertical yellow, red, and black lines, respectively. Below, the maps represent the spatial distribution of projected average yields, with darker shades indicating regions with higher mean yields, whereas lighter shades representing lower yields (e.g., areas with average crop yield ~ 0 t.ha^{-1} are displayed in light grey). Regions corresponding to the 25th, 50th (median), and 75th yield quantiles are further delineated by yellow, red, and black contours, respectively. For panel (c), darker shades indicate high ratio between maize and soybean average projected yields, for example in the areas close to the Alps characterized by low soybean yields (i.e., <1 t.ha^{-1}) but moderate-to-high maize yields (i.e., >6 t.ha^{-1}).

1.15. On top, readers may want to know what crops are replaced. It may not be the case, as suggested in the abstract, that crop substitution is minimized. This would need to be shown?

Our response: We thank the reviewer for this comment.

Using the CROPGRIDS dataset (Tang et al., 2024), we examined the area of major crops grown in the EU (i.e, wheat, maize, barley, rapeseed, and sunflower) in regions with high soybean productivity. This analysis shows that in the areas where rainfed projected soybean reaches 1.0, 2.1, and 2.6 $t.ha^{-1}$ (corresponding to 25th, 50th, and 75th percentiles of soybean yield projections in the EU) wheat, maize, and barley are the major crops in terms of cultivated areas. Because maize and soybean share the same ecological niche (in particular, both are spring crops with similar growing season), we focus in the manuscript on maize. This is now mentioned in the revised manuscript.

However, many factors intervene in the choice to grow soybean at the expense of another crop. In the present study, we focus on the environmental suitability to grow soybean. The technical (i.e., adapted equipment), agronomic (e.g., cultivars), and economic (e.g., subsidies, market prices) aspects must also be taken into account to examine the variation in profitability that converting a given crop to soybean would induce. Further research is needed to examine the (dis)advantages in profitability for farmers when substituting these crops by soybean, considering various scenarios of agricultural subsidies and market prices.

The results of this analysis are presented in the new Supplementary Table 2 (both provided in the following pages).

Results (lines 99-102):

"The 25th, 50th (i.e., median), and 75th percentiles of averages soybean yield projections for 2000-2023 across the EU are 1.0, 2.1, and 2.6 $t.ha^{-1}$, respectively (Figure 1a, top panel). These levels of productivity are reached over areas of 103, 82 and 36 Mha, respectively (Figure 1a, bottom panel), where wheat, maize, and barley are the major cultivated crops (Supplementary Table 2)."

Reference: Tang, F.H.M., Nguyen, T.H., Conchedda, G. et al. CROPGRIDS: a global geo-referenced dataset of 173 crops. Sci Data 11, 413, doi:https://doi.org/10.1038/s41597-024-03247-7 (2024).

Supplementary Table 1. Area of major crops in regions with high suitability of soybean cultivation in the European Union.

	Crop area in 2020 (in ha) in the regions with projected soybean yield		
	**≥1.0 t.ha^{-1}**	**≥2.1 t.ha^{-1}**	**≥2.6 t.ha^{-1}**
**Wheat**	22.3	18.4	7.8
**Maize (grain and forage)**	11.8	11.0	6.5
**Barley**	10.5	6.7	2.7
**Rapeseed**	5.3	4.7	1.7
**Sunflower**	4.8	4.1	2.4

Notes: Data on crop area are taken from the CROPGRID gridded dataset (0.5°-resolution) (Tang et al., 2024). The soybean yield values of 1.0, 2.1, and 2.6 t.ha^{-1} correspond to the 25th, 50th (median), and 75th percentiles of the rained soybean yield projections in the EU from a random-forest model based on the two first principal components of climate variables and irrigation fraction.

1.16. 100 The -> A

Our response: Has been rephrased.

1.17. 102 largely -> greatly

Our response: Corrected (line 106).

1.18. 101-102 Production area of 82 million ha has median yield of 2.1 t/ha but current area is only 1 million ha. What does this tell us about the acceptability of a soybean yield of 2.1 t/ha and the associated gross margin? What do growers cultivate currently on the 81 million ha that they could use for soybean but use for another crop? Do they grow a more profitable crop? What other crop? If it is maize, there is really no problem, but if it is wheat, alfalfa, or grass, maybe there is a problem. Some contextualization is needed to make sense of the reported numbers.

Our response: See our response to comment 1.15.

1.19. 119 meet -> achieve

Our response: Corrected (line 131).

1.20. 121-123 How do you achieve that intercropping is primarily implemented in areas with high soybean yields? Do you do this by making a selection of suitable regions with high enough yields and distribute evenly, or do you do this by ranking pixels according to soybean yield and allocating pixels to soybean or MSI cultivation until the production target is met? This needs to be made clearer. (Actually, it is in M&M, but the reader needs to know ahead of time.)

Our response: In the present study, we assume that soybean (intercropped or sole cropped) would primarily be implemented in sites with the highest soybean productivity (estimated by multiplying soybean yield by the cropland area in each site), we sequentially allocate intercropping to highest yielding sites, ranked by projected soybean yields, until the considered level of self-sufficiency (i.e., 25%, 50%, 75%, or 100%) is achieved.

We added some elements from the Material and methods section to this paragraph to provide more details on the allocation process. In addition, the Figure 2 has been revised to make the allocation process clearer for the reader (see the new version of the Figure 2 below comment 1.58).

Result (lines 120-143):

“Following the procedure displayed in Figure 2 and detailed in the Supplementary Material 2, we examine the potential of maize-soybean intercropping to improve soybean self-sufficiency in the EU while maintaining maize production.

First, we combine our yield projections with the productive performances of intercropping to estimate the potential quantity of soybean produced across the EU by a reference maize-soybean intercropping system. To represent the current average productive performance of maize-soybean intercropping systems, we assume as a baseline that 1 ha of intercropping produces as much as 0.56 ha of soybean and 0.79 ha of maize grown separately (Xu et al., 2020), corresponding to pLER values of 0.56 and 0.79, respectively. We consider that intercropping is grown once every four years (Hartman et al., 2011; Pannecoucques et al., 2018), which means that the area cultivated under maize-soybean intercropping in a given site each year cannot exceed 25% of its cropland area.

We then estimate the area requirements under such a system to achieve 25%, 50%, 75%, and 100% soybean self-sufficiency in the EU (i.e., 9.1, 18.2, 27.2, and 36.3 Mt,

respectively), assuming that intercropping would primarily be implemented in regions with the highest soybean productivity. We sequentially allocate intercropping to the most productive sites, ranked by soybean production (i.e. estimated by multiplying projected sole soybean yield by the cropland area in each site), until the whole intercropping area produces a quantity of soybean covering the considered level of self-sufficiency (i.e., 25%, 50%, 75%, or 100%) or exceeds 25 Mha, corresponding to a quarter of cropland areas in the EU (Eurostats, 2024). We define this limit to avoid any extreme land use scenario leading to strong reductions of areas allocated to other crops, and because it is compatible with a soybean frequency of one-in-four year (Supplementary Table 2). Since intercropping involves that both soybean and maize are cultivated together in the same field and that maize's productivity remains above zero in the whole intercropping area (Figure 1), we also estimate the quantity of maize produced in this area."

References:

Eurostats. Farms and hectares by crops, utilised agricultural area, economic size of the farm and NUTS 2 region. https://doi.org/10.2908/EF_LUS_ALLCROPS (2024).

Hartman, G. L., West, E. D. & Herman, T. K. Crops that feed the World 2. Soybean—worldwide production, use, and constraints caused by pathogens and pests. Food Security 3, 5-17, doi:10.1007/s12571-010-0108-x (2011).

Pannecoucque, J. et al. Screening for soybean varieties suited to Belgian growing conditions based on maturity, yield components and resistance to Sclerotinia sclerotiorum and Rhizoctonia solani anastomosis group 2-2IIIB. The Journal of Agricultural Science 156, 342-349, doi:10.1017/S0021859618000333 (2018).

Xu, Z. *et al.* Intercropping maize and soybean increases efficiency of land and fertilizer nitrogen use; A meta-analysis. *Field Crops Research* **246**, 107661, doi:https://doi.org/10.1016/j.fcr.2019.107661 (2020).

1.21. 122 excluded from -> excluding

Our response: Corrected (line 430).

1.22. 124 "we assume" Suggest to add: "as a baseline"

Our response: Added (line 120).

1.23. 129 Add “arable” to “cropland”?

Our response: Added (line 138).

1.24. 130 Please include rationale why the cultivation of soybean and maize should be simultaneous, so readers know your reasoning.

Our response: The rationale is now explained in the revised manuscript.

> **Result (lines 141-143):**
>
> “Since intercropping involves that both soybean and maize are cultivated together in the same field and that maize’s productivity remains above zero in the whole intercropping area (Figure 1), we also estimate the quantity of maize produced in this area.”

1.25. 132-135 Self sufficiency for soybean in attained more easily if the crop is grown in areas in which it performs well. Is this done in all of the scenarios that follow? (See also above in line 121-123 and general comment no. 1).

Our response: Thank you. Please see our answer to the comment 1.20. and in the revised version of the manuscript (lines 120-143 and 426-440).

1.26. 142-143 Are the areas with higher soybean production prioritized in the allocation?

Our response: Following the same allocation procedure than intercropping, we assume that areas with higher soybean production are prioritized in the allocation. The allocation procedure has been clarified in the revised version of the manuscript (lines 120-143 and 426-440). See also our answer to the comment 1.20.

1.27. 156 “stronger competition” here means simultaneous intercropping. I would advocate using the term “simultaneous intercropping” to be clear and upfront. You may explain elsewhere that relay intercropping boost production by temporal complementarity, i.e. competition avoidance.

Our response: The text has been modified following the reviewer’s suggestion (line 173).

1.28. 157 a N fertilization -> an N fertilization (an because N is pronounced as “en”, so it starts with a vowel if pronounced. (also in 188)

Our response: We now use local estimates of N rates, the sentences were rephrased accordingly (lines 174 and 207).

1.29. 171 conventional -> conventionally managed. I suggest a change in wording because MSI is by no means “conventional”. It is an innovative system, but it can still be conventionally managed, i.e. with artificial fertilizer and biocides.

Our response: Changed (line 192).

1.30. 183 Suggest to write “assumed” before “LER values”.

Our response: Corrected (line 203).

1.31. 184-186 Please harmonize tense (mix of present and past tense)

Our response: The tense has been harmonized through the manuscript.

1.32. 185-186 I’d suggest omitting “regardless of intercropping’s frequency in crop sequences”. In any case, it is a bit implicit whether the reported area of soybean, maize and MSI are in each year, or they are areas in which these crops are cultivated, e.g., in 1:4 years.

Our response: Deleted (line 205).

1.33. 203-204 I don’t understand why pLERs of 0.30 and 0.31, respectively, are needed to achieve 25 and 50% soybean self-sufficiency. The difference in pLER is quite small, but the difference in self-sufficiency is large. I suppose that the self-sufficiency is mostly driven by the cultivation area of soybean or MSI. But why then are the minimum pLERs so similar? Please clarify.

Our response: Thank you for rising this point; you are right this result is mostly driven by the area allocated to each scenario. For example, 25% of soybean self-sufficiency in the EU is

met as soon as soybean pLER is higher than 0.3 on 12.2 Mha, while 50% is achieved with pLERs of 0.31 but assuming that 25 Mha is allocated to maize-soybean intercropping. The whole paragraph presenting these results has been rephrased.

> **Results (lines 232-241):**
>
> ‘For example, when the soybean pLER increases from 0.3 to 0.56, the area required to produce 9.1 Mt of soybean decreases from 12.2 to 6.3 Mha, respectively (panel c of Supplementary Figure 12), […]. Similarly, the level of 50% soybean self-sufficiency is reached as long as soybean pLER is higher than 0.31, assuming that 25 Mha is allocated to maize-soybean intercropping (panels a and c of Supplementary Figure 13). »

1.34. 208 Why does maize self-sufficiency not exceed 75% when soybean pLER exceeds 0.41? I would think that maize production and maize self-sufficiency are altogether independent from soybean pLER. I am missing something in the reasoning. If there is insufficient maize production in MSI, then the remainder of maize can be produced in sole crops? This is related to what is replaced by MSI? Is it maize? Is it another crop?

Our response: We agree with the reviewer that when both pLERs values increase, the total productivity of maize-soybean intercropping increases as well. However, intercropping becomes more efficient for soybean production as soybean pLER increases and requires less land to meet the self-sufficiency target fixed, thus reducing the production of companion maize (because the area allocated to intercropping decreases). This is why the % of maize self-sufficiency attainable by maize-soybean intercropping decreases when soybean pLER increases.

We have rephrased the paragraph to explain more clearly this trade-off.

> **Results (lines 221-238):**
>
> “Sensitivity analyses investigating the effect of variations in pLERs values for soybean (from 0.3 to 0.7, by 0.01-increment) and maize (from 0.5 to 0.9, by 0.01-increment) show a trade-off between the pLER values and the total allocated surface: as intercropping becomes more efficient for soybean production (i.e., as soybean pLER increases), less land is required to meet the soybean self-sufficiency target, thus reducing the production of companion maize (because the area allocated to intercropping decreases). To illustrate this, Supplementary Figure 12 shows the results of these sensitivity analyses when targeting 25% of soybean self-sufficiency (i.e., 9.1 Mt of soybean) in the EU. This level of soybean self-sufficiency is met as soon as soybean pLER is higher than 0.3 (panel a of

Supplementary Figure 12). Consequently, maize production and intercropping area decrease as the value of soybean pLER increases, and this area reduction leads to a decrease of the total maize produced by the intercrops if the maize pLER remains unchanged. For example, when the soybean pLER increases from 0.3 to 0.56, the area required to produce 9.1 Mt of soybean decreases from 12.2 to 6.3 Mha, respectively (panel c of Supplementary Figure 12), reducing the companion maize production from 58.9 to 28.8 Mt when assuming a fixed value of maize pLER of 0.79. Increasing the maize pLER mitigates the decrease in the amount of maize produced by intercropping. When fixing the soybean pLER at 0.3, the minimum maize pLER values required to reach 50% and 75% of maize self-sufficiency levels are 0.58 and 0.86, respectively (panel d of Supplementary Figure 12)."

1.35. 215 A pLER of 0.46 for a legume is not low. See, e.g., Yu et al. (2015, 2016) and Li et al. (2020).

Li CJ, Hoffland E, Kuyper Th W, Yu Y, Zhang CC, Li HG, Zhang FS, van der Werf W (2020) Syndromes of production impact yield gains in intercropping. Nature Plants 6, 653–660. http://dx.doi.org/10.1038/s41477-020-0680-9

Yu Y, Stomph TJ, Makowski D, van der Werf W (2015) Temporal niche differentiation increases the land equivalent ratio of annual intercrops: a meta-analysis. Field Crops Research 184, 133–144. http://dx.doi.org/10.1016/j.fcr.2015.09.010

Yu Y, Stomph TJ, Makowski D, Zhang L, van der Werf W (2016) A meta-analysis of relative crop yields in cereal/legume mixtures suggests options for management. Field Crops Research 198: 269-279. http://dx.doi.org/10.1016/j.fcr.2016.08.001

Our response: The sentence was modified following reviewer's comment.

Results (line 247-250):

"Within the constraint of 25 Mha (i.e., 25% of European cropland) of maize-soybean intercropping, a soybean pLER of 0.46 is required to meet three-quarters of the EU's demand in soybean, while achieving full soybean self-sufficiency would require a minimum pLER of 0.62."

1.36. 216 "25 million hectares" Suggest to add: "of maize soybean intercropping"

Our response: The sentence was clarified. See also our response to comment 1.35 above.

In the discussion

1.37. 238 Add here “growing the intercrops in areas that are the most suited for soybean cultivation across Europe”?

Our response: Thank you for this suggestion. We modified the sentence accordingly.

Discussion (lines 268-271):

“Our results suggest that growing maize-soybean intercropping in areas most suited for soybean cultivation has the potential to enhance EU soybean and maize self-sufficiency, thus substantially reducing Europe’s dependency to imported supplies.”

1.39. 250-253 “If more productive intercropping scenarios […] in the least productive intercropping scenario”. I suppose you are only talking about soybean pLER here. Then I would refrain from calling it “productivity”. LER is not productivity. It is relative performance.

Our response: Thank you for this suggestion. We modified the sentence accordingly.

Discussion (lines 283-286):

“While intercropping scenarios characterized by pLERs values >0.5 for soybean and > 0.7 for maize lead to higher improvements in self-sufficiency and land savings, our results suggest that intercropping maize and soybean in the EU remains an interesting option to improve self-sufficiency compared to sole crops even with lower pLERs.”

1.40. 255 See previous comment: better call it simultaneous intercropping

Our response: The sentence was improved following the suggestion of the reviewer.

Discussion (lines 286-289):

“In this study, intercropping’s contribution to maize and soybean self-sufficiency slightly decreased with higher N inputs or when maize and soybean were grown during the same period, but still strongly improved self-sufficiency compared to the current EU level.”

1.41. 266 condition -> conditions

Our response: Corrected (line 290).

1.42. 275 Omit "only"

Our response: Removed (line 309).

1.43. 310-312 What is replaced?

Our response: See our response to comments 1.15.

Related to the material and methods

1.44. 338 producing -> production

Our response: Replaced (line 386).

1.45. 338 How was detrending done? You mean time trend?

Our response: In our study, yield data for each site was expressed relatively to the expected value at the most recent year of available data (i.e., 2016). This is now mentioned in the revised manuscript and in the Supplementary Material 3 (section 1).

Material and methods (lines 378-383):

"For each site, we expressed annual yield data relatively to the expected value at the most recent year of available (i.e., 2016). This detrending procedure allowed to remove the systematic temporal component of yield that is attributed to technological progress (e.g., genetic improvement, management, inputs) and to focus on the interannual variability around that trend, which is typically interpreted as being driven by climate, weather, soil, or other non-progress-related factors."

Supplementary Material 3:

"To avoid any confusion with technological progress due to improved cultivars and technological progress, yield data were detrended. For a given site and a given crop, yield

time series are used to fit a cubic smoothing spline f(t) (3, 4). Yield for each year t of this site is then expressed as:

$$Detrended\ yield(t) = f(tmax) + A(t)$$

with f(tmax), defined as the expected yield value at the most recent year of available data for this site (generally 2015 or 2016), and A(t), the yield anomaly, calculated as the difference between expected yield f(t) and actual yield in year t. This allowed to remove the systematic temporal component of yield that is attributed to technological progress (e.g., genetic improvement, management, inputs). What remains is the interannual variability around that trend, which is typically interpreted as being driven by climate, weather, soil, or other non-progress-related factors.

1.46. 339 What is the meaning of a detrended “mean yield”? Conceptually, which year is represented by the detrended mean yield; e.g. the yield in 1980, in 2030? Is it a future year or a year in the past?

Our response: Thank you for this question. Here, because yields are expressed relatively to the expected value in the most recent year (2016), all detrended values are normalized to the 2016 technology level. Therefore, the detrended mean yield is the average of yield values, rescaled to the technological baseline of 2016, and independent of the original calendar years of observation. This clarification is now provided in the manuscript.

Material and methods (lines 390-392):

“Mean (standard deviation) yield, representing the average (standard deviation) of values rescaled to the technological baseline of 2016 and independent of the original calendar years of observation, was 2.6 $t.ha^{-1}$ (1.1 $t.ha^{-1}$) for soybean and 9.5 $t.ha^{-1}$ (3.8 $t.ha^{-1}$) for maize.”

1.47. 352 Are these predictions of soybean yield for water limited rainfed conditions or irrigated conditions?

Our response: These predictions were obtained for rainfed conditions. This is now specified in the manuscript (line 401).

1.48. 353 for each crop -> for both crops (to leave no doubt that there are just two species involved)

Our response: Corrected (line 402).

1.49. 376 This is fine; but I am still a bit confused whether 25 Mha of a crop at EU level means that EACH YEAR, there is 25 Mha of this crop, or there is Mha of land in which this crop occurs in a 1:4 rotation, so 6.25 Mha in each year. I presume the former, but I am not 100% it is not the latter. Please state it.

Our response: In this sentence, the coefficient of 25% characterizes the return frequency of intercropping *in each site*, allowing the surface allocated to vary according to the cropland available in each site. It is different from the limit of 25% of cropland totally allocated to intercropping *at the scale of the EU*, which corresponds to a 25 Mha area.

We revised the manuscript in order to ease the distinction between both.

Material and methods (lines 423-425):

"We set this coefficient at 1/4, […] which is equivalent to allocating each year 25% of the cropland area *in each site*."

Material and methods (lines 432-435):

'To limit cropland expansion and competition with other crops, total area allocated to intercropping *at the scale of the EU* should not exceed 25 Mha, equivalent to 25% of EU cropland in 2018 (Eurostats, 2024a) or to the area devoted to wheat, the most widely cultivated crop in the EU over 2018–2022 (Eurostats, 2024b)."

References:

Eurostats. Crop production in EU standard humidity. https://doi.org/10.2908/APRO_CPSH1 (2024a).

Eurostats. Farms and hectares by crops, utilised agricultural area, economic size of the farm and NUTS 2 region. https://doi.org/10.2908/EF_LUS_ALLCROPS (2024b).

1.50. 381-398 This section is fairly clear, but I had to read several times to be sure I understood. I think readers need the understanding conveyed by this paragraph when they read the results (which are BEFORE the materials and methods). I suppose that more

methods need to be infused into the results to make readers (and this reviewer) understand the methods when reading the results.

Our response: Thank you for this comment. Following the reviewer's comment, we added more details on the Methodology implemented in the study in the Results section. The text has been improved (lines 413-452) and Figure 2 has been modified to make it clearer to the reader. The reviewer can also refer to our response to comment 1.10.

1.51. 381-398 Why does the area of MSI need to be capped at 25 Mha? Is this because this represents 25% of the whole EU arable crop area, and soybean cannot be cultivated at greater frequency than 1:4? Please, if so, state it.

Our response: The total area allocated to maize-soybean intercropping *at the scale of the EU* was bounded at 25 Mha in order to limit cropland expansion and competition with other crops. The total area allocated should not exceed this threshold, which is equivalent to 25% of EU cropland in 2018 or to the area devoted to wheat, the most widely cultivated crop in the EU over 2018–2022. The reviewer can also see our see our response to comment 1.49.

1.52. 381-398 I wonder whether adopting MSI would lower the maize cultivation frequency on parcels in maize growing areas. If so, this could boost maize yields by mitigating soil-borne diseases. Furthermore, incorporation of soybean in rotations benefits productivity of cereals (e.g. Xia et al., 2023, but there are many other sources)

> Xia HY, Qiao YT, Li XJ, Xue YH, Wang N, Yan W, Xue YF, Cui ZL, van der Werf W (2023) Moderation of nitrogen input and integration of legumes via intercropping enable sustainable intensification of wheat-maize double cropping in the North China Plain: A four-year rotation study. Agricultural Systems 204: 103540. https://doi.org/10.1016/j.agsy.2022.103540

Our response: We thank the reviewer for rising this point. Indeed, we agree that adopting maize-soybean intercropping would lead to changes in crop sequences. These changes would likely involve a decrease in the temporal frequency of some crops, like maize, and an increase in the temporal frequency of other crops like soybean. It is well known that temporal crop diversity in rotations has many advantages in terms of pests, diseases and weeds control, nitrogen management especially for legume crops, with positive effects on crop yields (Mudare et al. 2025, Zhao et al. 2022). Estimating the crop sequence effect of maize-soybean intercropping would be very interesting, but despite few studies (Xia et al. 2023)

data are currently lacking for doing so in a robust way. However, we modified the text to highlight this as a point deserving further research.

Discussion, lines 321-328:

"Additionally, adopting maize–soybean intercropping would alter crop sequences, likely reducing the temporal frequency of some crops (e.g. maize) while increasing that of others (e.g. soybean). Such increases in temporal crop diversity within rotations offer multiple agronomic benefits, including improved control of pests, diseases, and weeds, as well as enhanced nitrogen management (particularly when including legume crops) with positive effects on crop yields (Mudare et al., 2025; Zhao et al., 2022). However, quantitative evidence on the effects of crop sequences that include maize–soybean intercropping remains limited. Although a few studies exist (e.g. Xia et al., 2023), the available data are currently insufficient to support robust assessments."

References:

Mudare, S., Jing, J., Makowski, D., He, X., Liang, Z., Sims, Z., ... & Cong, W. F. (2025). Crop rotations synergize yield, nutrition, and revenue: a meta-analysis. Nature communications, 16(1), 9552.

Xia HY, Qiao YT, Li XJ, Xue YH, Wang N, Yan W, Xue YF, Cui ZL, van der Werf W (2023) Moderation of nitrogen input and integration of legumes via intercropping enable sustainable intensification of wheat-maize double cropping in the North China Plain: A four-year rotation study. Agricultural Systems 204: 103540. https://doi.org/10.1016/j.agsy.2022.103540

Zhao, J., Chen, J., Beillouin, D., Lambers, H., Yang, Y., Smith, P., ... & Zang, H. (2022). Global systematic review with meta-analysis reveals yield advantage of legume-based rotations and its drivers. Nature Communications, 13(1), 4926.

1.53. 388-389 You mention the area of wheat but do not state what the area is. Is it 25 Mha? Please clarify. Is it necessary to consider the replacement of wheat area by MSI area, with consequences for wheat self-sufficiency? Does it need quantification or discussion?

Our response: The area of 25 Mha corresponds to 25% of the European cropland but also to the area devoted to wheat in the EU. We used this as a threshold in our allocation procedure in order to limit the competition with other crops. This is now explained in the revised version of the manuscript.

Material and methods (lines 428-435):

"Our allocation process was guided by three assumptions: […] (3) to limit cropland expansion and competition with other crops, total area allocated to intercropping *at the scale of the EU* should not exceed 25 Mha, equivalent to 25% of EU cropland in 2018 (Eurostats, 2024a) or to the area devoted to wheat, the most widely cultivated crop in the EU over 2018–2022 (Eurostats, 2024b)."

References:

Eurostats. Crop production in EU standard humidity. https://doi.org/10.2908/APRO_CPSH1 (2024a).

Eurostats. Farms and hectares by crops, utilised agricultural area, economic size of the farm and NUTS 2 region. https://doi.org/10.2908/EF_LUS_ALLCROPS (2024b).

1.54. 416 Please make clear this is about relay intercropping.

Our response: This is now clarified.

Material and methods (lines 474-479):

"The results of Xu et al., (2020) show that soybean pLER vary according to fertilization practices, especially nitrogen (N) inputs, but also depending on the overlap between growing periods of maize and soybean. This overlap is often characterized by the temporal niche differentiation (TND). High TND values (i.e., close to 1) correspond to small overlap, where the second species is sown just before the harvest of the first crop (e.g., relay intercropping), whereas a TND of zero represents systems in which both species are sown and harvested simultaneously."

Reference: Xu, Z. et al. Intercropping maize and soybean increases efficiency of land and fertilizer nitrogen use; A meta-analysis. Field Crops Research 246, 107661, doi:https://doi.org/10.1016/j.fcr.2019.107661 (2020).

Related to the references

1.55. 518 Incomplete reference.

Our response: Completed.

Related to the figures

1.56. Figure 1 Colour scale of panels a and b is counter-intuitive, with dark blue (cold) representing the highest yields and yellow (somewhat warm) representing the lowest yield. Please consider using a different colour scale that is more intuitive. In panel c it is unclear whether the ratio shown is maize/soybean yield or soybean/maize yield.

Our response: The Figure has been modified according to reviewer's comments 1.56, to make it clearer for the reader. See also our response to the comment 1.14.

1.57. Sentence in lines 593-596 does not run.

Our response: The caption of Figure 1 has been revised (lines 465-479).

1.58. Figure 2 This figure did not help me as a reader. It might help if the caption would explain the procedure and all symbols used, but in the current situation, the reader needs to figure out the meaning by himself. Suggest to move to supplementary or make it totally self explanatory.

Our response: Following reviewers' comments (see also comments 2.6 and 2.7), we have improved the Figure 2 in order to explicit all symbols and notations to the reader to make it self-explanatory. We also added information on the allocation procedure and the different steps of our study. See the new version of this Figure in the following page.

[Figure Redacted]

Figure 2. Procedure applied to estimate the area requirements under intercropping (Int) and sole cropping (SC) to meet a target soybean production (P_S^*) in the European Union (EU), to compare the efficiency of both strategies in maximizing both soybean and maize coproduction, and to quantify the land saving allowed by the most efficient strategy at the scale of the EU.

1.59. Figure 3 Please enlarge the maps. They tell an important story about the allocation but they are too small.

Our response: We thank the reviewer for this suggestion. The Figure 3 has been modified to give more space to the maps.

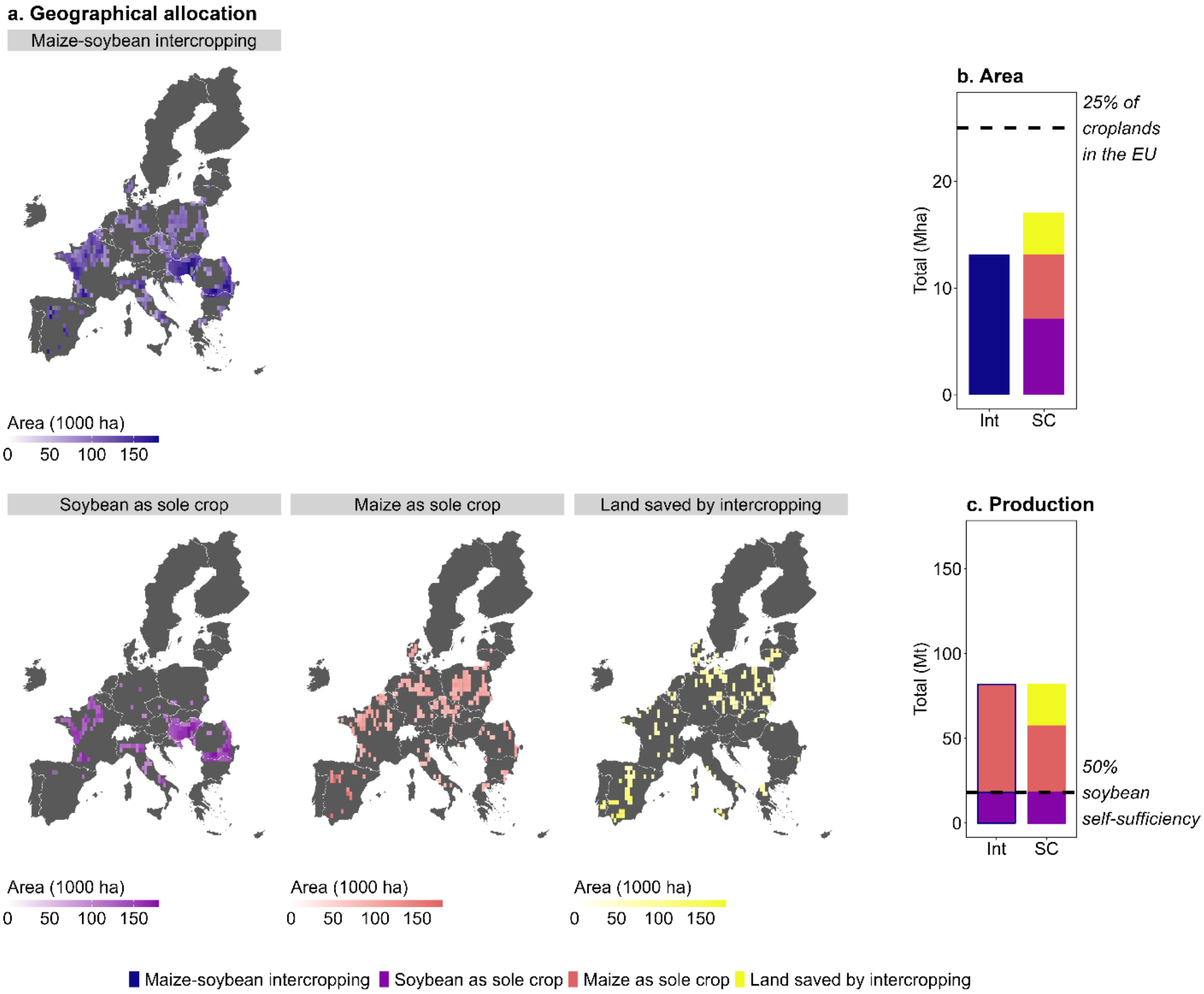

Figure 3. Comparison of maize-soybean intercropping (Int) and sole cropping (SC) for 50% soybean self-sufficiency in the European Union (EU). Maize-soybean intercropping was sequentially allocated in highest yielding sites, ranked by soybean productivity based on mean average production over 2000-2023 period in rainfed conditions, until 50% soybean self-sufficiency (i.e., 18.2 Mt) is reached. Sole soybean was sequentially allocated in the highest yielding sites until reaching 50% soybean self-sufficiency, then sole maize was allocated to the remaining sites so that the total covered area is equal in both strategies. Panel (a) displays the geographical allocation of crops with the Int. (top map) and with the SC (bottom maps) strategies. In the SC strategy, the two first maps represent the allocation of sole soybean and sole maize, respectively, while the third map shows the additional area required to produce the same coproduction than in Int. Panels (b) and (c) show the corresponding area requirements and maize-soybean co-production, respectively. All simulations are based on (i) yield projections from a random-forest model based on the two first principal components of climate variables and irrigation fraction (set to zero here), (ii) crop return frequency of one year in four, and (iii) partial land equivalent ratios equal to 0.56 and 0.79 for soybean and maize, respectively (Xu et al., 2020). The maximum total allocated area was set at 25% of croplands in

EU, indicated as dotted line in the panel (b). Self-sufficiency levels of 50% corresponds to soybean, indicated as horizontal dotted line in the panel (c).

Reviewer #3

Remarks to the Author

2.1. I thank the authors for their efforts in revising the manuscript. Many aspects have been improved.

Our response: We thank the reviewer for this positive comment.

2.2. However, my main concern regarding the static ratio LER = 0.56 + 0.79 remains unresolved.

I acknowledge the authors' argument that yield variability of the two crops and the allocation model may introduce some variation. However, the allocation model itself is relatively simple and based on many assumptions, which may not be sufficient to justify the conclusions.

Our response: The allocation model was designed based on several assumptions about crop productivity (i.e., climate conditions and water management) and intercropping system features (i.e., relative performance of each crop in the mix represented by the partial land equivalent ratio, return frequency in the crop sequences). To avoid too much competitions with other crops, we also constrained the area occupied by intercropping to 25% of the European cropland. We decided to present few main analyses but explore in details the robustness of the results through several sensitivity analyses, which showed that the main patterns and conclusions are robust across a wide range of parameter values and assumptions.

We have clarified the Results and Methods sections in the revised manuscript to ensure that the conclusions are interpreted accordingly. The reviewer can see our response the comments 1.10, 1.20, 1.25, and 1.26 above, which explain how we improved the manuscript in order to clarify the assumptions of our allocation procedure. The reviewer can also see the new version of Figures 2 and 3, also provided above this comment.

2.3. More importantly, the central focus of the manuscript is intercropping. The efficiency or land-saving benefit of intercropping is measured by the land equivalent ratio (LER):

LER = f(weather, crop allocation, management) where management includes nitrogen fertilization, irrigation regime, and relay vs. simultaneous planting. In other words, LER is not a fixed value but a dynamic outcome of these interacting factors. While the ML yield model

developed by the authors successfully captures weather effects, it does not explicitly address LER, which is the primary driver of land-use efficiency and land saving.

Our response: We thank the reviewer for this comment about the ML model. Regarding the variability of (p)LERs, we examined whether our estimates were robust in scenarios with pLER varying values of soybean (0.3 to 0.7, by 0.01-increment) and maize (0.5 to 0.9, by 0.01-increment) to increase the range of possible performances of intercropping over sole cropping (Methods section, lines 468-473, results presented in Supplementary Figures 12-15 and in the text lines 221-250). In other sets of sensitivity analysis, we include the factors that influence the most the performance of pLERs (i.e., N fertilizer, and TND). In particular, we computed site-specific pLERs values taking into account the spatial variability of N fertilizer use. These sensitivity analyses make us confident about our overall results at the European scale (i.e., on the strong benefices of intercropping vs sole cropping for improving maize and soybean self-sufficiency while reducing the area required).

We acknowledge that substantial variability might occur at the local scale. Local decisions about intercropping should be based on local data produced to regional institutes, and this is out of the scope of our study. We are aware that some of the important factors are not included in our sensitivity analyses (i.e., relative density, environmental conditions) (Ruillé et al., 2026). However, comprehensive datasets to model pLERs according to these factors are currently not available to the best of our knowledge. This is acknowledged in the discussion section of the manuscript (lines 301-305 and 326-328).

More details on this analysis are provided in the reviewer's comment 1.10 above and comment 2.4 below. The reviewer can also see our response to 2.5 below, regarding the development of a model to predict (p)LERs.

2.4. In addition, the authors applied a fixed nitrogen rate of 148 kg N ha^{-1} to calculate LER using the regression equation from Xu et al. This approach neglects spatial variability in nitrogen management and, therefore, does not provide a solid basis for conclusions about land saving.

Our response: We acknowledge that the analysis initially included to integrate the effect of N fertilization on maize and soybean pLERs did not take into account the spatial variability of fertilization practices. Following the reviewer's comment, we performed a more robust sensitivity analysis based the NPKGRIDS gridded dataset (Nguyen et al., 2024). This dataset provided data on N fertilizer application rates of 2020 for each site in the European Union, allowing us to compute site-specific soybean and maize pLERs. To prevent us to

overestimate the potential of intercropping in Europe, we considered that the applied N fertilizer rates were those of maize, which receive higher N amounts than soybean.

This new sensitivity analysis is now detailed in the revised manuscript. See also the new Supplementary Figure 23 showing the applied rates, as well as derived pLER values for each crop (also provided in previous pages of this Response Letter).

Results (lines 174-179):

"Assuming N fertilization rates equivalent to those of maize in 2020 in the EU (Nguyen et al., 2024), pLERs values varied from 0.45 and 0.59 for soybean and 0.77 and 0.79 for maize across the EU (Xu et al., 2020), respectively (Supplementary Figure 23). In this case, intercropping uses 19% and 21% less land compared to sole cropping to achieve similar co-production of both crops considering the targets of 25% and 50% soybean self-sufficiency in the EU (Supplementary Table 4)."

Results (lines 206-211):

"Similar levels of self-sufficiency are achieved after incorporating the effects of N inputs and TND on the pLERs. In the analysis which incorporates the effect of N fertilization rates, the potential self-sufficiency rates range from 49 to 90% for soybean and 83 to 143% for maize, respectively, depending on the return frequency. Similarly, 49-90% and 80-138% self-sufficiency for soybean and maize, respectively, could be achieved in a scenario characterized by a TND of zero (i.e., corresponding pLERs values: 0.53 and 0.76), respectively (Supplementary Figure 11)."

Material and methods (lines 483-488):

"To take into account the spatial variability in N management in the EU, we computed site-specific soybean and maize pLERs according to local N fertilizer application rates of 2020 provided by the NPKGRIDS gridded dataset (Nguyen et al., 2024) using published regression model (Xu et al., 2020). To compute less optimistic pLER values (i.e. lower) assuming high N fertilizer doses, we considered that the applied N fertilizer rates were those of maize, which receive higher amounts of N. Supplementary Figure 23 shows the applied rates, as well as derived pLER values."

References:

Nguyen, T.H., Tang, F.H.M., Conchedda, G. et al. NPKGRIDS: a global georeferenced dataset of N, P2O5, and K2O fertilizer application rates for 173 crops. Sci Data 11, 1179 (2024). https://doi.org/10.1038/s41597-024-04030-4

Xu, Z. et al. Intercropping maize and soybean increases efficiency of land and fertilizer nitrogen use; A meta-analysis. Field Crops Research 246, 107661, doi:https://doi.org/10.1016/j.fcr.2019.107661 (2020).

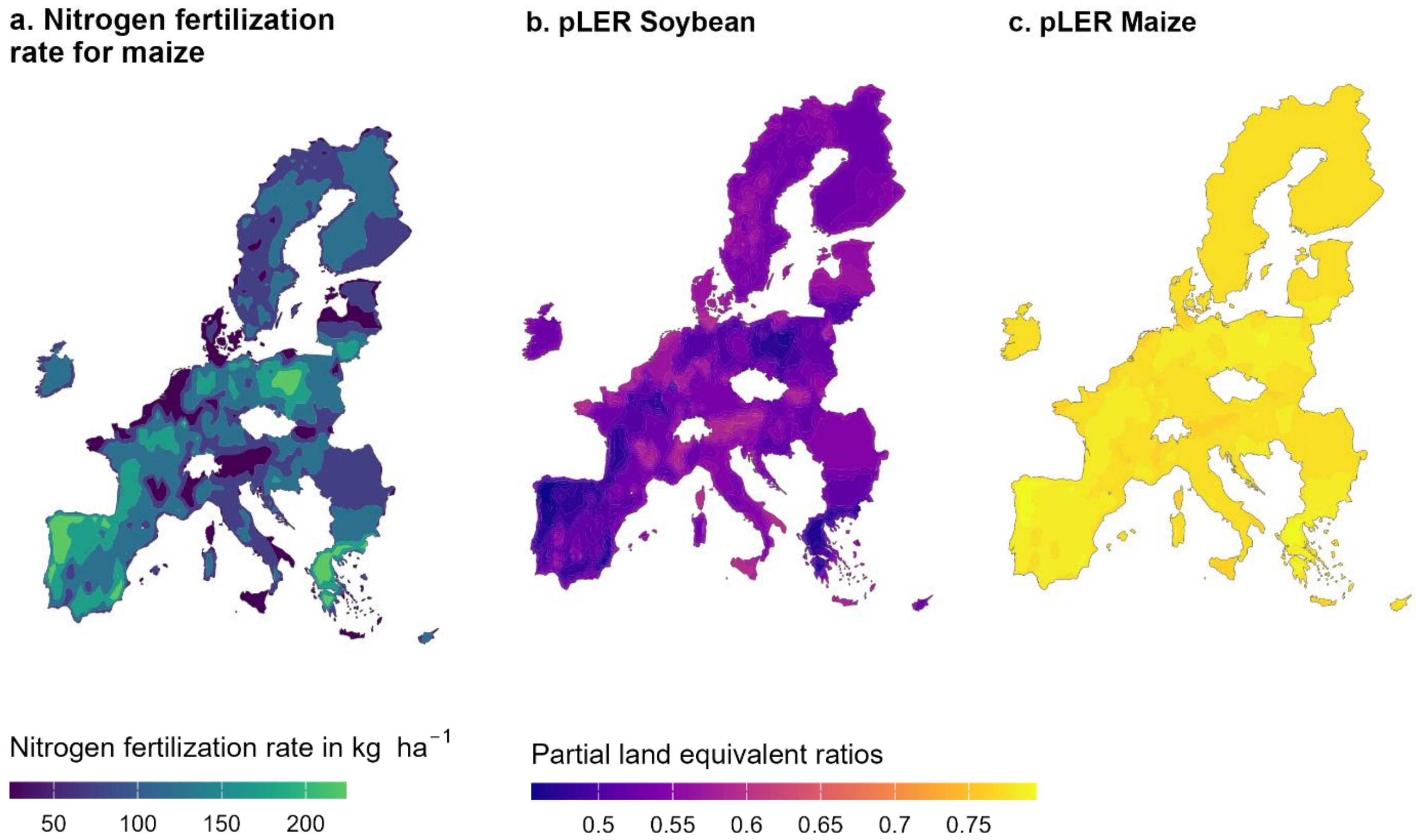

Supplementary Figure 10. The performances of maize-soybean intercropping according to (a) nitrogen fertilization rates for maize of 2020, represented by the partial land equivalent ratios (pLERs) of (b) soybean and (c) maize. Gridded fertilization rates (0.5°-resolution) were obtained from the dataset of Nguyen et al. (2024). Soybean and maize pLERs values were computed based on the equations of Xu et al. (2020)**.**

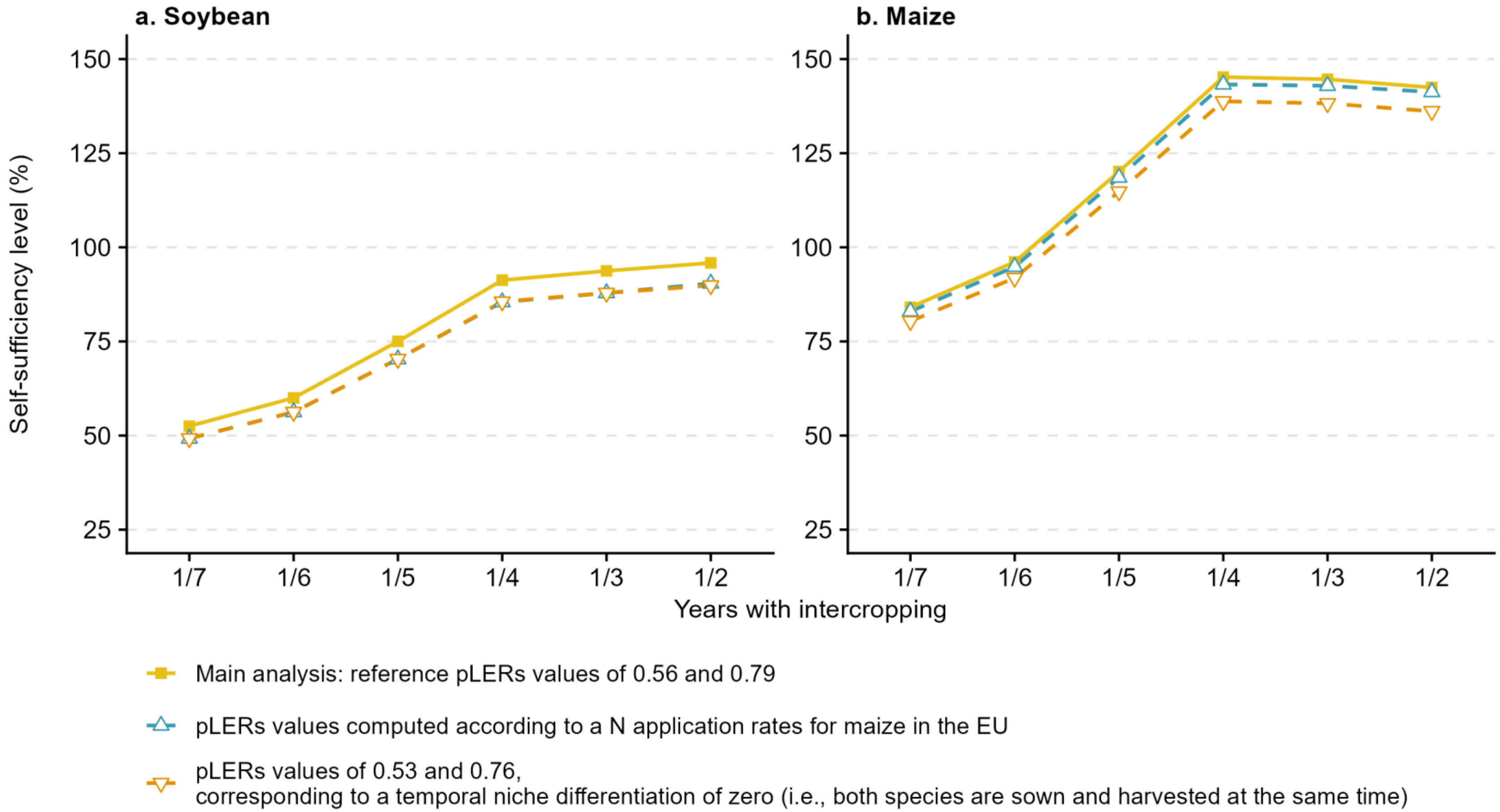

Supplementary Figure 11. Levels of soybean (a) and maize (b) self-sufficiency in the European Union (EU) achieved from intercropping for different assumptions of partial land equivalent ratios (pLERs) and crop return frequency.
Crop frequencies of 1/7, 1/6, 1/5, 1/4, 1/3, and 1/2 correspond to allocating maize-soybean intercropping on 14%, 16%, 20%, 25%, 33%, or 50% of cropland area in each grid-cell. Intercropping was first allocated to soybean highest-yielding grid-cells, and in all cases total intercropping area was constrained to not exceed 25% of croplands in the EU (i.e., 25 Mha). Squares represent the results obtained with pLER values of 0.56 for soybean and 0.79 for maize, corresponding to the average productive performances of maize-soybean intercropping systems estimated in a previous meta-analysis (Xu et al., 2020). The facing down triangles represent the results obtained with site-specific pLER values recomputed according to local N fertilization rate of maize in the European-Union (data from the NPKGRIDS dataset [Nguyen et al., 2024]), based on the results of Xu et al. (2020). Facing upwards triangles represent the results obtained with pLER values corresponding to a temporal differentiation niche of 0 (i.e., 0.53 for soybean and 0.76 for maize), based on the results of Xu et al. [2020]).

Supplementary Table 2. **Soybean and maize coproduction and surface requirement for 25, 50, 75, and 100% soybean self-sufficiency in the European Union (EU) achieved by intercropping or sole cropping *according to local nitrogen fertilization rates in the EU*.**

Target % of EU's self-sufficiency in soybean [a]	Intercropping		Sole crops			Total		Land-saving [b]
	Soybean	Maize	Soybean	Maize	Additional land required	Intercrop-ping	Sole crop	
**Production (Mt)**								
**25%**	9.1	30.1	9.1	19.8	10.3	39.2	39.2	
**50%**	18.2	67.3	18.2	44.7	22.6	85.5	85.5	
**75%**	27.2	105.2	27.3	68.7	26.8	132.3	122.8	
**100%**	31.0*	121.9	36.3	65.0	5.4	152.9	106.7	
**Surface (Mha)**								
**25%**	6.6		3.4	3.2	1.5	6.6	8.1	-19%
**50%**	13.9		7.1	6.8	3.7	13.9	17.6	-21%
**75%**	21.6		10.9	10.7	4.2	21.6	25.9	*-17%***
**100%**	25.0		14.8	10.2	0.9	25.0	25.9	*-3%***

Notes: Crop return frequency was one-in-four year for all simulations, and total allocated area was limited to 25% of croplands in the EU (i.e., 25 Mha). For intercropping, partial land equivalent ratios were computed according to local nitrogen fertilization rates from the NPKGRID gridded dataset (Nguyen et al., 2024) based on the equation derived by Xu et al. (2020). The computed values varied between 0.45 and 0.59 for soybean and between 0.77 and 0.79 for maize, respectively.

[a] Self-sufficiency levels of 50, 75, and 100% correspond to 18.2, 27.2, 36.3 Mt soybean.

[b] Computed as the percentage of surface saved by intercropping compared to sole crop strategy.

* Target production not reached even when soybean was grown of 25% cropland in the EU.

** Production from intercropping strategy higher than sole crops.

2.5. To strengthen the manuscript, I recommend that the authors consider constructing a machine learning model directly for LER and investigate how interactions among weather, management, and allocation factors could be extrapolated from other regions to Europe. Such an approach would provide more robust and generalizable evidence of the land-saving potential of intercropping under diverse conditions.

Our response: We thank the reviewer for this suggestion. Developing such a model would indeed require experimental datasets that explicitly explore variations in LER as a function of crop management practices and weather conditions. To the best of our knowledge, such comprehensive datasets are currently not available.

We nevertheless fully agree with the reviewer on the scientific relevance of this approach. In this perspective, the establishment of a shared, standardized, and open-access global database on intercropping systems—including detailed information on management, environmental conditions, and LER outcomes—would represent a major advance for the community and would enable the type of modeling effort suggested by the reviewer in future studies.

2.6. Another major concern is the allocation of intercropping, sole soybean, and sole maize. Based on the current description of the methods, it is difficult to reconcile the results presented in Figure 1 with those in Figure 3. Greater methodological clarity is needed.

Our response: Following reviewers' comments (see also comment 1.58), we have improved the Figure 2 in order to better explain the allocation procedure and the different steps of our study. See also our answer to comment 2.8 below.

Specific comments

2.7. Figure 2: A clear legend or explanation for P^i_m and P^m_m is still missing.

Our response: We thank the reviewer for rising this issue. The Figure 2 and its caption were improved to explicitly explain each term. Please see the new version of Figure 2 in the revised manuscript, as well as in previous comment 1.58 above.

2.8. Figure 3: Why does southeastern France show no intercropping or soybean cultivation, despite Figure 1 indicating higher yields than in northwestern France?

Our response: We thank the reviewer for this question. The panels a and b of Figure 1 show the estimated yield projections of soybean and maize, respectively, while the Figure 3a show the allocation of maize-soybean intercropping and sole soybean crop, which are preferentially allocated to the area which produce the most soybean. The soybean production is estimated by multiplying the estimated yield by the cropland area in each grid cells and by the pLER for intercropping allocation. The production of soybean in the North of France was higher than in the South of France (mainly due to higher cropland area per site), which explained the differences between the two maps. The allocation procedure has been clarified in the Results section (lines 120-143) and in the new version of Figure 2 as well. In addition, the caption of Figure 3 now explicitly explains what are the different maps (see our response to comment 2.9).

2.9. Figure 3: Inconsistencies remain between the intercropping and land-saved maps.

Our response: The intercropping map shows the area where intercropping is preferentially allocated to produce enough soybean to meet 50% self-sufficiency in the EU (18.2 Mt). This allocation is associated to some given quantities of soybean and maize. As specified in the revised manuscript, it coincides with the area allocated to sole soybean (second map) and to the remaining area allocated to sole maize (third map). The land saving map shows the additional area required by the sole maize strategy to produce the same quantity of maize than in intercropping. This is why these two maps do not cover the same areas. These additional lands are mainly located in Spain and in the Eastern EU, as now mentioned in the revised manuscript (line 166). The caption of Figure 3 has been completed to make this clearer to the reader.

Remarks on code availability

2.10. The code associated with this manuscript has not yet been made publicly available. In the response to reviewers, the authors provided a different link, which does not directly correspond to the present manuscript.

Our response: We thank the reviewer for rising this issue. We acknowledge that the link provided for the alternative repository mentioned in the previous response letter was associated with another study (Chen et al., 2024). The code associated with the present study has been made publicly available for the review process in the repository mentioned in the Material and Methods section of the manuscript (https://github.com/MathildeChen/Maize_Soybean_Intercropping_Europe).

Manuscript reference: NCOMMS-25-06543-C entitled “High potential contribution of intercropping to soybean and maize self-sufficiency in Europe”

RESPONSE LETTER TO REVIEWER COMMENTS

April 24th 2026

Dear reviewers,

We thank you for your constructive comments on the manuscript.

Please find below the response to each of them. The manuscript has been revised accordingly and the corrections made in the manuscript are indicated in boxes following each comment.

We hope that this revised version satisfactorily addresses the issues raised.

Yours sincerely,

Mathilde CHEN, Nicolas GUILPART, and David MAKOWSKI

1. **Reviewer #1**

1.1. This article has potential, and I agree with the main thesis that maize/soybean intercropping has great potential for boosting self sufficiency for soybean and maize, but the problems in presenting results in a clear manner persist in this version. I had difficulty in correctly interpreting the abstract and results, though I had little trouble with the introduction, discussion and methods sections which are clear. I feel readers need to be told upfront that total land area in the EU is about 100 Mha, of which 1 Mha is soybean and 9 Mha is maize. Readers need to know upfront whether yields are seed yields or biomass yields (maize may be harvested as both), and if this is not relevant, that needs to be stated and it needs to be explained why it does not matter. Proposed planting areas of intercrops are huge (up to 25 Mha; 25% of the EU arable land area) and it is not clear whether this area increase is from expansion of the arable land area, from partially reducing maize sole cropping (which would make sense) or from replacing another crop (e.g. wheat). Maybe the authors consider this a side issue, but without telling readers, readers cannot well contextualize results. I thus feel further modifications are needed.

Authors might include a table detailing soybean, maize and intercrop areas for different scenarios along with the production of soybean and maize, and include this in the results section. Doing so they may remove many numbers from the narrative and focus the narrative more on the logic and ideas. This could help to make the results section more accessible.

Our response: Thank you for this summary of your comments and feedbacks. We have addressed each of them in this letter.

ABSTRACT

I wrestled with the abstract. I found it left out critical information on the area and production of maize which made it difficult to assess the findings. Specifically:

1.2. 14-17 “Increasing domestic production of one of these species could reduce the production of the other. We examine the potential for maize-soybean intercropping to improve the European Union's soybean self-sufficiency while minimizing competition with maize and other crops.” Maybe rephrase as “while minimizing trade-offs with THE PRODUCTION OF maize and other crops”.

Our response: The sentence was rephrased as suggested by the reviewer (line 16).

1.3. 17-20 “Assuming a one-in-four years frequency, 25%, 50%, and 75% self-sufficiency would be achieved with 6.3, 13.1, and 20.1 Mha of maize-soybean intercropping, resulting in total co-production

of 37.9, 81.9, and 126.2 Mt of both crops, respectively." This is confusing as written as the previous sentence was emphasizing self-sufficiency for soybean, so this sentence would then be about 25, 50 and 75 self-sufficiency for SOYBEAN, but then it goes on to mention the TOTAL grain yields of the two species together. Is this what is meant? To avoid ambiguity, this might be rephrased as "Assuming a one-in-four years frequency, 25%, 50%, and 75% self-sufficiency FOR SOYBEAN would be achieved with 6.3, 13.1, and 20.1 Mha of maize-soybean intercropping, resulting in total co-production of 37.9, 81.9, and 126.2 Mt of both crops, respectively." Still this does not inform the reader what this means for the sufficiency of maize and other crops production and the area of land used for intercropping + maize in the scenarios compared to the area of land used for soybean + maize under current conditions.

Our response: The abstract now explicitly mentions the areas allocated to intercrops, as well as sufficiency levels and productions for both soybean and maize estimated for all three scenarios.

Abstract (lines 16-19):

"Assuming a return every four years, intercropping on 6.3, 13.1, and 20.1 Mha produces 9.1 (28.8), 18.2 (63.7), and 27.2 (99.0) Mt of soybean (maize), respectively, thus achieving 25% (34%), 50% (75%), and 75% (116%) self-sufficiency levels, respectively."

1.4. 21 intercropping -> intercrops

Our response: Corrected (line 21).

1.5. 17-23 I feel lines 17-23 do not give readers a clear picture of the soybean and maize production under the depicted scenarios of 25, 50 and 75% soybean self sufficiency, and the corresponding soybean and maize areas required to reach the same production of the two crops. I realize that this might require more space, but then perhaps the message needs to be simplified.

Our response: See our response to comment 1.3.

1.6. 21-24 "To achieve comparable levels of co-production, separate cultivation of the two species would require an additional 36.5, 24.7, and 11.1 Mha of land, corresponding to an estimated land saving of 20–21% under intercropping." Readers cannot judge the relevance of increases in land area of 36.5, 24.7 and 11.1 Mha of land as the reference areas have not been provided. The percentage land saving makes sense, but likely follows directly from the LER of maize/soybean intercropping. Then the

greater conclusion is that the LER obtained from field-level studies extrapolates well to continent level land use efficiency, even if geographic variation in the water limited potential yields of maize and soybean is taken into account.

Our response: Thank you for this comment. Our study estimates that 20-21% of land could be saved under maize-soybean intercropping depending on the considered scenario (see the new Table 1 of the revised manuscript). According to a meta-analysis of field-scale experiments (Xu et al., 2020), pLERs of soybean and maize would reach 0.56 and 0.79, respectively, corresponding to an average LER of 0.56+0.79=1.35. Under these estimates of land-use efficiency, 26% of land would be saved by maize-soybean intercropping (computed as: $\frac{1-1.35}{1.35}$).

This shows that land savings under maize-soybean intercropping estimated in the present study are not aligned with the pLER and LER estimated from field experiments. This difference between field-scale and continental-scale estimates suggests that these coefficients derived from field experiments ignores the spatial variability in maize and soybean yields (see the detailed analysis of the productive performance of intercropping in case of spatial variability of yield in Supplementary Material 1). It supports the need to take into account this variability when assessing land-use efficiency at the scale of a continent.

We agree that these results are important and we now mention them in the abstract and in the Discussion as well.

Abstract (lines 19-26):

“Reaching similar co-productions with sole crops require an additional 11.1, 24.7, and 36.5 Mha, corresponding to land savings of 20–21% with intercrops. […] Expected land-savings estimates suggest that geographic variation in crop yields should be considered to estimate land-use efficiency of intercrops.”

Discussion (lines 282-292):

“For instance, to simultaneously produce 18.1 Mt of soybean and 63.7 Mt of maize, intercropping required 3.4 Mha (21%) less land than sole cropping, corresponding to an area slightly higher than the combined agricultural areas of Belgium and the Netherlands (1.4 and 1.8 Mha, respectively) (Eurostats, 2026). This land saving estimate differs from the value obtained by direct extrapolation from the pLER values. Indeed, assuming average soybean and maize pLERs of 0.56 and 0.79,22 respectively, expected land savings based on field-scale experiments would be 26% as compared to the 20-21% estimated here. This difference demonstrates that pLER values derived from field-scale experiments cannot be robustly extrapolated to continental-scale land-use efficiency, because geographic variation in actual yields of maize and soybean need to be accounted for (see

Supplementary Material 1 for a detailed explanation of how spatial yield variations affect estimates of land savings)."

References:

Eurostats. Utilised agricultural area by categories. https://doi.org/10.2908/TAG00025 (assessed on: 2026).

Xu, Z. et al. Intercropping maize and soybean increases efficiency of land and fertilizer nitrogen use; A meta-analysis. Field Crops Research 246, 107661, doi:https://doi.org/10.1016/j.fcr.2019.107661 (2020).

1.7. 24-25 A pLER > 0.5 may be hard to achieve for soybean in an intercrop with maize if both species are grown simultaneously as maize is highly competitive in such intercrops. This is an important caveat.

Our response: Thank you for raising this important point regarding the potential difficulty of achieving a soybean pLER > 0.5 in simultaneous intercrops with maize due to maize's strong competitive ability.

Evidence from Xu et al. (2020) meta-analysis shows that in maize-soybean intercropping, at the global scale, mean pLER values are 0.56 for soybean and 0.79 for maize, corresponding to a total LER = 1.35. In comparison, experimental datasets collected exclusively in Europe report higher overall productivity (LER = 1.48 ± 0.087). Because our analysis relies on the global estimate, our assumptions can be considered conservative relative to European conditions. We also highlight that the reviewer did not provide any reference to support the statement that achieving a soybean pLER > 0.5 is difficult, thereby hampering a meaningful comparison with our estimates (which are based on best available evidence to our knowledge).

Importantly, using the relationship between soybean pLER and temporal niche differentiation (TND) reported in the same study, we explicitly evaluated a scenario where both crops are grown simultaneously. This resulted in a soybean pLER of 0.53 (Supplementary Table 4), which remains above the 0.5 threshold and yields results consistent with our main findings.

In addition, our sensitivity analyses also include less favorable scenarios where soybean pLER falls below 0.5. While these scenarios lead to lower gains in soybean self-sufficiency, they still represent a substantial improvement over the current level (16%) as 50% soybean self-sufficiency can be achieved with a soybean pLER of 0.31 assuming that return frequency higher than 1/4 year and that 25 Mha is allocated to maize-soybean intercropping (Figure 3).

We acknowledge that the reported potential of intercropping is conditional on assumed pLER values and for these reasons, we state in the abstract that our conclusions are robust provided soybean

pLER exceeds 0.5, while also demonstrating that meaningful benefits persist even under more conservative assumptions.

> **Abstract (lines 22-25):**
>
> "These estimates held under a large range of nitrogen inputs and temporal overlaps between crop species, provided that soybean production from 1 hectare of intercropping exceeded that from 0.5 ha of sole soybean."
>
> **Discussion (lines 295-300):**
>
> "While intercropping scenarios characterized by pLERs values higher than 0.5 for soybean and 0.7 for maize lead to higher improvements in self-sufficiency and land savings, our results suggest that intercropping maize and soybean in the EU remains an interesting option to improve self-sufficiency compared to sole crops even with lower pLERs. As an example, the level of 50% soybean self-sufficiency is reached as long as soybean pLER is higher than 0.3, assuming that 25 Mha is allocated to maize-soybean intercropping (Figure 3)."

Reference: Xu, Z. et al. Intercropping maize and soybean increases efficiency of land and fertilizer nitrogen use; A meta-analysis. Field Crops Research 246, 107661, doi:https://doi.org/10.1016/j.fcr.2019.107661 (2020).

INTRODUCTION

1.8. 35-36 To set the stage clearly, it would be useful to mention here both the soybean and maize areas in the EU and the average yields and production obtained.

Our response: Following the reviewer's suggestion, we added information on maize and soybean production in the EU.

> **Introduction (lines 34-38):**
>
> "Maize and soybean, two crops primarily used for livestock feed (European Commission, 2024; Debaeke et al., 2022), are cultivated in the EU on 8.9 and 1.0 Mha, respectively (2018-2022 averages) (FAOSTATS, 2026). However, domestic productions - 66.5 and 2.7 Mt (FAOSTATS, 2024a) - falls short of supply quantities of 85.1 and 36.3 Mt, respectively (FAOSTATS, 2024)."

References:

European Commission. Cereals, oilseeds, protein crops and rice. Protection of EU farmers and the agricultural sector through policy on market intervention, trade measures, legislation, and monitoring the market. https://agriculture.ec.europa.eu/farming/crop-productions-and-plant-based-products/cereals_en *(2024).*

Debaeke, P., Tibi, A., Forslund, A., Guyomard, H. & Schmitt, B. Could domestic soybean production avoid Europe's protein imports in 2050? OCL Oilseeds and fats crops and lipids 29, 38 (2022).

FAOSTATS. Crop and livestock products (ed FAO) https://www.fao.org/faostat/en/#data/QCL *(accessed on: 2026).*

FAOSTATS. Food Balances (2010-) (ed FAO) https://www.fao.org/faostat/en/#data/FBS *(assessed on: 2024).*

1.9. Generally the introduction is clear and sets the stage well. The workflow in the last paragraph is clear.

Our response: Thank you for this positive feedback.

RESULTS

1.10. 99-102 "The 25th, 50th (i.e., median), and 75th percentiles of averages soybean yield projections for 2000-2023 across the EU are 1.0, 2.1, and 2.6 $t.ha^{-1}$, respectively (Figure 1a, top panel). These levels of productivity are reached over 103, 82, and 36 Mha, respectively (Figure 1a, bottom panel), where wheat, maize, and barley are the major cultivated crops (Supplementary Table 2)." This sentence maybe misunderstood easily. I first though the percentiles were for the average yield estimate across the whole EU (a single number for the whole continent). And then it made no sense that corresponding areas were mentioned. Upon rereading several times, I gathered that you estimated yields for each grid cell and obtained a distribution across the whole EU for which the percentiles are given. Please rephrase to remove the ambiguity in the term "average yield".

Our response: Following the reviewer's comment, we rephrased this paragraph to mention clearly that we computed the yields for each grid cell and obtained a distribution across the whole EU.

**Results (lines 103-106):**<br><br>"We examine the suitability of soybean production in the EU using grid-cell-wise mean annual rainfed yield projections over 2000-2023. The 25th, 50th (i.e., median), and 75th percentiles of

soybean productivity across the EU are 1.0, 2.1, and 2.6 $t.ha^{-1}$ (Figure 1a, top panel), reached over 103, 82, and 36 Mha, respectively (Figure 1a, bottom panel)."

1.11. 105-107 For context you might mention here the total arable land area in the EU, so readers can immediately see that the 103 Mha is more than 25% of the total arable land area in the EU, actually, even more than the WHOLE arable land area in the EU.

Our response: We thank the reviewer for highlighting this point. According to the definition of cropland in the FAOSTAT Glossary (FAO, 2016), cropland areas include arable lands and permanent crops. Based on Eurostats data, cropland areas cover 109 Mha across the EU. The manuscript now explicitly mentions what "cropland" refers to.

Results (lines 109-112):

"Considering [the] threshold [of 1.0 $t.ha^{-1}$] as a criterion for soybean suitability, our projections suggest that nearly all European cropland areas (i.e., 109 Mha [Eurostats, 2026] including arable lands and permanents crops [FAO, 2016]) could be considered as suitable for soybean production, greatly exceeding the current production area of 1 Mha (Supplementary Table 1)."

References:

Eurostats. Utilised agricultural area by categories. https://doi.org/10.2908/TAG00025 (assessed on: 2026).

FAO, FAOSTAT Glossary, http://faostat3.fao.org/mes/glossary/E (2016).

1.12. 142 "maize's productivity remains above zero" What do you mean? Of course maize's productivity is above zero. Do you mean the change in production? You want to minimize trade-off?

Our response: This was mentioned because the estimated maize yield remains above 0 $t.ha^{-1}$ in the region characterized by higher soybean productivity, where intercropping will be preferably allocated in the tested scenarios. However, we set our allocation procedure such as maize production would be computed even if rainfed yield would be of 0 $t.ha^{-1}$ (produced maize would just be 0 t). We acknowledge that the sentence is confusing and we removed it from the revised text since it does not change the meaning of the paragraph (lines 149-150).

1.13. 149-152 “This system would produce 18.2 Mt of soybean and 63.7 Mt of maize (Figure 3c), representing nearly a sevenfold increase in current EU soybean production while maintaining maize production at nearly the same level (Supplementary Table 1).” For the reader it is not clear whether intercropping in this scenario is replacing sole maize or something else.

Our response: As indicated in the first section of the Results, wheat, maize, and barley dominate the regions identified as suitable for rainfed soybean expansion. However, because maize is another spring crop with similar ecological niche than soybean, maize is the most plausible candidate for substitution through soybean cultivation. We revised the manuscript to make this clearer for the reader.

Results (lines 114-117):

“Dominant crops currently cultivated in soybean-suitable area are wheat, maize, and barley (Supplementary Table 2). Maize shares a similar ecological niche with soybean (Mahaut et al., 2022), suggesting that it is the most likely to be replaced as soybean area expands.”

Reference: Mahaut, L. et al. Matches and mismatches between the global distribution of major food crops and climate suitability. Proceedings of the Royal Society B: Biological Sciences 289, doi:10.1098/rspb.2022.1542 (2022).

1.14. 144-155 To be more comprehensive in the presentation of results, it might be beneficial to present for each scenario soybean area, maize area, intercrop area, and the associated soybean and maize production. Somewhat in line with Supplementary Table S3. This would greatly facilitate the insightfulness of the results. It would also clarify to which extent maize or other crop species are replaced. Such a table would have to be in the main text, not in supplementary. When the table is included, the text can be simplified because readers can see the numbers in the table.

Our response: We thank the reviewer for this helpful suggestion. We agree that presenting all results in a table provides a clearer and more comprehensive overview of area requirements, soybean and maize production, and land savings under intercropping across the different scenarios (i.e., 25, 50, 75, and 100% soybean self-sufficiency).

Following this comment, Supplementary Table 3 has been included as Table 1 in the revised manuscript. The former Figure 3 has been retained as Supplementary Figure 10, as it provides valuable spatial information on the geographical distribution of intercropping, sole crops, and the additional land required to produce equivalent amounts of maize and soybean under both cropping systems.

1.15. 156-171 The narrative in this paragraph would be supported by including yet another scenario in the 50% self sufficiency for soybean is achieved by increasing soybean area (at the expense of maize?) and again reporting soybean and maize cropping areas as well as the soybean and maize production. The maps are nice, but they are not a good vehicle to support the text on area requirements to reach certain levels of sufficiency. In line with previous comment: a table is needed.

Our response: Please see our response to comment 1.14.

1.16. 189-193 This is a best case; be cautious.

Our response: The paragraph has been rephrased to take into account that our estimates were made in an optimistic scenario.

Results (lines 194-197):

"In an optimistic scenario where the total intercropping area could be as high as 25 Mha, our estimations indicate that maximum self-sufficiency levels of 91% for soybean and at least 145% for maize could be achieved with a conventionally managed intercropping system (i.e., one-in-four years cultivation, with a soybean pLER of 0.56 and a maize pLER of 0.79) (Figure 3)."

1.17. 211 A soybean pLER of 0.53 with TND = 0 seems optimistic. It maybe in line with the results of Xu et al. (2019) but I am not sure. Maybe those data would need to be analysed specifically for pLERs of both soybean and maize with both higher N input and low TND (also because of mechanization difficulties if TND>0). Are the data of Xu et al. (2019) available from public sources or from the authors?

Our response: We managed to get the data associated with the meta-analysis of Xu et al. (2020). However, the data does not include information for pLER, so it was not possible to directly derive the pLERs associated with a TND=0. In this meta-analysis, the average LER provided was 1.32, with pLERs values of 0.56 and 0.79 for soybean and maize, respectively. Additionally, the authors report a significant relationship between LER and TND with a slope of 0.214 ± 0.011 (units of LER per unit of TND) and an intercept (LER at TND = 0) of 1.25 ± 0.03. We computed the ratio between each individual pLER and average LER and we used them to weight the LER at TND = 0 to derive corresponding pLERs. We ended with pLERs values of 0.53 and 0.76 for soybean and maize, respectively. This procedure is fully detailed in Supplementary Material 5.

1.18. 224-226: The truth of this statement depends on whether intercropping replaces maize or another crop. If intercropping replaces maize, then less intercropping would mean greater self sufficiency for maize production in the EU; however, if intercropping replaces another crop than maize, less intercropping indeed would mean lower self sufficiency for maize production in the EU but if would imply higher self sufficiency for the unnamed crop that is replaced by intercropping. It depends on what crop is replaced by intercropping; but that is not clear. I feel a table with clear scenario assumptions (as suggested above) could help in this respect.

Our response: The present statement is not related to the overall maize production but to the companion maize which is cultivated with soybean in intercropping. We rephrased the sentence to clarify this.

Results (lines 228-230):

"[…] less land is required to meet the soybean self-sufficiency target, thus reducing the production of companion maize associated to soybean in intercropping […]."

1.19. 241-243 "Interestingly, 50% soybean self-sufficiency and 100% maize self-sufficiency would be simultaneously achieved a maize pLER of 0.79 as soon as soybean pLER remains lower than 0.41." This result is not logical. If the pLER of soybean decreases, while that of maize stays the same, a greater area of intercropping is needed to match a given production target of soybean. Then the self sufficiency of maize is bound to change, getting lower if intercropping replaces sole maize and getting higher if intercropping replaces another crop than maize. Maybe you mean AT LEAST 100% maize self-sufficiency. Please rephrase for clarity.

Our response: We rephrased the sentence accordingly (lines 246-248).

DISCUSSION

1.20. 311 Sorting of grain is indeed needed if the crops are harvested as bulk. In China, where maize/soybean intercropping is practiced and advocated by the national government, the crops are harvested separately, using specific equipment. See Li Chunjie et al. (2020) in Nature Plants. Li Long and colleagues at China Agricultural University investigate wide strip maize/soybean intercropping to facilitate separate harvesting with bigger than traditional equipment as shown in Li et al. (2020). In colder areas (e.g. Tibet) encouraging results have been obtained with using maize/soybean silage (Yang Wenyu, personal communication). Li CJ, Hoffland E, Kuyper Th W, Yu Y, Zhang CC, Li HG, Zhang FS, van der Werf W (2020) Syndromes of production impact yield gains in intercropping. Nature Plants 6, 653–660. http://dx.doi.org/10.1038/s41477-020-0680-9

Our response: Thank you for this information. This is now mentioned in the revised manuscript.

**Discussion (lines 326-327):**<br><br>“Machinery is already available for intercropping, especially in China (Li et al., 2020), but further development is required to facilitate their large-scale deployment.”

Reference: Li CJ, Hoffland E, Kuyper Th W, Yu Y, Zhang CC, Li HG, Zhang FS, van der Werf W (2020) Syndromes of production impact yield gains in intercropping. Nature Plants 6, 653–660. http://dx.doi.org/10.1038/s41477-020-0680-9

1.21. 351-353 “Our findings suggest that to achieve 91% soybean self-sufficiency, more than 25 Mha of maize-soybean intercropping would be required, corresponding to an increase of 15 Mha compared to the current harvested areas of maize and soybean in the EU.11” From this I would calculate the current maize area in the EU is 25 – 1 – 15 = 9 Mha. But if that is true, the planting of 15 Mha of intercropping would result in a big overproduction of maize in the EU. This would also suggest further adaptations to the implemented scenarios to avoid such overproduction?

Our response: We thank the reviewer for this insightful comment and agree that this point deserves clarification. Some scenarios examined in this study can indeed lead to situations where maize production exceeds current EU levels, due to the additional maize produced within intercrops. Such additional production could be used to improve the current level of maize self-sufficiency of 81% or toward increased exports, which may represent a potential economic opportunity rather than a limitation.

Nevertheless, we agree with the reviewer about the possible optimization of maize-soybean intercropping scenarios to better balance co-productions. As shown in our main analysis (Figure 3) and sensitivity analyses (Supplementary Figures 12, 13-16, 17, and 19), modifying parameters such as intercropping return frequency and the pLER of maize allows for reducing maize output while maintaining substantial gains in soybean production compared to the current situation.

We have included a sentence in the discussion section of the revised manuscript to clarify this point and avoid potential misinterpretation.

**Discussion (lines 387-394):**<br><br>“Our results suggest that the widespread adoption of maize-soybean intercropping in the EU would improve soybean self-sufficiency (25- 91%, depending on the considered scenario) but also contribute to cover maize consumption in the EU (50-145%, depending on the considered scenario). For maize, the levels attained could even be higher considering the maize produced

outside of the intercropping area, thus increasing EU's maize self-sufficiency and exports. However, our sensitivity analyses suggest that the design of intercropping systems could be adjusted (especially by tuning intercropping return frequency, relative crop densities, and maize pLER) to limit maize overproduction while still achieving high levels of soybean self-sufficiency."

METHODS

1.22. 384-392 Please clarify whether yields are seed yields (standard moisture percentage) or seed yield dry matter, or biomass (fresh or after drying).

Our response: In this study, we considered grain yields (standard moisture percentage). This is now specified in the Results (line 90) and Material and Methods section (line 414).

1.23. 432-440 Please clarify which land is allocated to intercropping. Total arable land area in the EU currently is about 100 Mha, of which 1 Mha is soybean and 9 Mha is maize. Scenarios for increased self sufficiency for soybean result in a major area allocation to soybean/maize intercropping, up to 25 Mha for reaching 91% soybean self sufficiency. The text in 432-440 mentions that arable land area increase is avoided, and competition with maize growing area is also avoided. Somehow, it is not clear where the land is coming from, and it may even be questioned if the land area is available.

Our response: Under some of the scenarios considered, the intercropping area required to achieve the targeted level of soybean self-sufficiency exceeds the current combined area of sole soybean and maize in the EU, i.e., 1 + 9 = 10 Mha (2018-2022 averages), based on FAOSTATS (2026). To avoid allocating an excessively large and unrealistic amount of land to intercropping, we constrained our allocation procedure by limiting additional cropland allocated to intercropping to 15 Mha, resulting in a maximum total intercropped area of 25 Mha (10 + 15) at the EU scale. We improved the Material and Methods section to reflect that assumption in our allocation procedure.

We acknowledge that this assumption may imply substantial competition with other crops, particularly in high-productivity areas for soybean (see our response to comment 1.13). However, the primary objective of this study is to quantify the land requirements for maize–soybean intercropping to achieve EU soybean self-sufficiency; identifying which specific crops would be displaced is beyond the scope of the present analysis. This limitation and its implications are now explicitly discussed in the revised manuscript.

Discussion (lines 373-386):

“Assuming that 1 ha of intercropping produces as much as 0.56 ha of sole soybean and 0.79 ha of sole maize, our results suggest that 13.1 Mha of intercropping are required to meet 50% soybean self-sufficiency in the EU (Table 1). This area is only 30% larger than the 10 Mha currently devoted to the cultivation of maize and soybeans in the EU (Supplementary Table 1). However, achieving higher soybean self-sufficiency levels through intercropping expansion could have a more important impact on land use in the EU. Our findings suggest that, in the same conditions of land use efficiency (pLER of 0.56 and 0.79 for soybean and maize, respectively), 25 Mha of maize-soybean intercropping would be required to achieve 91% soybean self-sufficiency. This could be achieved by growing maize-soybean intercrops on the current harvested area of maize and soybean in the EU plus an additional 15 Mha currently cultivated by other crops, mostly wheat, barley rapeseed and sunflower (Supplementary Table 2). In this scenario, the pressure on cropland will increase, but it is difficult to determine which crops are most likely to be replaced by corn-soybean intercrops due to the influence of numerous factors, including future supply-demand balances and future commodity prices (Meyfroidt et al 2022).”

Material and methods (lines 472-482):

“Our allocation process was guided by three assumptions: (1) maize-soybean intercropping would be preferentially implemented in the cropland areas with the highest projected soybean yields but (2) excluding sites with unsuitable conditions for soybean production (i.e., projected yields below 1 t.ha-1, corresponding to the minimum observed in the training dataset from the US and Italy; Supplementary Figure 21); (3) we assumed that intercropping would first replace existing maize and soybean areas (i.e., 10 Mha including 1.0 Mha of soybean and 8.9 Mha of maize, 2018–2022 averages [FAOSTATS, 2026]). If the soybean self-sufficiency targets were not met, additional cropland could be allocated up to 15 Mha to limit competition with other crops. In that case, the total intercropped area at the scale of the EU would be capped at 25 Mha, an area equivalent to 23% of the EU’s cropland (i.e., 109 Mha) (Eurostats, 2026a) and comparable to the current European wheat area in 2020 (Eurostats, 2026b).”

References:

Eurostats. Utilised agricultural area by categories. https://doi.org/10.2908/TAG00025 (assessed on: 2026a).

Eurostats. Farms and hectares by crops, utilised agricultural area, economic size of the farm and NUTS 2 region. https://doi.org/10.2908/EF_LUS_ALLCROPS (assessed on: 2026b)

FAOSTATS. Crop and livestock products (ed FAO) https://www.fao.org/faostat/en/#data/QCL (accessed on: 2026).

Meyfroidt, P., De Bremond, A., Ryan, C. M., Archer, E., Aspinall, R., Chhabra, A., ... & Zu Ermgassen, E. K. (2022). Ten facts about land systems for sustainability. Proceedings of the National Academy of Sciences, 119(7), e2109217118.

• What are the noteworthy results?
Maize/soybean intercropping, if applied at a large scale in Europe, has potential major benefit for self sufficiency for both maize and soybean in the EU. This is not a surprising result, but this paper is the first to come up with somewhat credible quantification.
• Will the work be of significance to the field and related fields? How does it compare to the established literature? If the work is not original, please provide relevant references.
The work is important as a hallmark in quantification of the benefits of intercropping in Europe if applied at a large scale. It may make policy makers aware of the need to invest in research and technological development to make intercropping of maize and soybean technically achievable and known and acceptable to farmers in the EU.
• Does the work support the conclusions and claims, or is additional evidence needed?
Yes, the conclusions are supported. I agree with the conclusions and the analysis, but found the presentation needs improvement to enhance its clarity and transparency (see above).
• Are there any flaws in the data analysis, interpretation and conclusions? Do these prohibit publication or require revision?
I don't think there are important flaws. There are uncertainties, but these are acknowledged.
• Is the methodology sound? Does the work meet the expected standards in your field?
I am satisfied.
• Is there enough detail provided in the methods for the work to be reproduced?
Yes.

2. **Reviewer #3**

2.1. Again, I thank the authors for their careful revision. The manuscript has improved in clarity, structure, and transparency. In particular, the description of the allocation procedure is now clearer (e.g., Figure 2 and corresponding text), and additional sensitivity analyses have been conducted to explore the robustness of the results to variations in pLER values and key drivers such as nitrogen input and temporal niche differentiation.

Our response: We thank the reviewer for this positive feedback on the revision of our manuscript.

However, my main concern remains only partially addressed.

2.2. The central focus of the manuscript is the efficiency of intercropping, which is quantified through the land equivalent ratio (LER). As noted previously, LER is not a fixed parameter but a dynamic outcome of interacting factors including climate, soil conditions, and management practices (e.g., fertilization, irrigation, and relay versus simultaneous intercropping). While the authors acknowledge this and perform sensitivity analyses across a wide range of pLER values, LER remains treated as an exogenous parameter rather than being explicitly linked to the same drivers that determine crop yields in the model.
In the current framework, crop yields are spatially explicit and climate-driven (via the machine learning model), whereas intercropping performance is imposed through externally derived pLER values that do not vary consistently with environmental conditions. This creates a structural inconsistency: the model captures spatial variability in sole crop productivity but not in the intercropping advantage itself. As a result, the estimated land-use efficiency and land-saving potential depend critically on assumptions about LER that are not mechanistically or statistically integrated into the modeling framework.
The additional sensitivity analyses are useful and demonstrate that the main conclusions are numerically robust across a range of assumed pLER values. However, they do not fully resolve the underlying issue that LER may co-vary with climate and management conditions across Europe. Therefore, the results should be interpreted as conditional on the assumed distributions of pLER, rather than as fully spatially resolved estimates of intercropping performance.

Our response: Thank you raising these important points. We have answered them in the following pages and revised the manuscript accordingly. See our responses in comments 2.3, 2.4, and 2.5.

I encourage the authors to further clarify this point in the manuscript. In particular:

2.3. The limitations section should more explicitly state that LER is not modeled as a function of environmental drivers and that this may affect spatial allocation results.

Our response: As suggested by the reviewer, the revised manuscript now explicitly mentions this limitation in the discussion section.

> **Discussion (lines 366-372):**
>
> "In absence of experimental datasets that explicitly explore variations in intercropping efficiency as a function of weather conditions, it was not possible to predict the effect of environmental factors on pLERs and, thus, take the potential spatial variability of pLERs into account. The establishment of a shared, standardized, and open-access global database on intercropping systems—including detailed information on management, environmental conditions, and LER outcomes—would represent a major advance to address this issue."

2.4. The framing of the conclusions could be adjusted to emphasize that the reported potential of intercropping is conditional on assumed pLER values.

Our response: We acknowledge that the reported potential of intercropping is conditional on assumed pLER values and for these reasons, we state in the abstract that our conclusions are robust provided soybean pLER exceeds 0.5, while also demonstrating that meaningful benefits persist even under more conservative assumptions.

> **Abstract (lines 22-25):**
>
> "These estimates held under a large range of nitrogen inputs and temporal overlaps between crop species, provided that soybean production from 1 hectare of intercropping exceeded that from 0.5 ha of sole soybean."
>
> **Discussion (lines 295-300):**
>
> "While intercropping scenarios characterized by pLERs values higher than 0.5 for soybean and 0.7 for maize lead to higher improvements in self-sufficiency and land savings, our results suggest that intercropping maize and soybean in the EU remains an interesting option to improve self-sufficiency compared to sole crops even with lower pLERs. As an example, the level of 50% soybean self-sufficiency is reached as long as soybean pLER is higher than 0.3, assuming that 25 Mha is allocated to maize-soybean intercropping (Figure 3)."

2.5. If feasible, even a simplified stratification of pLER values across agro-climatic zones would strengthen the consistency of the framework.

Our response: We thank the reviewer for suggesting this analysis, which is currently not feasible

given the lack of data points available by climate area (see Supplementary Figures 23-24). As mentioned in the discussion section, we highlight the need of data from field experiments to finely describe the relationship between intercropping efficiency and climate.

**Discussion (lines 315-319):**<br><br>“Given the limited information on the interactions between soybean productivity in intercropping and other farming practices in Europe, additional trials are required to refine local estimations of maize-soybean intercropping performances and to help identifying the best combinations of field configuration, fertilization, and water management which maximizes the productivity of intercropping at the farm level.”

2.6. Overall, the manuscript addresses an important question and has been substantially improved. Nevertheless, the treatment of LER remains a key limitation that should be more explicitly acknowledged and discussed to ensure that the conclusions are interpreted appropriately.

Our response: We thank the reviewer for this comment. As answered to comments 2.3 and 2.5. this limitation regarding LER treatment and the need for additional data from field studies is now included the revised manuscript (lines 315-319 and 366-372 of the Discussion section).

Remarks on code availability:

2.7. The codebase reflects solid scientific programming and implements the paper’s core methodology effectively, particularly the spatial allocation framework for maize–soybean intercropping, the random forest setup, the PCA-based climate preprocessing, and the baseline use of partial land equivalent ratios for soybean and maize.

Our response: Thank you for this positive feedback.

2.8. At the same time, its present form limits reproducibility and broader community use: several scripts rely on system-specific file paths, the large raw input datasets are not included in the repository even though their sources are documented, some sensitivity-analysis scripts described in the paper are missing, and the README would be stronger with clearer installation guidance, dependency specifications, data access instructions, and troubleshooting notes. The documentation does provide a useful overview of the workflow and data provenance, but reproducing the full analysis still requires substantial setup and adaptation. Even so, the cached outputs appear sufficient to regenerate the final

figures with limited effort, and the main workflow should be reproducible with file-path revisions, appropriate data organization, and a better-defined software environment.

Our response: The code reproducibility has been improved following the reviewer's suggestions. In particular:

- system-specific file paths were removed from the scripts to facilitate setup and adaptation to a new coding environment,
- all climate inputs and yield predictions used in this study are now provided in addition to their source, in other to improve the reproducibility of our simulations,
- all the scripts developed in this study, including the scripts performing sensitivity analyses, are now provided,
- the README file has been strengthened with session information on the computer, software, and packages versions used to perform the analyses.

The public GitHub repository (https://github.com/MathildeChen/Maize_Soybean_Intercropping_Europe) has been updated with these new features.

2.9. Overall, this is a well-constructed and scientifically valuable codebase whose reproducibility and community uptake would be improved by platform-independent paths, more explicit setup documentation, and packaging tools such as dependency locking or containerization.

Our response: Thank you for this positive feedback. The changes performed according to reviewer's suggestions in comment 2.8. improved the reproducibility of our analyses by the whole community.

Manuscript reference: NCOMMS-25-06543-C entitled “High potential contribution of intercropping to soybean and maize self-sufficiency in Europe”

RESPONSE LETTER TO REVIEWER COMMENTS

June 30th 2026

Dear reviewers,

We thank you for your advised recommendations during this review process.

Please find below the response to each of the comments on the manuscript, which has been revised accordingly. The corrections made are indicated in boxes following each comment.

We hope that this revised version satisfactorily addresses the issues raised.

Yours sincerely,

Mathilde CHEN, Nicolas GUILPART, and David MAKOWSKI

REVIEWERS' COMMENTS

1. Reviewer #1 (Remarks to the Author):

1.1. Authors provide a convincing analysis that use of maize/soybean intercropping will contribute substantially to soybean and maize self sufficiency in Europe if applied at a large scale. Results extend the meta-analysis results of Xu et al. (2021) and provide a compelling analysis of potentials of this intercropping system in the EU. The results are important as they progress from existing LER values, which are abstractions, to economic potentials, which will probably trigger more the imagination of policy makers and other stakeholders. Hence, this study could have high impact. The machine learning values for yield projections help give credibility to the results. Evidence is sufficient, and there is ample analysis of uncertainties. I have not seen any flaws. Methods are sound and are at the level the journal should expect. There is a lot of information in supplementary, and I understand that codes are provided (but I did not check those). There are still some minor issues in the text that might be clarified. I do provide an annotated word file with minor suggestions for improving clarity and language, at the discretion of the authors.

I have seen previous versions of this ms, and my concerns are sufficiently addressed in this version.

Our response: We thank the reviewer for these positive summary and feedbacks on our study. We are delighted to know that the manuscript now addresses his/her concerns and we thank him/her as well for the suggestions. The majority of these suggestions were taken into account in the revised version of the manuscript. When necessary, we provide complementary information in the present letter.

Introduction section:

1.2. Lines 58: Could or did?

Our response: We acknowledge that the formulation was unclear. The cited paper estimates the impact of intercropping on China's soybean production using 2018 as a reference year. We modified the sentence to reflect the results of the study.

Introduction (lines 59-61):

“Based on production levels in China in 2018, estimates suggest that intercropping maize with soybean on 50% of the maize planting area could more than double the country’s total soybean production (Wu et al., 2023).”

Reference: Wu, Y. et al. Soybean yield variations and the potential of intercropping to increase production in China. Field Crops Research 291, 108771, doi:https://doi.org/10.1016/j.fcr.2022.108771 (2023).

1.3. Line 68: Actually, they account for it by averaging.

Our response: Average yield estimates are indeed derived from observations spanning areas with different productivity levels. Our intention was to emphasize that these estimates do not explicitly represent or quantify the spatial variability in maize and soybean yields. We have revised the sentence to clarify this point.

Introduction (lines 70-72):

“[…] these field-scale estimates do not explicitly represent the spatial variability in maize (Lecerf et al., 2019) and soybean (Guilpart et al., 2022; Rotundo et al., 2024; Nendel et al., 2023) yields across areas of contrasted productivity (e.g., due to climate conditions).”

References:

Guilpart, N., Iizumi, T. & Makowski, D. Data-driven projections suggest large opportunities to improve Europe’s soybean self-sufficiency under climate change. Nature Food 3, 255-265, doi:10.1038/s43016-022-00481-3 (2022).

Lecerf, R., Ceglar, A., López-Lozano, R., Van Der Velde, M. & Baruth, B. Assessing the information in crop model and meteorological indicators to forecast crop yield over Europe. Agricultural Systems 168, 191-202, doi:https://doi.org/10.1016/j.agsy.2018.03.002 (2019).

Nendel, C. et al. Future area expansion outweighs increasing drought risk for soybean in Europe. Global Change Biology 29, 1340-1358, doi:https://doi.org/10.1111/gcb.16562 (2023).

Rotundo, J. L. et al. European soybean to benefit people and the environment. Scientific Reports 14, 7612, doi:10.1038/s41598-024-57522-z (2024).

1.4. Line 162: Please add the value.

Our response: The value of the current self-sufficiency value (i.e., 81%) is already provided at the end of the sentence. We rephrased it to make it clearer.

Results (lines 164-165):

"[…] thereby more than tripling the current self-sufficiency rate for soybean (16%), while slightly reducing the current maize self-sufficiency rate (81%)."

1.5. Line 168: What is residual about it?

Our response: In this sentence, "residual area" refers to the land area remaining after accounting for the difference between the intercropping and sole soybean scenarios. In the revised version of the manuscript, we removed the term and explicitly explained what is these 6.0 Mha of land.

Results (lines 171-172):

"[…] growing sole maize on the 6.0 Mha of land spared under sole soybean scenario could produce 39.0 Mt of maize […]"

1.6. Lines 250-251: Is this a logical result? If soybean pLER increases just a bit above 0.14, one needs a bit less intercropping area to achieve the soybean sufficiency target, and I cannot imagine that then suddenly the maize self sufficiency drops from more than 100 to less than 75%. Please check.

Our response: Indeed, our sensitivity analyses suggest that the maize self-sufficiency could range between 75% and 100% in case of increase of the soybean pLER above 0.41. This is because the land allocated to maize-soybean is higher when intercropping efficiency to produce soybean (i.e., soybean pLER) decreases. We rephrased the sentence to clearly reflect these results.

Results (lines 252-255):

"When soybean pLER exceeds this threshold, less land is required to reach 50% soybean self-sufficiency and the area allocated to intercropping decreases. In response, maize self-sufficiency does not exceed 100% and could drop to 75% under highest soybean pLER values."

1.7. Lines 271-274: Sentence does not run. Please check.

Our response: Following reviewer's comment, we improved the sentence.

Discussion (lines 275-278):

"This study estimates the benefits of intercropping to improve EU's self-sufficiency in soybean while maintaining a high production of maize, and quantifies the land saved by this strategy over sole cropping at a continental scale. It extends the results from previous meta-analysis by taking into account spatial variability of maize and soybean productivity in the EU."

2. Reviewer #3 (Remarks to the Author):

2.1. I thank the authors for their careful revision. The manuscript has improved substantially in clarity and transparency. In particular, the allocation procedure is now much clearer, and the additional sensitivity analyses for pLER values, nitrogen input, and temporal niche differentiation help readers understand the conditional robustness of the conclusions. My main concern regarding the treatment of LER as an exogenous parameter rather than as a modeled function of environmental and management drivers has now been sufficiently acknowledged as a limitation. The revised Discussion appropriately states that spatial variability in pLER could not be modeled due to the lack of suitable experimental datasets, and the conclusions are now more clearly framed as conditional on assumed pLER values. I therefore do not see a remaining critical issue that would prevent publication.
Our response: We thank the reviewer for this positive feedback on the revision of our manuscript and we are very pleased that the manuscript addresses his/her concerns.

I only recommend minor editorial corrections before acceptance:
2.2. Harmonize the description of allocation ranking, which is described as soybean production in the Results but projected soybean yield in the Methods.

Our response: The description of the allocation process has been harmonized through the manuscript.

Results (lines 146-148):

"We sequentially allocate intercropping to the most productive sites, ranked by soybean production (i.e. estimated by multiplying projected sole soybean yield by the cropland area in each site) […]"

Methods (lines 477-481):

"Our allocation process was guided by three assumptions: (1) maize-soybean intercropping is preferentially implemented in the cropland areas with the highest projected soybean production but (2) excluding sites with unsuitable conditions for soybean production (i.e., projected yields below 1 t.ha-1, corresponding to the minimum observed in the training dataset from the US and Italy; Supplementary Figure 21);"

Methods (lines 493-495):

"[…] maize-soybean intercropping was sequentially allocated to highest yielding sites, ranked by soybean productivity (i.e., projected soybean yield multiplied by the cropland area in each site) […]"

2.3. Report land-saving values consistently as positive percentages, or clarify the sign convention in Table 1.

Our response: The percentages of land saved by intercropping are expressed as the absolute value of the relative difference of land use by sole cropping vs intercropping in order to consistently report positive percentages through the manuscript. This is now explicitly stated in the notes of the Table 1.

2.4. Check minor rounding inconsistencies, such as 18.1 vs 18.2 Mt.

Our response: Corrected (see in Discussion section, lines 284 and 288).

2.5. Overall, I am satisfied with the revision and support publication after these minor editorial corrections.

Our response: We thank the reviewer for pointing these minor issues and for the positive feedback.
